# Novel insights into the nervous system affected by prolonged hyperglycemia

**DOI:** 10.1007/s00109-023-02347-y

**Published:** 2023-07-18

**Authors:** Kamila Zglejc-Waszak, Konark Mukherjee, Agnieszka Korytko, Bogdan Lewczuk, Andrzej Pomianowski, Joanna Wojtkiewicz, Marta Banach, Michał Załęcki, Natalia Nowicka, Julia Jarosławska, Bernard Kordas, Krzysztof Wąsowicz, Judyta K. Juranek

**Affiliations:** 1 Department of Human Physiology and Pathophysiology, School of Medicine, Collegium Medicum, University of Warmia and Mazury in Olsztyn, 10-085, Olsztyn, Poland; 2 Fralin Biomedical Research Institute at VTC, Virginia Tech, VA 24016 USA; 3 Department of Histology and Embryology, Faculty of Veterinary Medicine, University of Warmia and Mazury in Olsztyn, 10-719 Olsztyn, Poland; 4 Internal Medicine Department, Faculty of Veterinary Medicine, University of Warmia and Mazury in Olsztyn, 10-719 Olsztyn, Poland; 5 Department of Neurology, Collegium Medicum, Jagiellonian University, 31-008 Krakow, Poland; 6 Department of Animal Anatomy, Faculty of Veterinary Medicine, University of Warmia and Mazury in Olsztyn, 10-719 Olsztyn, Poland; 7 Department of Biological Functions of Food, Institute of Animal Reproduction and Food Research, Polish Academy of Sciences, 10-748 Olsztyn, Poland; 8 Department of Pathophysiology, Forensic Veterinary Medicine and Administration, Faculty of Veterinary Medicine, University of Warmia and Mazury in Olsztyn, 10-719 Olsztyn, Poland

**Keywords:** Spinal cord, Sciatic nerve, Cytoskeleton, Inflammation, Neuropathy, Diabetes

## Abstract

**Abstract:**

Multiple molecular pathways including the receptor for advanced glycation end-products-diaphanous related formin 1 (RAGE-Diaph1) signaling are known to play a role in diabetic peripheral neuropathy (DPN). Evidence suggests that neuropathological alterations in type 1 diabetic spinal cord may occur at the same time as or following peripheral nerve abnormalities. We demonstrated that DPN was associated with perturbations of RAGE-Diaph1 signaling pathway in peripheral nerve accompanied by widespread spinal cord molecular changes. More than 500 differentially expressed genes (DEGs) belonging to multiple functional pathways were identified in diabetic spinal cord and of those the most enriched was RAGE-Diaph1 related PI3K-Akt pathway. Only seven of spinal cord DEGs overlapped with DEGs from type 1 diabetic sciatic nerve and only a single gene cathepsin E (*CTSE*) was common for both type 1 and type 2 diabetic mice. In silico analysis suggests that molecular changes in spinal cord may act synergistically with RAGE-Diaph1 signaling axis in the peripheral nerve.

**Key messages:**

- Molecular perturbations in spinal cord may be involved in the progression of diabetic peripheral neuropathy.
- Diabetic peripheral neuropathy was associated with perturbations of RAGE-Diaph1 signaling pathway in peripheral nerve accompanied by widespread spinal cord molecular changes.
- In silico analysis revealed that PI3K-Akt signaling axis related to RAGE-Diaph1 was the most enriched biological pathway in diabetic spinal cord.
- Cathepsin E may be the target molecular hub for intervention against diabetic peripheral neuropathy.

**Supplementary Information:**

The online version contains supplementary material available at 10.1007/s00109-023-02347-y.

## Introduction

Over years it has become apparent that abnormalities in diabetic peripheral neuropathy (DPN) are caused by several concurrent factors such as: accumulation of advanced glycation end-products (AGEs), receptor of AGEs-diaphanous related formin 1 (RAGE-Diaph1) pathway, axonal transport alteration concomitant with increased inflammation and oxidative stress [1–9]. However, clinical evidence demonstrates that besides peripheral effects, diabetes also affect central nervous system (CNS) neurons, with structural and metabolic alternations observed primarily in spinal cord (SC) [10–15]. Tesfaye and co-workers [10] revealed that high blood glucose level also affects CNS. Hence, we may suppose that long-term hyperglycemia may have an impact on SC transcriptome profile. To date however no study has used high throughput RNA sequencing experiments on SC in animal models of DPN. In this study, using a type 1 diabetes (T1D) mouse model we also carefully tested the timing of DPN initiation and its relation to changes in RAGE-Diaph1 signaling axis in sciatic nerve (SN).

The aim of our study was to investigate diabetes-triggered molecular alternations in SC as well as perturbations in RAGE-Diaph1 axis in SN. We uncovered that DPN initiation may precede altered RAGE-Diaph1 signaling in SN as well as gene expression profile within the SC of T1D mouse model.

## Materials and methods

### Animals

**Fig. 1:**
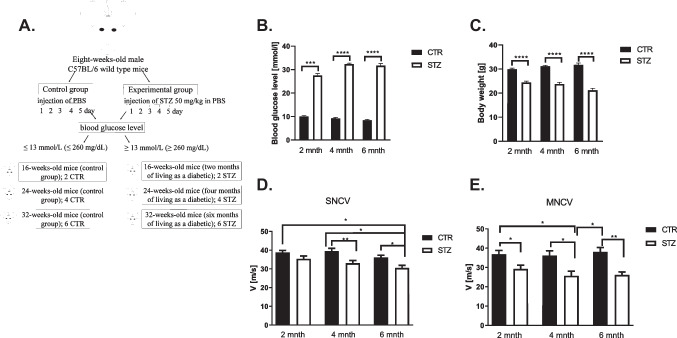
Diabetic neuropathy study. **A**  Experiment diagram **B** The effect of type 1 diabetes (T1D) on glucose level. **C** The impact of T1D on body weight in mice, analyzed by *Kruskal–Wallis* test. Data are expressed as means ± SEM; * *P* ≤ 0.05, ** *P* ≤ 0.01; *** *P* ≤ 0.001; **** *P* ≤ 0.0001; n = 12 mice in each group. **D** Sensory nerve conduction velocity (SNCV) in the diagnosis of diabetic peripheral neuropathy (DPN). For SNCV, the sural nerve was stimulated orthodromically using needle electrodes placed in the fourth toe of the foot, with recording via needle electrodes in the gastrocnemius muscle. Sensory conduction velocity was calculated by dividing the distance between the stimulating and recording electrodes by this latency. **E** The effect of T1D on motor nerve conduction velocity (MNCV). The MNCV was calculated by the dividing in distance between electrodes placed upper thigh near midline at the sciatic notch and electrodes placed in the popliteal fossa (measured with a fine caliper) by a difference in latency during stimulation at the sciatic notch compared with that obtained during popliteal fossa stimulation to yield a velocity in meters per second. The studies were performed using Nicolet Viking Quest Apparatus and Nicolet Viking, version X computerized system (CareFusion, San Diego, CA, USA). One-way ANOVA followed by Tukey's HSD post hoc test was used to indicate the effect of diabetes on alterations in SNCV as well as MNCV. Finally, in case of SNCV and MNCV, two-way ANOVA was performed to evaluate the effects and interactions among factors (diabetes status and duration of diabetes) followed by the Tukey’s HSD post-hoc test. Data are expressed as means ± SEM; * *P* ≤ 0.05, ** *P* ≤ 0.01; n = 12 mice in each group. Abbreviation: CTR – control, STZ – diabetes, mnth – month

All experiments were approved by the Local Ethics Committee of Experiments on Animals in Olsztyn (Poland; decision no. 57/2019). Eight weeks old C57BL/6 males were randomly divided into control and experimental groups per defined time points (Fig. 1A). Streptozotocin (STZ, 50 mg/kg; Sigma-Aldrich, USA) or a vehicle (PBS, pH 7.4; Eurx^®^, Poland) were administered daily for a week (Fig. 1A). Mice were sacrificed eight, 16 and 24 weeks post the last STZ injection (two, four and six months of rendered diabetes, respectively). Animals with a blood glucose level ≥ 13 mmol/L (260 mg/dL) were considered diabetic.

### Nerve conduction velocity (NCV)

Measurement of SN electrophysiological activities was performed using Nicolet Viking Quest Apparatus (CareFusion, San Diego, CA, USA) as previously described [1, 5, 16]. Prior to motor (MNCV) as well as sensory nerve conduction velocity (SNCV) studies, all animals (n = 12; [17, 18]) were anesthetized as described previously [1].

### Morphometric and ultrastructural studies (scanning electron microscope, SEM)

Nerve samples (n = 5 per group; at 6 months of experiment; [19]) were immersion-fixed in a mixture of 1% paraformaldehyde and 2.5% glutaraldehyde in 0.2 M cacodylate buffer (pH 7.4) for 2 h at 4 °C.

### The morphometric and ultrastructural analysis of semithin sections

Semithin sections (n = 5, [19]) were stained with 1% toluidine blue and digitalized at 40× objective using PANNORAMIC 250 Flash III scanner (3DHistech, Budapest, Hungary). Samples for scanning electron microscopy (SEM) imaging were post-fixed in a solution containing 2% aqueous osmium tetroxide [20].

### The expression of Diaph1, RAGE and beta-actin (ACTB) in SN

#### RNA isolation and reverse transcription

Total RNA was isolated from a whole, unilateral SN (n = 4, [19]) with the use of TRI Reagent^®^ (Sigma Aldrich, USA), according to the manufacturer’s protocol. Reverse transcription was conducted using the QuantiNova Reverse Transcription Kit (Qiagen, Valencia, CA, USA).

### The expression of Diaph1, gene encoding RAGE (AGER) and ACTB mRNAs

The expression of *Diaph1*, *AGER* and *ACTB* (Table 1) in SN harvested from each group (Fig. 1A) were analyzed in duplicates by LightCycler^®^ 480 Instrument II (Roche Diagnostics, Switzerland). The relative amplification of genes was calculated using the *ΔΔ*Ct method.

**Table 1:** TaqMan gene expression assays (ThermoFisher) used for *qPCR*

Gene symbol (official)	Assay ID	Catalog no.	Amplicon lenght (nt)	Dye
*DIAPH1*	Mm01258311_m1	4448892	64	6-carboxyfluorescein (6-FAM)
*ACTB*	Mm00607939_s1	4453320	115	
*AGER*	Mm01134790_g1	4351368	65	
*18S rRNA*	Mm03928990_g1	4453320	61	

### Localization and immunoreactivity of Diaph1, RAGE and ACTB proteins

The presence of Diaph1, RAGE and ACTB in SN sections was determined using a two-day procedure for semi-quantitative immunohistochemical (IHC) staining [1] (Table 3). Eight micron tissue samples were air dried and incubated with primary antibodies (Table 3) diluted in 0.1% BSA, overnight. To visualize the immunoreactivity, slides were incubated with 3,3 diaminobenzidine tetrahydrochloride (DAB, Sigma-Aldrich, USA) as well as with hematoxylin (Sigma-Aldrich, USA).

**Table 2:** Primers used for validation of NGS results

Official gene symbol		Sequences	NCBI reference sequence	Amplicon lenght (nt)	References
Differentially expressed genes					
*HAO1*		F: AGAGTCAGCATGCCAATATG	NM_010403.2	155	-
		R: GCTTCTGCCACTTCTTCTATT			
*NET1*		F: ACACCCACCAAGAGAAGA	NM_019671.3	139	-
		R: CCTCAATCAAGTCCTGTTCC			
*RHOJ*		F: TGTGTTTCTCATCTGCTTCTC	NM_023275.2	128	-
		R: GATCAATCTGGGTTCCGATG			
*TXNIP*		F: CAGTCAGAGGCAATCACATTA	NM_023719.2	131	-
		R: GTAGATCAGCAAGGAGTATTCG			
*CTSE*		F: CATATACCCTCAACCCAACTG	NM_007799.3	146	-
		R: GTAGAACTGTCGGATGAAGAC			
Reference genes					
*18S rRNA*		F: GGGAGCCTGAGAAACGGC	NR_003278.3	68	[26]
		R: GGGTCGGGAGTGGGTAATTT			
*IPO8*		F:CTATGCTCTCGTTCAGTATGC	NM_001081113.1	173	[21]
		R: GAGCCCACTTCTTACACTTC			

### The content of Diaph1, profilin 1 (PFN1), N(epsilon)-(carboxymethyl)lysine-AGEs (CML–AGEs), high mobility group box 1 (HMGB1), S100 calcium binding protein B (S100B), S100 calcium binding protein A6 (S100A6), superoxide dismutase type 1 (SOD1), ACTB and RAGE proteins in SN

#### Protein extraction

Whole SN (n = 6 per group; [19]) was homogenized with isolation buffers (Eurx^®^, Poland) in MagNA Lyser (Roche Diagnostics, Switzerland) according to the manufacturer’s protocol (Universal DNA/RNA/Protein Purification Kit, Eurx^®^). The protein concentration was determined using Direct Detect^®^ Infrared Spectrometer for Total Protein Quantitation (Merck Millipore, Darmstadt, Germany).

### Western blot analysis

Proteins were separated on gels and transferred onto nitrocellulose membrane using semi-dry system. Subsequently, the membrane was incubated in Blocking Buffer (Bio-Rad) and primary antibody solutions (Table 3). Images were quantified densitometrically with Image Lab v6.0.1 software.

### Co-localization of Diaph1 and ACTB as well as ACTB and PFN1 in SN

To investigate the co-expression and co-localization between Diaph1 and ACTB as well as ACTB and PFN1 in mouse SN, samples were collected six months after induction of diabetes (Fig. 1A). Immunofluorescence staining was performed according to standard laboratory protocols [3–6].

### Next-generation sequencing procedure

Total RNA was extracted from lumbar SC neuromere (n = 5 per group; [19]) harvested from 32-weeks-old mice. Subsequently, RNA integrity (RIN), quality and quantity were evaluated with microcapillary electrophoresis (2100 Bioanalyzer, Agilent Technologies, Santa Clara, CA, USA). Only samples with RIN above 8 were used for further analysis. The sequencing reactions were performed on the NovaSeq 6000 platform (Illumina^®^, USA) to generate 2 × 150 bp paired-end reads and 40 million readings per sample. Sequencing data was converted into raw data for the in silico analysis (Supplementary Fig. 1).

### Functional analysis of differentially expressed genes (DEGs)

The list of DEGs was uploaded to DAVID 6.8 to identify enriched biological themes, Gene Ontology (GO) terms and visualize KEGG pathways [22–24]. Further, the list of selected genes among DEGs in lumbar SC of diabetic mice with *AGER, Diaph1* and *ACTB* was uploaded to the GeneMANIA Prediction Server [25].

### The expression of hydroxyacid oxidase 1 (HAO1), neuroepithelial cell transforming 1 (NET1), ras homolog family member J (RHOJ), thioredoxin interacting protein (TXNIP), cathepsin E (CTSE) mRNAs in lumbar SC neuromere harvested in six months of diabetes

The expression of selected genes (Table 2) in lumbar SC harvested from diabetic mice (Fig. 1) performed by quantitative PCR (qPCR).

**Table 3:** Characteristics of antibodies used in experiments

Primary antibody						
Protein	Type		Species	Dilution	Usage	Manufacturer and catalog no.
DIAPH1	polyclonal		rabbit	1:100	IHC	Abcam, ab11173
	polyclonal		rabbit	1: 1 000	WB	Abcam, ab129167
	polyclonal		rabbit	1:50	IF	ThermoFisher, PA5-96420
ACTB	polyclonal		rabbit	1:200	IHC	Synaptic Systems, 251003
	monoclonal		mouse	1: 50 000	WB	Abcam, ab6276
	polyclonal		chicken	1:500	IF	Synaptic System, 251006
RAGE	polyclonal		rabbit	1:50	IHC	Abcam, ab37647
	polyclonal		rabbit	1:5 000	WB	Abcam, ab37647
PFN1	monoclonal		rabbit	1: 1 000	WB	Abcam, ab232020
	monoclonal		rabbit	1:100	IF	Abcam, ab232020
CML	polyclonal		rabbit	1: 5 000	WB	Abcam, ab27684
HMGB1	polyclonal		rabbit	1: 1 000	WB	Abcam, ab18256
S100B	monoclonal		rabbit	1: 1 000	WB	Abcam, ab52642
S100A6	monoclonal		rabbit	1: 1 000	WB	Abcam, ab134149
SOD1	polyclonal		rabbit	1: 5 000	WB	Abcam, ab13498

Secondary antibody						
Reagent	Host	Species reactivity	Dilution	Usage	Manufacturer and catalog no	
StarBright Blue 700 IgG	goat	rabbit	1: 10 000	WB	BioRad, 12004162	
StarBright Blue 800 IgG	goat	mouse	1: 10 000	WB	BioRad, 12004158	
IgG (H + L), Alexa Fluor Plus 594	goat	rabbit	1: 2 000	IF	ThermoFisher, A11039	
IgY (H + L), Alexa Fluor 488	goat	chicken	1: 2 000	IF	ThermoFisher, A-32740	

Explanation of abbreviations: *WB* Western blot analysis, *IF* immunofluorescence staining, *IHC* immunohistochemistry staining

### The comparison of SC and SN transcriptomes in diabetes

The comparison of DEGs in SC of T1D with the DEGs in T1D and T2D SN [27, 28] obtained from Gene Expression Omnibus (GEO) database (http://www.ncbi.nlm.nih.gov.geo/) was performed by creating a Venn diagram [29].

### Statistical analyses

Analyses with *P* values ≤ 0.05 were considered statistically significant. All data were presented as mean ± *SEM*. Before selecting the appropriate statistical test, we have performed the normality and lognormality test, *i.e. Shapiro–Wilk* test. Moreover, all datasets were tested for presence of outliers by using the Grubbs’ test (α = 0.05). Statistical analyses and graphs were performed using GraphPad Prism 9.1.0. (CA, USA).

## Results

### Induction of diabetes and loss of weight in mice

Blood glucose was increased already by over two folds at two months post-STZ injections in the experimental group and remained elevated throughout the study as to control groups (*P* ≤ 0.001, *P* ≤ 0.0001, respectively, Fig. 1B). No differences in weight and blood glucose level between control groups of mice were observed (*P* ≥ 0.05, Fig. 1B, C), however following the progression of the disease, steady decline in body mass was noted in diabetic groups (*P* ≤ 0.0001 in all cases, Fig. 1C).

### Effect on SN conduction velocity

We found that in T1D animal group, NCV was reduced both in MNCV and SNCV (Fig. 1D, E). The SNCV was lower at all time points in diabetic groups as compared to controls, however statistically significant reduction was noted first at 4 month and subsequently at 6 month of diabetes (*P* ≤ 0.01, *P* ≤ 0.05, respectively, Fig. 1D). In the control group no significant alteration of SNCV was observed during the entire period (*P* ≥ 0.05, Fig. 1D). MNCV was significantly decreased as early as two months when compared to controls and declined throughout the study in diabetic groups as compared to controls (*P* ≤ 0.05, *P* ≤ 0.05, *P* ≤ 0.01, respectively, Fig. 1E). Again, we did not observe a decline in MNCV during the examined period in the control group (*P* ≥ 0.05, Fig. 1D, E).

### SN morphometry in T1D murine model

**Fig. 2:**
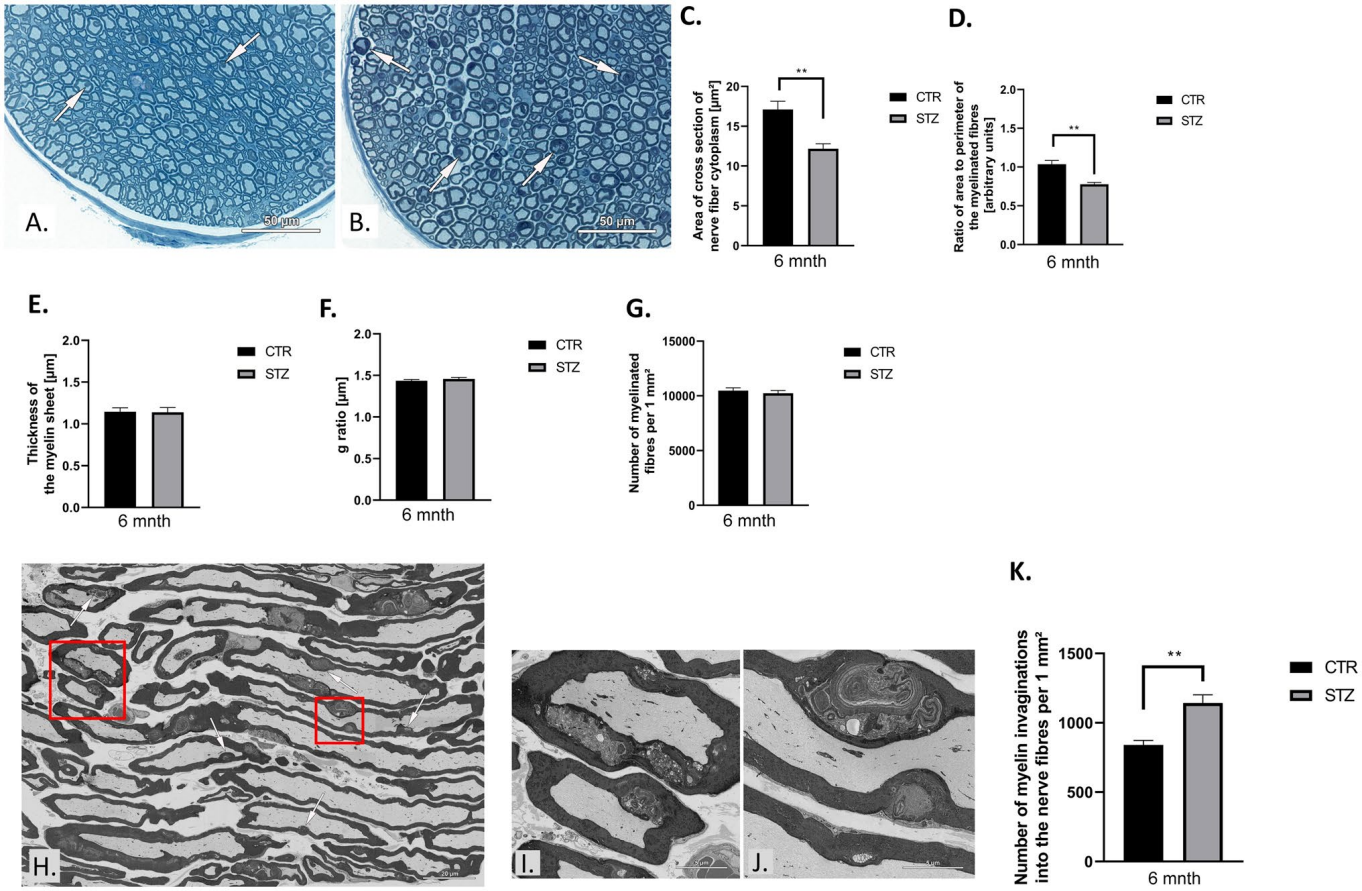
Type 1 diabetes (T1D) leads to morphometric, morphological and ultrastructural alternations in mouse peripheral nerves. **A**,** B** Toluidine blue-stained semithin sections used for morphometrical and morphological analyses. Representative parts of the transverse sections through the nerves of control mouse (**A**) and (**B**) STZ-treated mouse. Arrows show myelin invaginations into nerve fibers. Digital scans of semithin sections were used for measurements of cross section perimeter of nerve fiber and the thickness of the myelin sheet, for counting of the myelinated fiber per area unit. The right upper part of the semithin section with the area of 25 000 µm^2^ was chosen for analysis and all myelinated fibers located inside this area were measured. No less than 200 nerve fibers were measured per animal. Measurements were made using CaseViewer 2.1 (3DHistech Ltd, Budapest, Hungary). Scale bar = 50 µm. **C** T1D alters the area of cross section of nerve fiber cytoplasm as well as **D** the ratio of area to perimeter of the myelinated fibers. The T1D in not affected on **E** the thickness of myelin, **F** g ratio and **G** number of myelinated fibers per 1 mm2. **H** Myelin structure of sciatic nerve per longitudinal section area. The areas marked with red rectangles in **I-J** after zooming in. Scale bar = 5 µm. **K** Myelin structure of diabetic nerve fibers shows alternations in frequency of myelin invaginations. The whole sections were imaged using a backscatter detector in SEM Gemini 450 at 1.5 kV controlled by Atlas 5 software (Carl Zeiss, Oberkochen Germany). The digital ultrastructural images of longitudinal sections obtained in SEM were used for the semiquantitative analysis of myelin structure alternations, which was performed in the ATLAS Browser-Based Viewer 3.0. The analysis of semithin sections was performed with *Student's t*-test or *U Mann–Whitney* test. Data are expressed as means ± SEM; * *P* ≤ 0.05, ** *P* ≤ 0.01; n = 5 mice in each group. Abbreviation: CTR – control, STZ – diabetes, mnth – month

Toluidine blue stained semithin sections of diabetic SN showed no differences in the appearance of epineurium, perineurium and endoneurium (Fig. 2A, B). The analysis showed that the cytoplasm area of nerve fiber transverse section as well as the ratio of area to perimeter of these sections were decreased at sixth month of the experiment in the diabetic group (*P* ≤ 0.01 in both cases; Fig. 2C, D). Diabetes did not affect the myelin sheet thickness g-ratio as well as the number of myelinated nerve fibers per 1 mm^2^ of the nerve section (*P* ≥ 0.05, Fig. 2E−G, respectively).

The occurrence of myelin infoldings (Fig. 2H–K) was significantly higher in STZ-treated mice (*P* ≤ 0.01; Fig. 2K). These alterations locally affected diameter of the nerve fibers and the shape of their sections. Their occurrence was not usually close to the Schmidt-Lanterman’s incisures and the Ranvier’s nodes (Fig. 2H–K).

### The effect of T1D on the expression of Diaph1, RAGE, ACTB in mouse SN

**Fig. 3:**
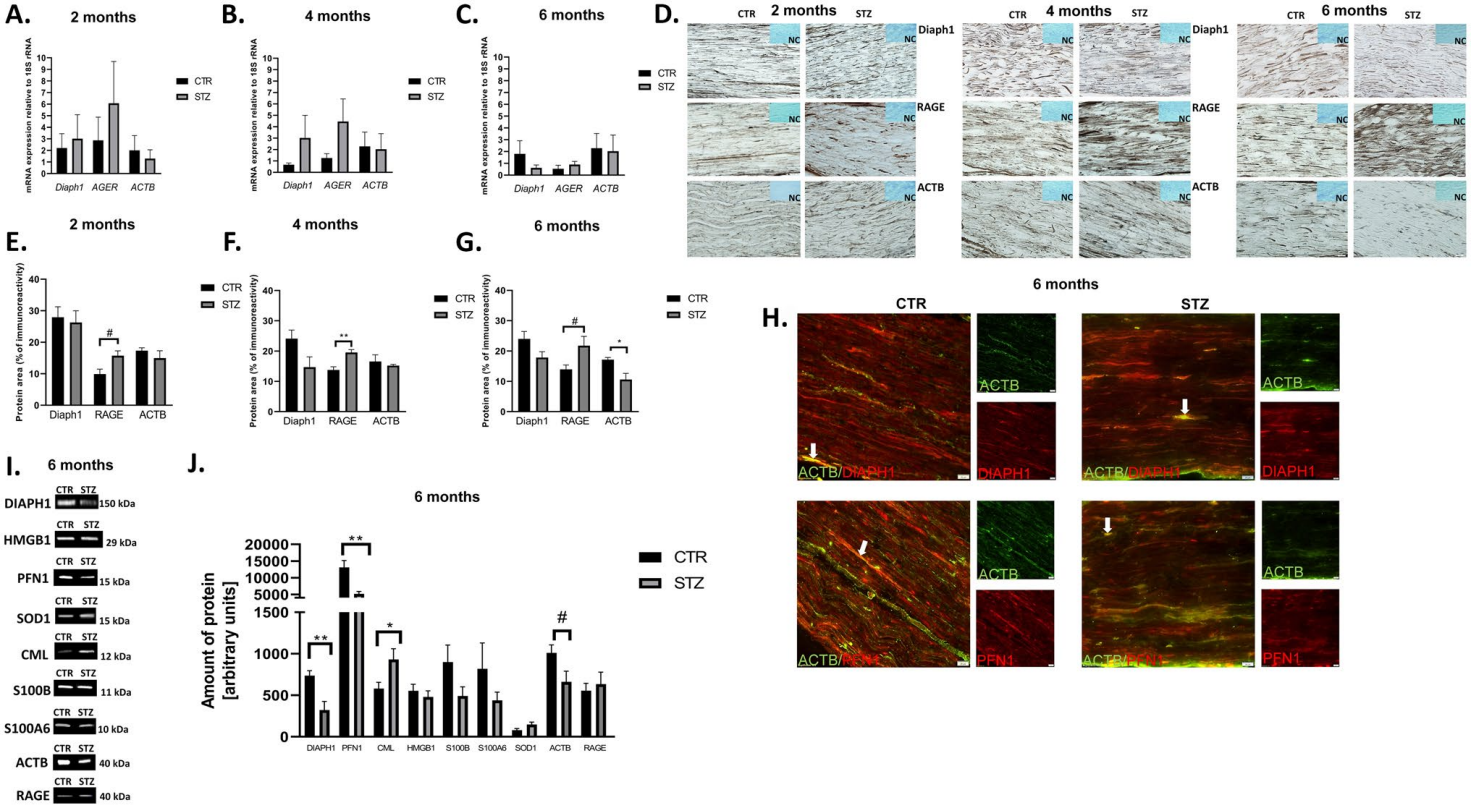
The effect of T1D on the expression of Diaph1, RAGE, ACTB in mouse SN. **A B C** The expression of *Diaph1, AGER* (gene encoding RAGE) and *ACTB* mRNAs in SN under hypoglycemic milieu *vs.* control group in two (**A**), four (**B**) and six (**C**) months of the experiment, respectively. Data are expressed as means ± SEM; n = 4 mice in each group. **D** Immunostaining of Diaph1, RAGE and ACTB under hyperglycemic conditions in mice SN. Negative control (NC) showed no immunostaining. Brown color demonstrated immunoreactive area of stain. Blue color demonstrated hematoxylin staining. Images were taken under 40× objective with 0.75 numerical aperture (40× /0.75). Scale bar = 100 µm. **E F G** The number of Diaph1, RAGE, ACTB fibers expression/immunoreactivity in diabetic SN as compared to control tissue in two (**E**), four (**F**) and six (**G**) months of the experiment, respectively. Data are expressed as means ± SEM; * *P* ≤ 0.05, ** *P* ≤ 0.01; # 0.05 ≤ *P* ≤ 0.056; n = 4 mice in each group. **I** Representative blots for Diaph1, PFN1, CML-AGE, HMGB1, S100B, S100A6, SOD1, ACTB and RAGE proteins of control (CTR) and after six months of T1D (STZ; see also supplementary files). Abbreviation: kDa – kilodalton. **J** The effect of T1D on the relative amount of Diaph1, PFN1, CML-AGE, HMGB1, S100B, S100A6, SOD1, ACTB and RAGE proteins in SN mice. Data are expressed as means ± SEM; * *P* ≤ 0.05, ** *P* ≤ 0.01; n = 6 CTR or 7 STZ. **H** Co-localization (arrows, yellow staining) of Diaph1-ACTB and ACTB-PFN1 in mice SN of T1D. Images were taken under 40× objective with 0.6 numerical aperture (40× /0.6). Scale bar = 20 µm. Abbreviation: CTR – control, STZ – diabetes, # - *P *= 0.07

The expression of Diaph1, *AGER* (gene coding RAGE) and ACTB mRNAs did not reach statistical significance (Fig. 3A–C). IHC revealed that at all examined time points Diaph1, RAGE and ACTB proteins were present in mouse SN (Fig. 3D). We observed an increasing trend of RAGE immunoreactivity area at two and six months of diabetes when compared with controls (*P* = 0.0551, *P* = 0.0527, respectively; Fig. 3E, G). Intensity of RAGE immunostaining at four months of diabetes was significantly elevated when compared to control group (*P* ≤ 0.01; Fig. 3F). Moreover, IHC staining revealed that intensity of ACTB immunostaining was decreased at all time points and reached statistical significance at sixth month of T1D comparing to controls (*P* ≥ 0.05, *P* ≥ 0.05, *P* ≤ 0.05; respectively, Fig. 3G).

### The effect of diabetes on Diaph1, PFN1, CML–AGEs, HMGB1, S100B, S100A6, SOD1, ACTB and RAGE expression in mouse SN

Our results indicated that Diaph1 was reduced in diabetic SN compared to controls (*P* ≤ 0.01, Fig. 3J), consistent with earlier studies. At six months of diabetes the amount of Diaph1 as well as PFN1 was decreased and the relative amount of CML-AGE was elevated when compared to control mice (*P* ≤ 0.01, *P* ≤ 0.01, *P* ≤ 0.05, respectively, Fig. 3J). We did not observe differences in the relative amount of HMGB1, S100B, S100A6 and SOD1 proteins in SN harvested from diabetic and control mice at six months after diabetes induction in our experiment (*P* ≥ 0.05, Fig. 3J). Intensity of ACTB expression at six months of diabetes showed a downward trend compared to the control group (*P* = 0.07; Fig. 3J). However, we observed a trend towards increased RAGE expression in STZ mice, consistent with CML-AGE (Fig. 3J). Our analysis confirmed the specificity of antibody and protein expression patterns in mice sciatic nerve (Fig. 3I). Finally using immunofluorescence, we observed co-localization of Diaph1-ACTB and ACTB-PFN1 in SN of T1D mice (Fig. 3H).

### Sequencing results

More than 18 million (M) reads in each sample were achieved (Supplementary Fig. 1). After the trimming, reads were mapped to the reference mouse genome vGRCm39.104 from the Ensemble database (Supplementary Fig. 2). Principal component analysis (PCA) showed a high transcriptome differentiation between diabetics and controls (Supplementary Fig. 3).

**Fig. 4:**
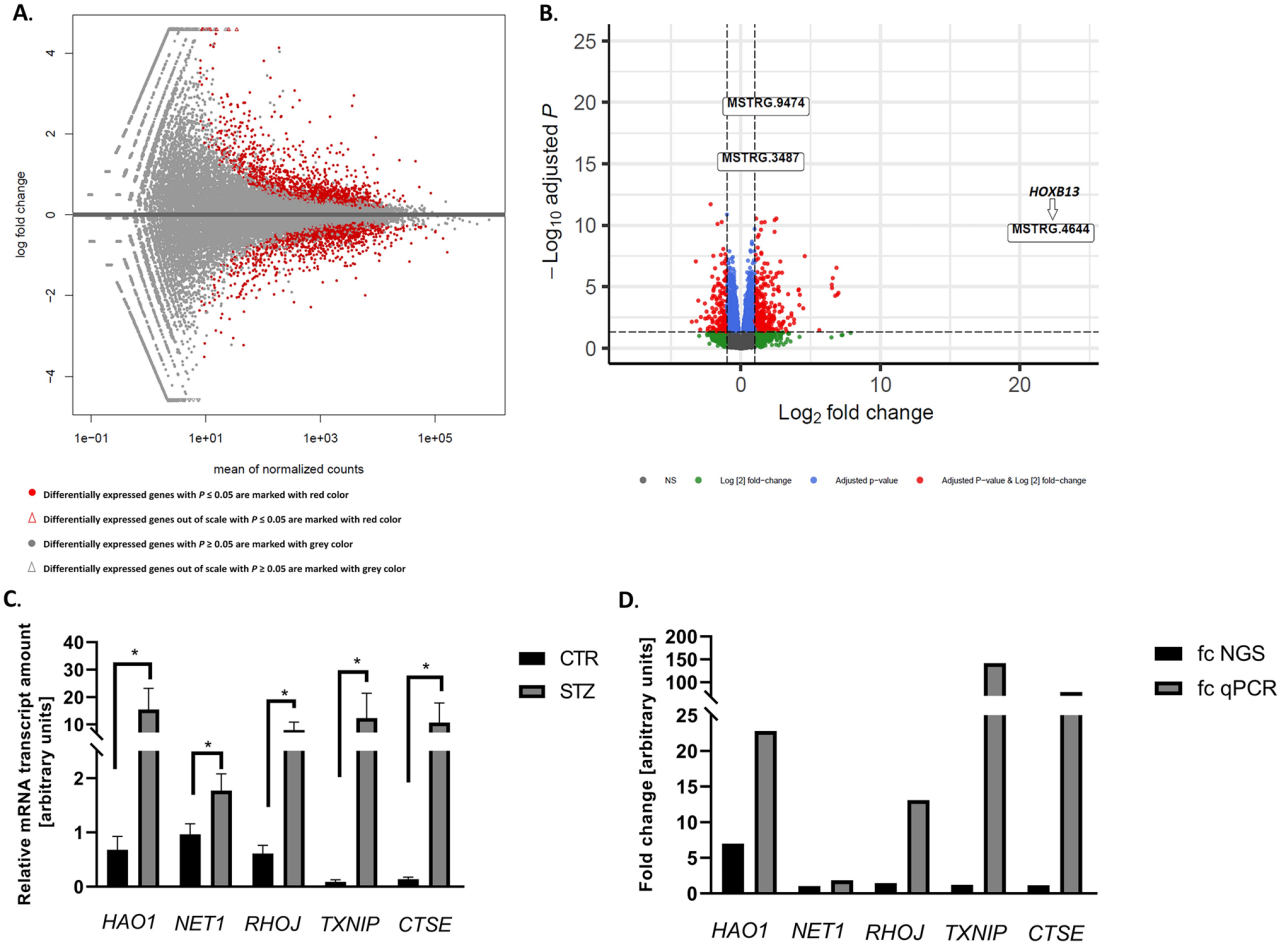
Overall statistics of RNA-seq data. **A** MA plot shows the logarithmic value of log fold change (fc; Y axis) and mean of normalized counts (X axis) for comparing STZ-treated and control libraries. **B** Volcano plot indicates logarithmic value of fold change (fc; X axis) and negative logarithmic adjusted P values (Y axis). On both plots DEGs with *P* ≤ 0.05 are marked red dots. The *HOXB13* is the most altered genes in diabetic spinal cord. NS – no significant. **C** The relative mRNA expression of genes with altered expression in mouse lumbar SC under hyperglycemic conditions. The amount of mRNA transcript are presented as a mean 2^−ΔΔCt^ values ± SEM. * *P* ≤ 0.05, ** *P* ≤ 0.01; n = 4 mice in each group. **D** Fold change (fc) of selected genes in lumbar SC harvested from T1D mice. The results were consistent throughout the study

We then analyzed the expression profiles of DEGs in the diabetic lumbar SC samples. Approximately 31 453 transcripts were identified in lumbar SC (Fig. 4A). Analysis revealed that among 31 453 transcripts, 538 genes revealed differential expression in studied samples (*P* ≤ 0.05; Fig. 4B, Supplementary Table 1). Among 538 (248 up- and 137 down-regulated) DEGs, 385 had known official gene symbol (Supplementary Table 1) and of those, 330 has known biological function as per DAVID database. The highest expression gene was homeobox B13 (*HOXB13*; Fig. 4B) and the most down-regulated gene was RIKEN cDNA 5033426O07 gene (*5033426O07Rik,* Supplementary Table 1). Further, of those 330 genes we found genes affiliated with long non-coding RNAs (lncRNAs), such as: small nucleolar RNA host gene 15 (*SNHG15*, up-regulated) and deleted in lymphocytic leukemia 2 (*DLEU2,* down-regulated). The expression of SPOC domain containing 1 (*SPOCD1)* piwi-interacting RNA (pi-RNA) was up-regulated in lumbar SC of mice with T1D (Supplementary Table 1). We were able to validate expression of many of the prominent DEGs, *i.e.* hydroxyacid oxidase 1 (*HAO1*)*,* neuroepithelial cell transforming 1 (*NET1*)*,* ras homolog family member J (*RHOJ*)*,* thioredoxin interacting protein (*TXNIP*), cathepsin E *(CTSE)* with RNA-Seq results (Fig. 4C). Results of qPCR confirmed the veracity of the RNA-Seq data (Fig. 4D).

### Functional gene analysis

**Fig. 5:**
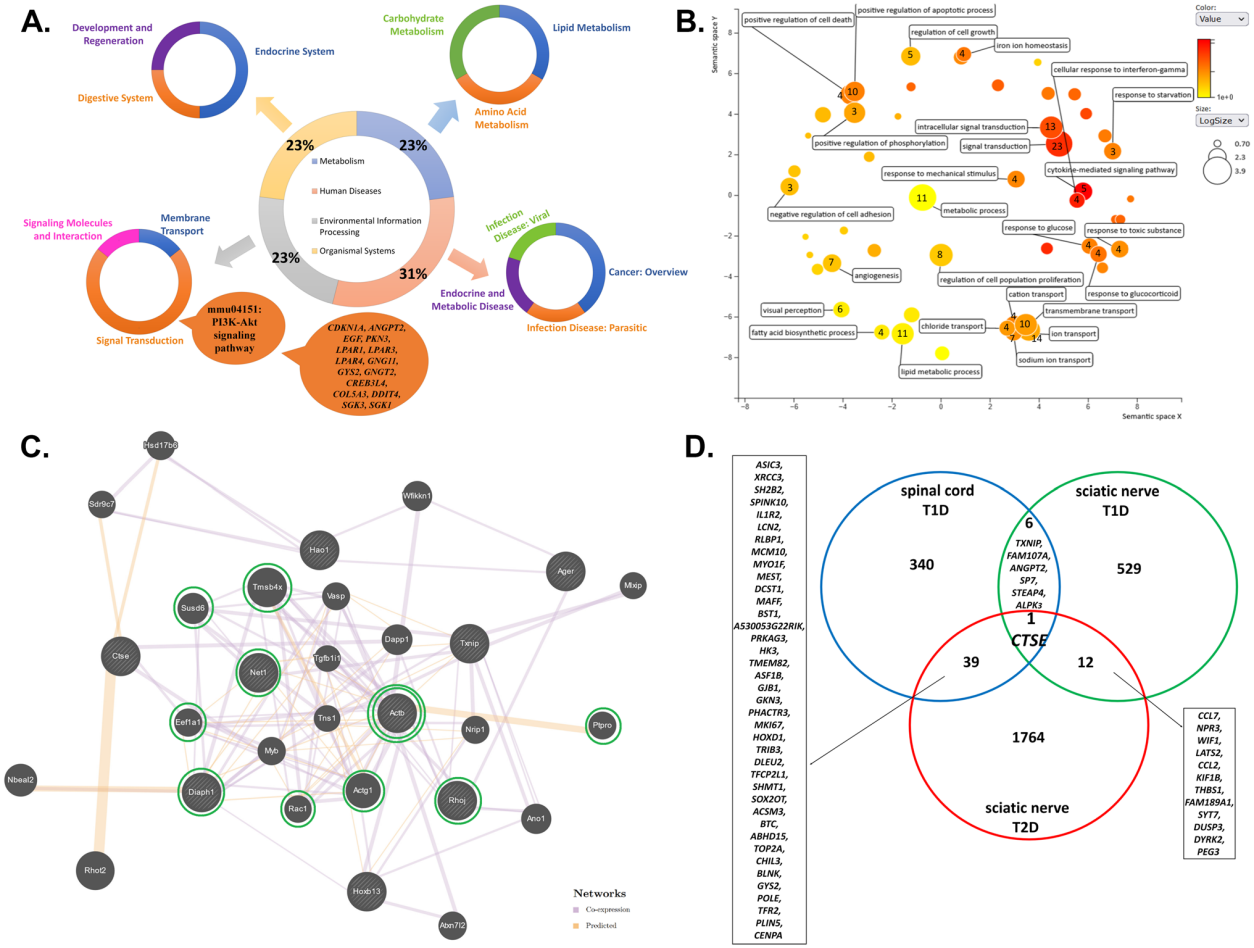
Functional DEGs analysis. **A** The diagram of expression data for DEGs with known biological functions and their participation in signaling pathways from KEGG database. The analysis of signaling pathways demonstrated four major categories, such as: environmental information processing, metabolism, human diseases, organismal systems (central pie chart), and 13 subcategories (lateral pie chart). The most enriched biological pathway in lumbar SC from STZ-treated mice was the PI3K-Akt signaling pathway (mmu04151). The cloud indicates genes involved in PI3K-Akt signaling pathway. **B** Functional analyses of DEGs by REVIGO gene ontology (GO) enrichment analysis [50]. The most representative GO terms for biological process (BP) were utilized for REVIGO functional annotation clustering that relies on semantic similarity measures. Bubble color corresponds the *P*-value obtained from GO enrichment analysis, whereas the bubble size corresponds to the GO term prevalence in the UniProt-GOA database for *Mus musculus*. Arabic numeral indicates the number of DEGs in SC. See also Supplementary Table 2 for details on the GO-BP terms. **C** Gene interaction network constructed with a use of GeneMANIA for selected set of genes, *i.e*. *HOXB13, HAO1, NET1, RHOJ, TXNIP, CTSE, AGER, DIAPH1, ACTB* (limited to *Mus musculus*). The selected genes are in a striped circle. The colors of the line suggest the type of interaction (see legend). In silico analysis indicated that *ACTB* gene has the most direct interactions (double green circle). Green circles indicate genes that have a direct interaction with *ACTB, i.e. NET1, TMSB4X, SUSD6, EEF1A1, DIAPH1, RAC1, ACTG1, RHOJ, PTPRO* genes. See also Supplementary Table 6 for details on the interaction network. **D** Venn diagram [29] representing DEGs in SC of T1D, SN of T1D and T2D [27, 28]. The analysis showed that the expression of a one, single gene, *i.e. CTSE* was altered for all tissue transcriptome profiles

The gene ontology (GO) enrichment analysis suggested that among DEGs, a total of 330 with known biological functions could be categorized under the hierarchical GO terms (Supplementary Tables 2, 3 and 4). Briefly, within the biological process (BP) aspect, DEGs were enriched in 55 functions (Fig. 5B). In molecular function (MF) hierarchy, there were 17 significant GO terms; in case of cellular component (CC) hierarchy, DEGs were enriched to 10 GO terms (Supplementary Tables 2, 3 and 4). The further analysis of the 330 DEGs demonstrated four major categories, including environmental information processing, metabolism, human diseases, organismal systems and 13 subcategories (Fig. 5A) as well as 19 biological pathways (Supplementary Table 5). The most enriched biological pathway in lumbar SC neuromere from STZ-treated mice was PI3K-Akt signaling pathway (mmu04151, Fig. 5A, Supplementary Table 5). The DEGs in GO enriched categories of BP were mainly involved in signal transduction (GO:0007165), intracellular signal transduction (GO:0035556), ion transport (GO:0006811), metabolic process (GO:0008152), lipid metabolic process (GO:0006629), transmembrane transport (GO:0055085), positive regulation of apoptotic process (GO:0043065), regulation of cell proliferation (GO:0042127), sodium ion transport (GO:0006814) and angiogenesis (GO:0001525; Fig. 5B).

### Interaction network

The GeneMANIA analysis of selected genes, *i.e. HAO1, NET1, RHOJ*, *TXNIP, CTSE* (based on validation)*, DIAPH1, AGER* and *ACTB* (based on our previous studies and literature) as well as the highest expressed gene in diabetic spinal cord, *i.e. HOXB13*, revealed that all of them were connected in single network (Fig. 5C). It was constructed by 29 genes (20 related genes) and consisted of a total number of 122 interactions. Moreover, genes building a network were involved in the following functions: transepithelial transport (*ACTB,* actin gamma 1 (*ACTG1*)), actin polymerization or depolymerization (thymosin beta 4 x-linked (*TMSB4X*)*,* vasodilator stimulated phosphoprotein (*VASP*)*, DIAPH1,* rac family small GTPase 1 (*RAC1*)), structural constituent of synapse (*ACTB, ACTG1*), regulation of actin filament organization (*TMSB4X, VASP, ACTG1, RAC1*), cell-substrate junction organization (tensin 1 (*TNS1*)*, ACTG1, RAC1*), structural constituent of postsynapse (*ACTB, ACTG1*), ruffle (eukaryotic translation elongation factor 1 alpha 1 (*EEF1A1*)*, DIAPH1, RAC*, cellular response to glucose stimulus (*RAC1,* anoctamin 1 (*ANO1*)), protein localization to cell–cell junction (*ACTB, ACTG1*) and protein polymerization (*TMSB4X, VASP, DIAPH1, RAC1*). In silico analysis revealed that *ACTB* was a node of the network and the most common interaction between genes was co-expression (Supplementary Table 6). We found that *ACTB* gene has direct interactions, *i.e.* co-expression, with sushi domain containing 6 (S*USD6*)*, TMSB4X, EEF1A1, RHOJ, ACTG1, RAC1, NET1, DIAPH1* genes as well as predicted interaction with protein tyrosine phosphatase receptor type O (*PTPRO*) gene (Fig. 5C).

### Comparison of SC and SN transcriptomes in diabetes

Among all studied DEGs, 385 in SC, 548 in SN of T1D and 1816 in SN of T2D, the expression of only one gene, *i.e*. Cathepsin E (*CTSE*) was altered in all cases (Fig. 5D; [27, 28]). Both in the SC of T1D and SN of T1D or T2D seven and 40 genes were commonly altered, respectively (Fig. 5D).

## Discussion

Our results indicate that the progression of DPN is associated with alterations in NCV, morphometry and SN ultrastructure as well as the expression of molecules in RAGE-Diaph1 signaling pathway. Diaph1 has the ability to regulate actin polymerization and modification of actin cytoskeleton in nervous system [1, 2, 9, 30, 31]. The reduction in the amount of Diaph1 may lead to abnormal organization of actin cytoskeleton in diabetic SN [1, 2, 9, 30, 31]. In line with this reported association, our data revealed reduction of ACTB and PFN1 in SN after six months of T1D. PFN1 is a master regulator of cytoskeleton structure and may enhance F-actin elongation. Overexpression of PFN1 may however prevent the polymerization of ACTB [32]. PFN1 dysfunction may lead to changes in the structure of nuclear membrane, with the presence of frequent folds and invaginations [32]. Indeed we observed myelin invaginations into nerve fibers and a decline in SN myelinated axons in hyperglycemic environment. Moreover, we observed elevated presence of CML-AGEs in diabetic SN [3–9]. Evidence shows that excessive deposition of AGEs, reactive derivatives of methyl-glyoxal, is a hallmark of extensive glycation likely affecting cytoskeleton proteins and thus damage the actin cytoskeleton of nerve fibers in diabetic SN [33].

Overall, our data confirmed that T1D affects PNS. However, the soma of motor neuron for SN resides in lumbar SC, therefore we decided to look at molecular changes in lumbar SC in contrast to previous studies which focused solely on SN [9, 10].

Our sequencing analysis demonstrated that DEGs in lumbar SC were the most representative in BP GO term. The most enriched categories were those related to signal transduction, with PI3K-Akt signaling pathway (mmu04151) being the most enriched pathway. PI3K-Akt signaling pathway is engaged in multiple functions in cells, such as metabolism, cell survival, proliferation and angiogenesis in response to extracellular factors. It is also involved in the regulation of glucose level in cells and regeneration of PNS as well as nerves growth in CNS [34]. To date, countless previous studies have showed that the neurotrophins and nerve growth factor may active PI3K-Akt pathway in nervous system [34–37]. Hence, the PI3K-Akt signaling pathway plays a crucial role in mediating the survival as well as neurite outgrowth processes in PNS and CNS [34–37]. How aberrant function of this pathway may affect progression of DPN will be topic of future research and may provide target for intervention in DPN.

Our results suggest that the expression of *SNHG15* lncRNAs was up-regluated in SC of diabetic mice. *SNHG15* lncRNA alters the expression level of target proteins like TXNIP [38, 39]. Increase in expression of TXNIP was reported in the plasma of diabetic patients [40]. Dunn and co-workers [41] showed that elevated expression of TXNIP protein may trigger endothelial dysfunctions by inhibiting synthesis of vascular endothelial growth factor (VEGF). Our study demonstrates simultaneous overexpression of *SNHG15* as well as *TXNIP* in lumbar SC of T1D mice. We speculate that elevated expression of *SNHG15* may be compensatory to overexpression of *TXNIP* in lumbar SC in hyperglycemia. However, further studies are needed to elucidate *SNHG15* lncRNA effect on the *TXNIP* expression pattern in diabetes.

The GeneMANIA analysis revealed that genes involved in oxidative stress (*HAO1)*, RhoA signaling pathway (*NET1, RHOJ)*, endothelial dysfunctions (*TXNIP),* transcription process (*HOXB13),* inflammation (A*GER)* neuronal death signaling pathway (*CTSE),* axonal cytoskeleton*,* actin polymerization (*DIAPH1)* and cytoskeleton remodeling (*ACTB)* were connected in singular network [3–9, 41–45]. The analysis showed that *ACTB* has direct links with genes related to RhoA signaling pathway (*NET1*), formation of F-actin-rich structures (*RHOJ*), axonal cytoskeleton, actin polymerization (*DIAPH1, TMSB4X, EEF1A1, ACTG1, RAC1, ACTG1*), growth-suppressive and cell death (*SUSD6*), GTPase and actin bundling protein (*EEF1A1*), glucose uptake as well as activate the PI3K kinase (*RAC1*), cell adhesion as well as cell-surface mediated signaling (*ITGA6*), transports of proteins to the lysosomes (*CHAMP1A*), diabetic kidney disease (*PTPRO*) [46–49]. Overall, we observed that most of the aforementioned genes are involved in cytoskeletal reorganization as well as actin polymerization.

Interestingly, we identified a single gene *CTSE* as an intersection point of DEGs from T1D SC, T1D and T2D SN. It is pertinent to mention that while the expression of *CTSE* in SN of T2D as well as lumbar SC of T1D was elevated it was declined in SN harvested from T1D mice [27, 28]. The function of CTSE is not well understood, however it may play a role in amyloid polyneuropathy, neuropathy in experimental autoimmune encephalitis and neuroinflammation associated with Alzheimer’s disease [47–49]. Cathepsin E enzyme is involved in neuronal death signaling pathway [45]. Moreover, bioinformatic analysis of DEGs associated with SC injury revealed that *CTSE* gene was up-regulated [49]. Thus it is likely that *CTSE* may be a common molecule that is dysregulated in neurological disorders and may serve as a broad target for DPN and other neurodegenerative diseases warranting further investigations. Our studies revealed that molecular perturbations in SC may also be involved in the progression of DPN.

Overall, our results showed that alterations of diabetic SC transcriptome may act synergistically with changes in RAGE-Diaph1 signaling pathway in diabetic SN. However, in both SC and SN in hyperglycemia, the expression of one, single gene, *i.e. CTSE* involved in neuronal death signaling pathway was affected. Further, our results revealed that PI3K-Akt axis may be involved in progression of DPN.

## Supplementary Information

Below is the link to the electronic supplementary material.

**Supplementary Fig. 1.** The diagram of raw data analysis – *in silico *analysis. The md5sum is designed to verify data integrity using Message Digest Algorithm 5 (MD5). Consequently, regions of row reads were trimmed with use of Trimmomatic v0.38 program. Next, mapping was performed using STAR tool. For mapping the *Mus musculus* GRCm39 was used as the reference genome with annotation version GRCm39.104 downloaded from Ensembl database (https://www.ensembl.org/index.html). Statistical analyses of differentially expressed genes (DEGs) for protein-coding RNA affected by long term hyperglycemia was performed using Ballgown, dedicated Bioconductor v3.14 (https://bioconductor.org/packages/release/bioc/html/ballgown.html) package prepared for the R environment v4.1.2, with the following operating parameters: *P*-value < 0.05 and |log_2_FC| ≥ 0.5 (TIF 2504 KB)

**Supplementary Fig. 2.** Summary of the results of RNA sequencing, preprocessing, and mapping of reads to the mice reference genome (*Mus musculus* GRCm39.104). (TIF 1032 KB)

**Supplementary Fig. 3.** Principle component analysis (PCA). The PCA revealed the high level of differentiation between control and STZ-treated samples. Principal component analysis (PCA) and Euclidean distances between samples analysis were performed using ggplot2 library v3.3.5 (https://www.rdocumentation.org/packages/ggplot2/versions/3.3.5) and self-developed R script, to assess the overall similarity between transcriptomic profiles of RNA samples derived from diabetic and non-diabetic mice. Wheel – control, triangle – diabetes/STZ-treated mice. (TIF 510 KB)

**Supplementary Table 1**. Altered transcripts in lumbar spinal cord (SC) neuromere under hyperglycemic condition. (DOCX 120 KB)

**Supplementary Table** **2.** The table with GO-BP terms(DOCX 25 KB)

**Supplementary Table** **3.** The table with GO-MF terms (DOCX 17 KB)

**Supplementary Table** **4.** The table with GO-CC terms (DOCX 18 KB)

**Supplementary Table 5.** Functional enrichment analysis of DEGs in diabetic spinal cord (SC) of mice by KEGG database (DOCX 19 KB)

**Supplementary Table 6.** The table of interaction network in mouse diabetic spinal cord (SC) by GeneMANIA analysis (DOCX 20 KB)

**Supplementary file 1: **Western blot raw data (DOCX 3728 KB)

**Supplementary file 2: **Detailed methodology (DOCX 24 KB)

## Data Availability

The original results presented in the study are included in the article, further inquiries can be directed to the corresponding author. RNA-seq data have been deposited in the ArrayExpress database at EMBL-EBI (www.ebi.ac.uk/arrayexpress) under accession number E-MTAB 12252.

## Abbreviations

ACTG1: Actin gamma 1
ACTβ/ACTB: Beta (β) isoform of actin/ beta actin
AGEs: Advanced glycation end products
ALS: Amyotrophic lateral sclerosis
ANO1: Anoctamin 1
BP: Biological process
CC: Cellular component
CMAPs: Compound muscle action potentials
CML: Carboxymethyl-lysine
CNS: Central nervous system
CTSE: Cathepsin E
DAVID: Database for Annotation, Visualization and Integrated Discovery
DEGs: Differentially expressed genes
DIAPH1: Diaphanous related formin 1
DPN: Diabetic peripheral neuropathy
EEF1A1: Eukaryotic translation elongation factor 1 alpha 1
F-actin: Actin filaments
GEO: Gene Expression Omnibus database
GO: Gene Ontology
HAO1: Hydroxyacid oxidase 1
HMGB1: High-mobility group box 1 protein
HOXB13: Homeobox B13 actin gamma 1
IPO8: Importin 8
lncRNAs: Long non-coding RNAs
MF: Molecular function
MNCV: Motor nerve conduction velocity
NCV: Nerve conduction velocity
NET1: Neuroepithelial cell transforming 1
NGS: Next-generation sequencing
PFN1: Profilin 1
pi-RNA: Piwi-interacting RNA
PTPRO: Protein tyrosine phosphatase receptor type O
PNS: Peripheral nervous system
qPCR: Quantitative PCR
RAC1: Rac family small GTPase 1
RAGE: Receptor for advanced glycation end-products
RHOJ: Ras homolog family member J
RT: Room temperature
SC: Spinal cord
SEM: Scanning electron microscopy
SN: Sciatic nerve
SNCV: Sensory nerve conduction velocity
SNHG15: Small nucleolar RNA host gene 15
SOD1: Superoxide dismutase 1
SPOCD1: SPOC domain containing 1
STZ: Streptozotocin
SUSD6: Sushi domain containing 6
S100B: S100 calcium-binding protein B
S100A6: S100 calcium binding protein A6
T1D: Type 1 diabetes
T2D: Type 2 diabetes
TXNIP: Thioredoxin interacting protein
18S rRNA: 18S ribosomal RNA
TMSB4X: Thymosin beta 4 x-linked
TNS1: Tensin 1
VASP: Vasodilator stimulated phosphoprotein
