# Supplementary figures and images for: Novel insights into the nervous system affected by prolonged hyperglycemia
Source: J Mol Med (Berl). 2023 Jul 18;101(8):1015–28. doi: 10.1007/s00109-023-02347-y (PMC10400689; doi:10.1007/s00109-023-02347-y)

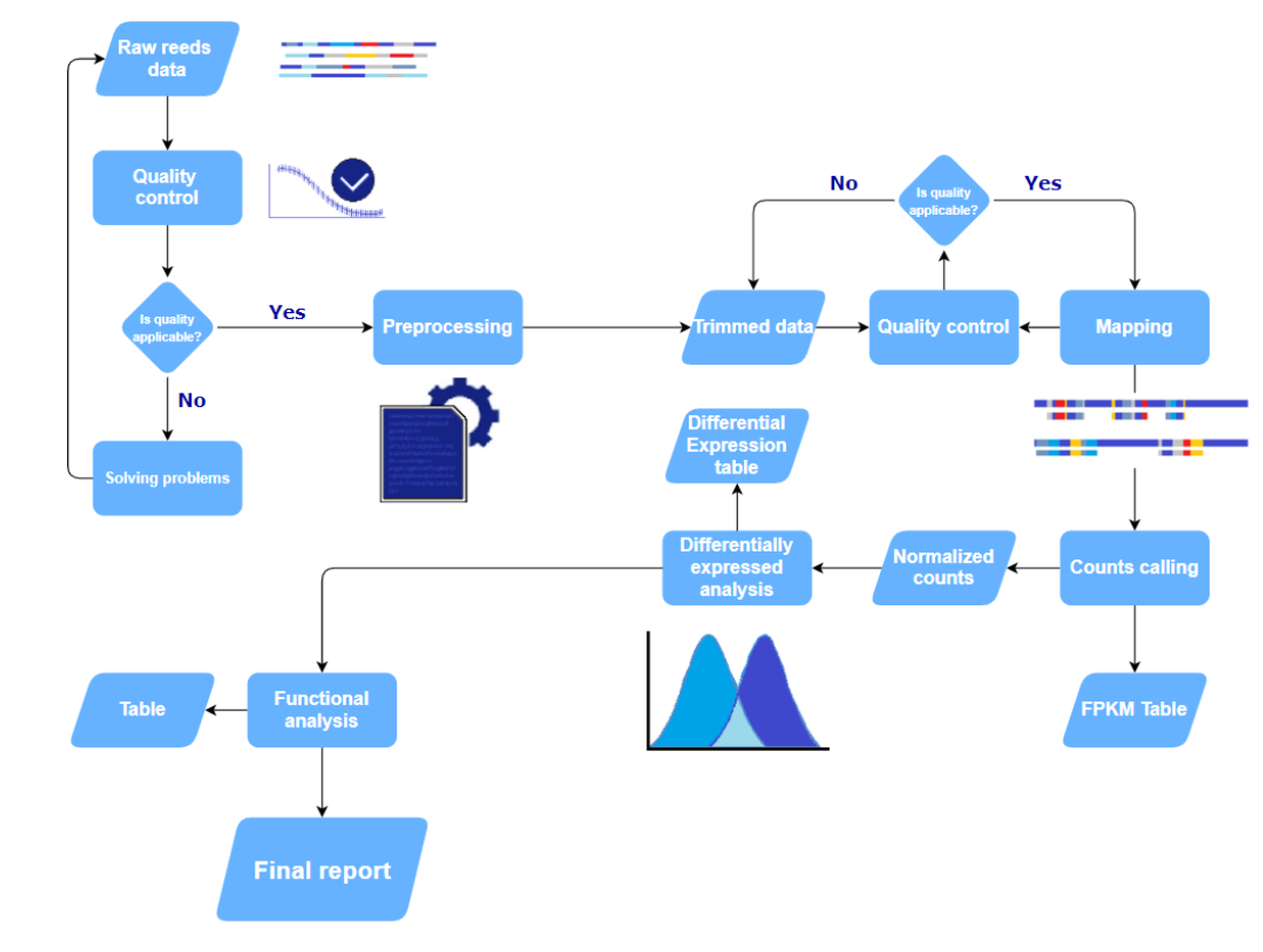

Supplement: Supplementary file 1 — Supplementary Fig. 1. The diagram of raw data analysis – in silico analysis. The md5sum is designed to verify data integrity using Message Digest Algorithm 5 (MD5). Consequently, regions of row reads were trimmed with use of Trimmomatic v0.38 program. Next, mapping was performed using STAR tool. For mapping the Mus musculus GRCm39 was used as the reference genome with annotation version GRCm39.104 downloaded from Ensembl database (https://www.ensembl.org/index.html). Statistical analyses of differentially expressed genes (DEGs) for protein-coding RNA affected by long term hyperglycemia was performed using Ballgown, dedicated Bioconductor v3.14 (https://bioconductor.org/packages/release/bioc/html/ballgown.html) package prepared for the R environment v4.1.2, with the following operating parameters: P-value < 0.05 and |log2FC| ≥ 0.5 (TIF 2504 KB) [file 109_2023_2347_MOESM1_ESM.tif]

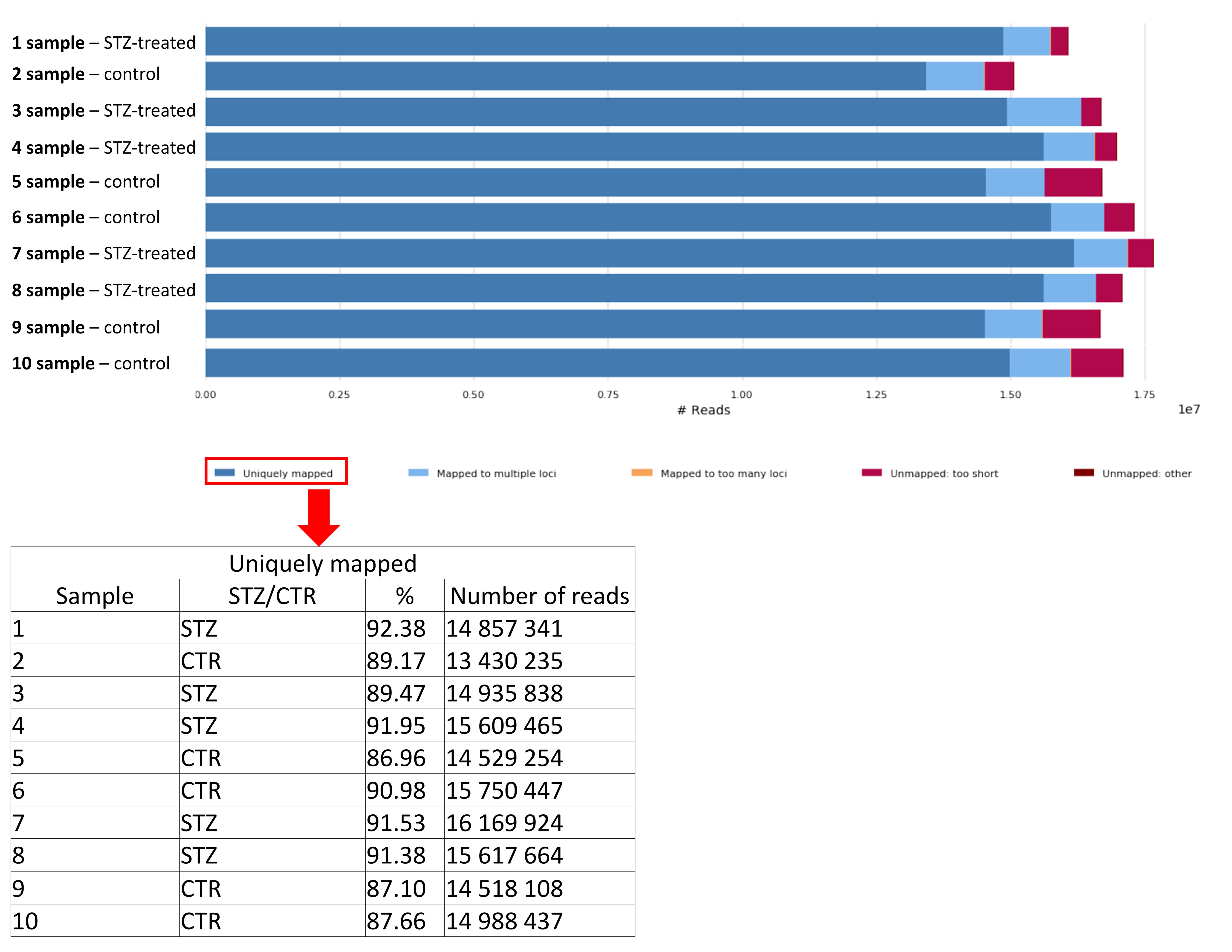

Supplement: Supplementary file 2 — Supplementary Fig. 2. Summary of the results of RNA sequencing, preprocessing, and mapping of reads to the mice reference genome (Mus musculus GRCm39.104). (TIF 1032 KB) [file 109_2023_2347_MOESM2_ESM.tif]

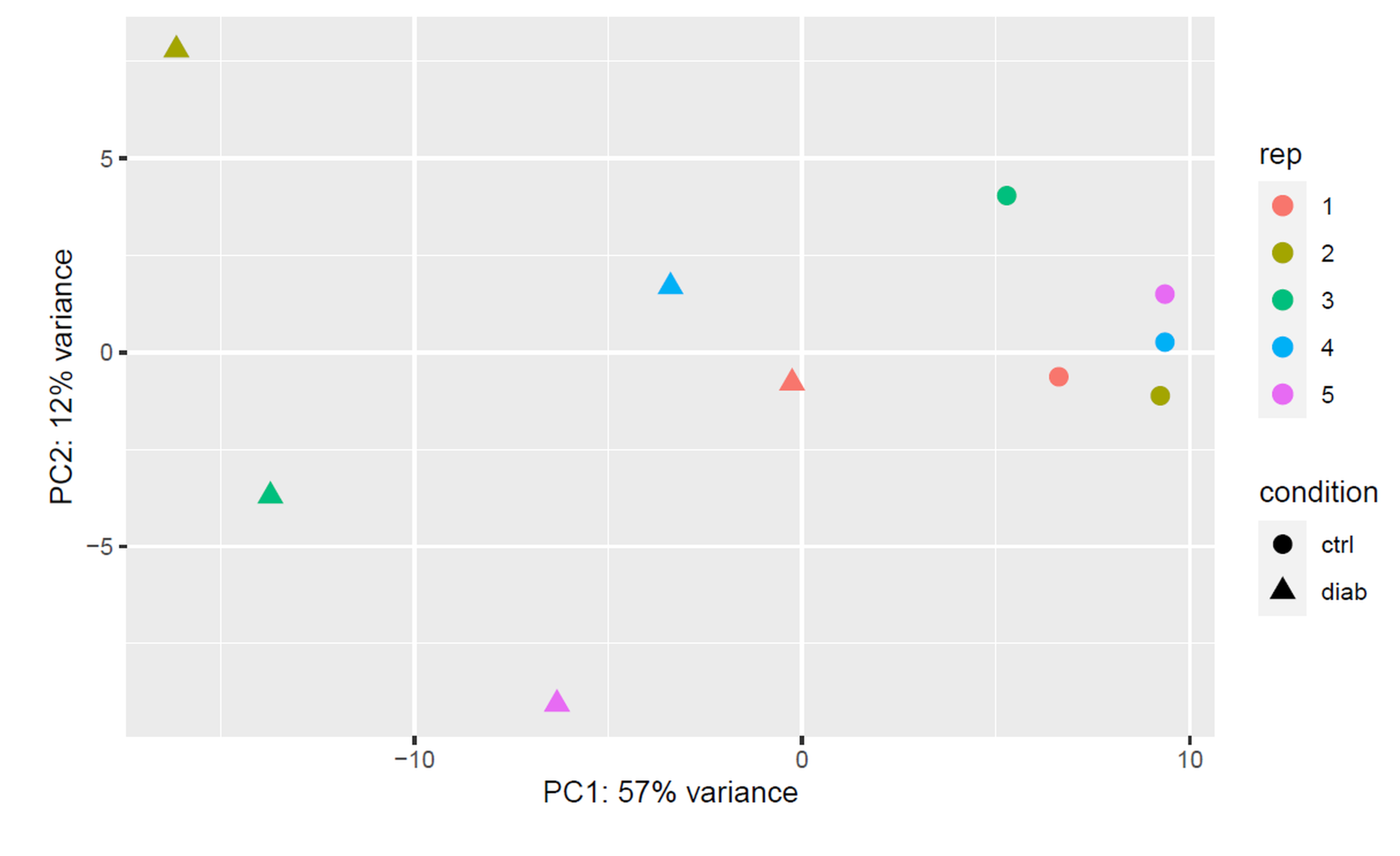

Supplement: Supplementary file 3 — Supplementary Fig. 3. Principle component analysis (PCA). The PCA revealed the high level of differentiation between control and STZ-treated samples. Principal component analysis (PCA) and Euclidean distances between samples analysis were performed using ggplot2 library v3.3.5 (https://www.rdocumentation.org/packages/ggplot2/versions/3.3.5) and self-developed R script, to assess the overall similarity between transcriptomic profiles of RNA samples derived from diabetic and non-diabetic mice. Wheel – control, triangle – diabetes/STZ-treated mice. (TIF 510 KB) [file 109_2023_2347_MOESM3_ESM.tif]
