# Supplementary material for: Novel insights into the nervous system affected by prolonged hyperglycemia
Source: J Mol Med (Berl). 2023 Jul 18;101(8):1015–28. doi: 10.1007/s00109-023-02347-y (PMC10400689; doi:10.1007/s00109-023-02347-y)
Supplement: Supplementary file 4 — Supplementary Table 1. Altered transcripts in lumbar spinal cord (SC) neuromere under hyperglycemic condition. (DOCX 120 KB) [file 109_2023_2347_MOESM4_ESM.docx]

| **Supplementary Table 1**. Altered transcripts in lumbar spinal cord (SC) neuromere under hyperglycemic condition | | | | | | | | |
| --- | --- | --- | --- | --- | --- | --- | --- | --- |
| Sequence ID | baseMean | log2FoldChange | lfcSE | stat | p-value | padj | Official gene name |  |
| **MSTRG.4644** | **34.6161811314099** | **22.5145853597238** | **3.02714823837077** | **7.43755626973899** | **1.02564900018245e-13** | **1.64402987654245e-10** | **Hoxb13** |  |
| **MSTRG.15199** | **12.0474831370988** | **6.99549013722077** | **1.38033959157079** | **5.06794862651161** | **4.02125895730859e-07** | **3.13545900296704e-05** | **Hao1** |  |
| **ENSMUSG00000024903** | **11.4069757695511** | **6.91835005866795** | **1.38928338859839** | **4.97979758157742** | **6.36508113641851e-07** | **4.32623094201449e-05** | **Lao1** |  |
| **MSTRG.23504** | **10.9173460116425** | **6.85330546466321** | **1.11672121131433** | **6.13698870875494** | **8.41003608763269e-10** | **2.88869721688598e-07** | **Gm44969** |  |
| **MSTRG.26282** | **10.2320768708614** | **6.76088697025091** | **1.3723609009968** | **4.92646428890549** | **8.37309066283606e-07** | **5.42277437372564e-05** | **Gm26759** |  |
| **MSTRG.6371** | **9.06478351762123** | **6.58502491920605** | **1.15177653304808** | **5.71727651177206** | **1.08244898863041e-08** | **1.98019194335855e-06** | **"-"** |  |
| **MSTRG.21640** | **8.72626541078953** | **6.53186878674849** | **1.23734914826363** | **5.27892131005596** | **1.29946591022675e-07** | **1.26879323772647e-05** | **Gm44026** |  |
| **MSTRG.16580** | **8.64242776938532** | **6.51667427982437** | **1.20216255506171** | **5.42079293052831** | **5.93352577376489e-08** | **6.87538363002215e-06** | **Dcst1** |  |
| **MSTRG.8579** | **15.4594957934567** | **5.62339396526701** | **1.97210722918289** | **2.85146460702188** | **0.00435183307027717** | **0.0337802700188787** | **Tnfsf11** |  |
| **MSTRG.13120** | **25.1005321163793** | **4.57990140837054** | **0.698694788034452** | **6.5549385608766** | **5.56647898188622e-11** | **3.24458252171459e-08** | **"-"** |  |
| **MSTRG.20809** | **14.9462497155835** | **4.47202782863398** | **1.03976928025435** | **4.3009809133234** | **1.70043707906681e-05** | **0.000565880747679066** | **Gm43123** |  |
| **MSTRG.15407** | **14.0264744330534** | **4.22957821473046** | **0.85146800370494** | **4.96739536462505** | **6.78581510243461e-07** | **4.51644129741625e-05** | **Efcab8** |  |
| **MSTRG.26974** | **12.0432513240191** | **4.19191017818445** | **0.937389364577691** | **4.47189859047843** | **7.7528192656178e-06** | **0.00031067808036283** | **Mpzl2** |  |
| **MSTRG.11744** | **13.5694879105182** | **4.15294686084294** | **0.795196945205048** | **5.22253875078968** | **1.76486609103293e-07** | **1.64792229422419e-05** | **Gm38220** |  |
| **MSTRG.2671** | **189.388011629468** | **4.12969517721483** | **0.796617626747285** | **5.18403690623446** | **2.17133937465075e-07** | **1.94069835096639e-05** | **Aire** |  |
| **MSTRG.21035** | **8.05614099160924** | **3.81365416036069** | **1.05002879084035** | **3.63195199372445** | **0.000281285457116942** | **0.00462438099798665** | **Ankrd61** |  |
| **MSTRG.23055** | **102.980253103256** | **3.80523702659945** | **1.11850776996973** | **3.40206579584372** | **0.000668785345633042** | **0.00888595166743073** | **Prx** |  |
| **ENSMUSG00000078954** | **8.05275545458696** | **3.6253630802909** | **0.901636885685067** | **4.02086819855017** | **5.79840421803561e-05** | **0.0014579386292015** | **Arhgap8** |  |
| **ENSMUSG00000030244** | **13.2472701047703** | **3.61777647613058** | **0.936045357865855** | **3.86495851480847** | **0.000111108082748597** | **0.00239808816816626** | **Gys2** |  |
| MSTRG.26501 | 9.39065489007704 | -3.51678241981554 | 1.00946177672928 | -3.4838192994391 | 0.00049431310439579 | 0.00704304634300223 | 5033426O07Rik |  |
| **ENSMUSG00000004814** | **8.00927796537655** | **3.50855275176159** | **1.07075481952755** | **3.27670974510268** | **0.00105024274218917** | **0.0122879678503702** | **Ccl24** |  |
| **MSTRG.26653** | **134.37526991842** | **3.38726682766073** | **0.677719663913142** | **4.99803533529292** | **5.79173737875384e-07** | **4.03637929276558e-05** | **"-"** |  |
| **MSTRG.9182** | **10.2588065575084** | **3.36536588628413** | **0.896766973683263** | **3.75277634552225** | **0.000174886874634116** | **0.00333064260751209** | **Psca** |  |
| **MSTRG.26581** | **7.79706580628783** | **3.29440476838085** | **1.21211689545836** | **2.71789361300427** | **0.00656989589651508** | **0.0448127473650594** | **Dpep1** |  |
| MSTRG.11327 | 45.5887770346726 | -3.22659071720407 | 0.506880411621512 | -6.36558573428039 | 1.94545867125525e-10 | 8.70253431199879e-08 | "-" |  |
| **MSTRG.576** | **8.48044168926316** | **3.21656701474749** | **1.08942697993026** | **2.95253107734985** | **0.00315180342644356** | **0.0270044271303527** | **4933402D24Rik** |  |
| ENSMUSG00000102166 | 9.55884048262347 | -3.18324196057103 | 0.903607094818427 | -3.52281647501969 | 0.000426986920826344 | 0.0063865423188917 | Gm36947 |  |
| **MSTRG.14167** | **354.824174663778** | **3.06977315590769** | **0.877316690824359** | **3.49904793561287** | **3.49904793561287** | **0.000466922629707377** | **Lcn2** |  |
| MSTRG.24838 | 30.0970860700283 | -3.05108488697996 | 0.648787549977973 | -4.70274882291368 | 2.56682115134225e-06 | 0.000134165230559968 | Cyp2e1 |  |
| **MSTRG.12893** | **58.5614800234039** | **3.02349881907381** | **0.87503210423768** | **3.45530044489951** | **0.000549679925484791** | **0.00767278183359939** | **"-"** |  |
| **MSTRG.18628** | **13.0702822158306** | **2.97805658295953** | **0.89219287914604** | **3.33790669323652** | **0.000844120902592758** | **0.0104462330209358** | **"-"** |  |
| **MSTRG.16670** | **23.5086429761571** | **2.97673047156306** | **0.684205472455281** | **4.35063820942709** | **1.35741863125524e-05** | **0.000477329933678144** | **Oaz3** |  |
| **MSTRG.11610** | **3847.51608494016** | **2.94870691641729** | **0.49559672503202** | **5.94981114176406** | **2.68452063063433e-09** | **7.17177143475714e-07** | **"-"** |  |
| **MSTRG.16940** | **24.0318335413355** | **2.92725581814816** | **0.891747409466339** | **3.28260647249871** | **0.00102852136446593** | **0.0121074715088752** | **Chil3** |  |
| **MSTRG.23234** | **195.507096877342** | **2.92445622555158** | **0.703223930510955** | **4.15864150616533** | **3.2014588217931e-05** | **0.000931619673785027** | **Gm17077** |  |
| MSTRG.4741 | 18.4399733002225 | -2.91319309298568 | 1.00694019394159 | -2.89311431851997 | 0.00381442435553857 | 0.0308673919237684 | Top2a |  |
| **MSTRG.28139** | **18.5659853980421** | **2.89186627049241** | **0.955822543159758** | **3.02552632932519** | **0.00248200793764283** | **0.0225301664372628** | **Gm47173** |  |
| MSTRG.11660 | 14.9009664076258 | -2.86464263300937 | 0.7537774634203 | -3.80038243649647 | 0.000144472947716214 | 0.00291598861418822 | A330072L02Rik |  |
| **MSTRG.25561** | **433.316560025199** | **2.82909690028611** | **0.444830304119914** | **6.35994642020492** | **2.01824136913832e-10** | **8.82292562167629e-08** | **Il12rb1** |  |
| **MSTRG.19127** | **19.5994121239554** | **2.7786405864669** | **0.865179280292004** | **3.21163561097891** | **0.00131981662851261** | **0.0144324461906993** | **Steap4** |  |
| **MSTRG.5037** | **48.231232175499** | **2.76929915263126** | **0.675019905994042** | **4.10254442578721** | **4.08631361144847e-05** | **0.0011212588062227** | **Cd300lf** |  |
| **MSTRG.28138** | **45.4250899248085** | **2.76795444754825** | **0.614112925278021** | **4.50724017296191** | **6.56762791831083e-06** | **0.000272069188230985** | **"-"** |  |
| **ENSMUSG00000111097** | **27.6863283180315** | **2.7351730149102** | **0.531877852442681** | **5.14248337724301** | **2.71130437999767e-07** | **2.27737728162687e-05** | **Gm34069** |  |
| **MSTRG.15365** | **26.6791984093613** | **2.72024057440844** | **0.7907616666931** | **3.44002584974088** | **0.000581658628996958** | **0.00800873566840121** | **Trib3** |  |
| **MSTRG.23507** | **35.7270300214578** | **2.71422278678483** | **0.455799596479599** | **5.95486000371287** | **2.60294549303552e-09** | **7.05178261387863e-07** | **"-"** |  |
| **MSTRG.11117** | **3587.20478373287** | **2.69227335681518** | **0.505342590237198** | **5.32762013103127** | **9.95079635813224e-08** | **1.02905144058427e-05** | **Cdkn1a** |  |
| **MSTRG.17177** | **36.3527632846104** | **2.66440744134047** | **0.512182619802918** | **5.20206531483967** | **1.9708593144359e-07** | **1.80251435881616e-05** | **Rrh** |  |
| **MSTRG.5154** | **20.5031421937356** | **2.65934991939307** | **0.939583324302095** | **2.83035027400937** | **0.00464970684447026** | **0.0354346716138611** | **"-"** |  |
| **MSTRG.18625** | **329.366110299398** | **2.65660722843674** | **0.509310711392017** | **5.21608355963271** | **1.8274576249947e-07** | **1.69023737057618e-05** | **Map3k6** |  |
| MSTRG.398 | 25.9262937772783 | -2.58058350084312 | 0.566702911284022 | -4.55367962553094 | 5.27156188890827e-06 | 0.000230451120302615 | Gm28322 |  |
| **MSTRG.10771** | **58.0932756070016** | **2.55749527098155** | **0.432389800605958** | **5.91479093030742** | **3.32297396237268e-09** | **8.6374870494917e-07** | **"-"** |  |
| MSTRG.21193 | 18.431010480828 | -2.55516322955973 | 0.69068180476065 | -3.69947957503413 | 0.000216042025402837 | 0.003847748480207 | "-" |  |
| **MSTRG.20602** | **28.0624149924177** | **2.54952722778866** | **0.864523399852468** | **2.94905519992142** | **0.00318747038313128** | **0.0272372247088095** | **Hcar2** |  |
| **MSTRG.3809** | **159.071188146587** | **2.54502733842502** | **0.328159330692891** | **7.75546236351509** | **8.80221809911572e-15** | **2.82184441894152e-11** | **"-"** |  |
| **MSTRG.23198** | **46.6526324901207** | **2.50215389727993** | **0.433175750319876** | **5.77630187154342** | **7.63603231500555e-09** | **1.56254342105459e-06** | **"-"** |  |
| **MSTRG.15812** | **57.0774799414985** | **2.45760470296651** | **0.552332491580357** | **4.4495023204858** | **8.60695000565705e-06** | **0.000336151757450091** | **Slc17a9** |  |
| MSTRG.21406 | 19.2903505865147 | -2.45605160213334 | 0.595588785704804 | -4.12373715067002 | 3.72774272978528e-05 | 0.00105136556315865 | "-" |  |
| **MSTRG.9329** | **207.829553678279** | **2.43629741131547** | **0.553574178352881** | **4.40103152673141** | **1.07737467721406e-05** | **0.000399292907826831** | **Maff** |  |
| **MSTRG.17698** | **17.9947716713384** | **2.42440572056716** | **0.552765573720772** | **4.38595642678689** | **1.15477238197626e-05** | **0.000419887462520103** | **Gm21104** |  |
| **MSTRG.22895** | **1473.56287546576** | **2.42404234567919** | **0.314856099505862** | **7.69888958633327** | **1.37253701307324e-14** | **3.77153563520912e-11** | **"-"** |  |
| **MSTRG.11211** | **141.57918569384** | **2.42348702193326** | **0.584471081717006** | **4.14646181435318** | **3.3765246082663e-05** | **0.000968946581876604** | **"-"** |  |
| **MSTRG.27581** | **203.846641100115** | **2.41814975710226** | **0.330782598781507** | **7.31038986334201** | **2.66368401251658e-13** | **3.41573079871709e-10** | **"-"** |  |
| ENSMUSG00000087278 | 46.1972607119889 | -2.39864227180661 | 0.645089925437081 | -3.7183068239384 | 0.000200562578153852 | 0.00363602374249703 | A930006I01Rik |  |
| **MSTRG.15290** | **20.7453655329276** | **2.39683827500944** | **0.65437456767389** | **3.66279252497465** | **0.000249480599917015** | **0.00426556385724781** | **"-"** |  |
| ENSMUSG00000026285 | 10.0826002779849 | -2.39056157429774 | 0.856368828379341 | -2.79150932994821 | 0.00524628451210715 | 0.0386489017963926 | Pdcd1 |  |
| **MSTRG.21693** | **72.1653022566199** | **2.37529335369154** | **0.454606412393803** | **5.2249446750741** | **1.7420718927035e-07** | **1.63731361092429e-05** | **A530053G22Rik** |  |
| **MSTRG.23579** | **31.0099704032296** | **2.37113285399435** | **0.625669876260709** | **3.78975070394203** | **0.000150798548892384** | **0.00300581356263731** | **Gm44829** |  |
| MSTRG.6512 | 7.74533453704415 | -2.36756626722004 | 0.790199525196387 | -2.99616260416208 | 0.00273400603080184 | 0.0242679307810214 | Gm47826 |  |
| **MSTRG.17899** | **11.2605158090239** | **2.34721200980669** | **0.814933453015005** | **2.88024991626338** | **0.00397360053700951** | **0.0317541364060565** | **"-"** |  |
| **MSTRG.4858** | **27.3019134643593** | **2.3336788697392** | **0.595570033475175** | **3.91839538353213** | **8.91404027087611e-05** | **0.00204793647278383** | **"-"** |  |
| **MSTRG.3560** | **55.8332979198096** | **2.32277922558495** | **0.467127449529547** | **4.97247427425698** | **6.6103745389879e-07** | **4.44582357543469e-05** | **1700093K21Rik** |  |
| **MSTRG.17741** | **8.10835383531738** | **2.31186291765151** | **0.785474302429274** | **2.94326995867529** | **0.00324765084380859** | **0.0275985661652162** | **Ccl19** |  |
| **ENSMUSG00000097476** | **23.320010889192** | **2.30016854415349** | **0.633068652843345** | **3.63336351250782** | **0.000279750457641692** | **0.00461096833996397** | **Gm26583** |  |
| **ENSMUSG00000026774** | **17.8696921998549** | **2.29396989719869** | **0.654789950444552** | **3.50336760000861** | **0.000459414979893495** | **0.00669458116534196** | **Potegl** |  |
| **MSTRG.17608** | **653.983736445007** | **2.29083856000397** | **2.29083856000397** | **0.401057713415323** | **5.71199227287182** | **1.11661159625313e-08** | **"-"** |  |
| MSTRG.12140 | 683.188019283059 | -2.28540980512405 | 0.595187705786112 | -3.8398135292554 | 0.000123127794930033 | 0.00257430775595563 | Gpr17 |  |
| **MSTRG.24457** | **40.2418269386854** | **2.2815872530325** | **0.605960018442048** | **3.76524388341423** | **0.000166386597721343** | **0.00322911717264309** | **Acsm3** |  |
| **ENSMUSG00000011305** | **43.3855563550169** | **2.27319087597494** | **0.418331503612301** | **5.43394617987384** | **5.51212126720131e-08** | **6.50464126224645e-06** | **Plin5** |  |
| **MSTRG.16491** | **12.4989690075955** | **2.25875818865952** | **0.783823132515293** | **2.88171922332931** | **0.00395511997563375** | **0.0316578084549228** | **Crabp2** |  |
| **ENSMUSG00000026114** | **21.7006606644427** | **2.23559373179595** | **0.761614689036444** | **2.93533431534035** | **0.00333188494754306** | **0.0280844903444307** | **Cnga3** |  |
| ENSMUSG00000116597 | 9.33125246433086 | -2.22582116864111 | 0.715532183688966 | -3.11072124969385 | 0.00186631024592491 | 0.018362392624228 | Gm536 |  |
| **MSTRG.5325** | **28.1381933546451** | **2.19718618299467** | **0.604211029619013** | **3.63645493922895** | **0.000276415970270786** | **0.0045716777198268** | **"-"** |  |
| **MSTRG.22978** | **52.3041035340812** | **2.19181102398707** | **0.670925166561249** | **3.26684872356324** | **0.00108751775982694** | **0.0125938615955877** | **"-"** |  |
| **MSTRG.18799** | **39.9286818182787** | **2.19010622395303** | **0.477413605076267** | **4.58743990675164** | **4.48714528825407e-06** | **0.000203266221667737** | **Tmem82** |  |
| **ENSMUSG00000056032** | **32.8389405639133** | **2.18567176233343** | **0.563413651135197** | **3.87933760200807** | **0.000104741301423445** | **0.00228423915292514** | **BC018473** |  |
| MSTRG.13913 | 11.6525045813347 | -2.17827237051569 | 0.80093736181874 | -2.71965383855905 | 0.0065350288756185 | 0.044638238786406 | Gm13339 |  |
| MSTRG.9987 | 346.330333923817 | -2.1651541105536 | 0.264960354513918 | -8.17161538950109 | 3.04287273893667e-16 | 1.95098857111489e-12 | "-" |  |
| **MSTRG.19814** | **92.0126842705435** | **2.15551525538808** | **0.387652851967531** | **5.56042666640467** | **2.69115915638327e-08** | **3.69746045521658e-06** | **Gm43279** |  |
| **MSTRG.8273** | **37.7761935248783** | **2.14872686939033** | **0.596439115581652** | **3.60259213934162** | **0.000315059669116117** | **0.00505435590946498** | **Gm15932** |  |
| ENSMUSG00000074813 | 8.37368477037579 | -2.14386390783381 | 0.785062883929893 | -2.73081806784953 | 0.00631773370630709 | 0.04375664892052 | Morrbid |  |
| **MSTRG.23267** | **9.01117909707192** | **2.14219028606086** | **0.762802071692969** | **2.80831734149131** | **0.00498011250587274** | **0.0372313146549622** | **Gm45094** |  |
| **MSTRG.11108** | **53.7032631147158** | **2.13070377913503** | **0.357080125706646** | **5.96701867660223** | **2.4162776101001e-09** | **6.73581156960513e-07** | **Gm49864** |  |
| MSTRG.3180 | 14.2458570953612 | -2.10722635534403 | 0.571638026754782 | -3.68629492216754 | 0.00022754264410802 | 0.00400437580916538 | "-" |  |
| **MSTRG.20810** | **65.8908747744498** | **2.10377859177713** | **0.480077472483358** | **4.38216478039398** | **1.17505880138972e-05** | **0.000425654539448801** | **"-"** |  |
| **MSTRG.15402** | **25.4519687140506** | **2.09296823488401** | **0.566351897841676** | **3.69552612582416** | **0.000219431913562672** | **0.00390089912881516** | **"-"** |  |
| **MSTRG.27613** | **62.5580815204855** | **2.07482219871525** | **0.525029275947968** | **3.95182191501427** | **7.75584503337378e-05** | **0.0018459805833415** | **"-"** |  |
| **MSTRG.10831** | **18.8645369766219** | **2.07458500170172** | **0.609100969875326** | **3.40597881846478** | **0.000659272939348052** | **0.00878802147495481** | **Dynlt2a3** |  |
| MSTRG.4562 | 46.0749237324629 | -2.07073857602503 | 0.430667675236566 | -4.8082052475556 | 1.52291364471543e-06 | 8.80525485002324e-05 | "-" |  |
| **MSTRG.21565** | **23.2047550705072** | **2.06864920337867** | **0.60370927700449** | **3.4265652064235** | **0.000611266978260603** | **0.0082976149095573** | **Gm7932** |  |
| **MSTRG.14114** | **17.3033386795616** | **2.06596511444859** | **2.06596511444859** | **0.673295799039672** | **3.06843606835999** | **0.00215182365472035** | **1700084E18Rik** |  |
| MSTRG.19073 | 23.9728931169052 | -2.05655669424342 | 0.757935810494087 | -2.71336525569729 | 0.0066603648746512 | 0.045157602525173 | Perm1 |  |
| MSTRG.23150 | 76.0332651285832 | -2.05391877868752 | 0.354974741076465 | -5.7860983924067 | 7.204002778668e-09 | 1.50618471138781e-06 | 4930432E11Rik |  |
| **MSTRG.15815** | **39.5072461759959** | **2.04729934332286** | **0.587001332669682** | **3.48772520500378** | **0.000487148473303885** | **0.00699276185373151** | **Gm14342** |  |
| MSTRG.4212 | 34.7458727803996 | -2.04566902452798 | 0.474071281861634 | -4.31510851383959 | 1.59524522406948e-05 | 0.000537382519876995 | Gm12324 |  |
| **ENSMUSG00000059901** | **32.2622124757081** | **2.03914607677936** | **0.671930215603196** | **3.03475871366911** | **0.00240728190896928** | **0.0220600607522745** | **Adamts14** |  |
| MSTRG.716 | 37.7908727307933 | -2.03269629367218 | 0.590057362214776 | -3.44491302683263 | 0.000571243164601122 | 0.00791062798495507 | Gm816 |  |
| **MSTRG.17823** | **15.9686691316404** | **2.02721029687794** | **0.690909648162939** | **2.93411780001639** | **0.0033449723007371** | **0.0281701148006472** | **"-"** |  |
| MSTRG.17505 | 28.0481502693703 | -2.01357780547561 | 0.565894303547194 | -3.55822243280044 | 0.000373373070582433 | 0.00577317605518738 | Esrp1 |  |
| **MSTRG.27711** | **18.3633407284999** | **2.01222780489787** | **0.555165310451752** | **3.62455608629523** | **0.000289458201463657** | **0.00472746427165766** | **Gm37314** |  |
| MSTRG.13225 | 16.9177877688692 | -2.01183675269988 | 0.659997263251888 | -3.04825014392834 | 0.00230178236897953 | 0.0214094699551843 | Ifit1bl2 |  |
| **MSTRG.17338** | **252.179619308003** | **2.01054435275064** | **0.490070843391146** | **4.10255859915734** | **4.08606321773893e-05** | **0.0011212588062227** | **Ccn1** |  |
| **MSTRG.19614** | **28.3409809488872** | **2.00111522693959** | **0.519195597999832** | **3.85426077310508** | **0.000116079821116502** | **0.00245632052714623** | **Bst1** |  |
| MSTRG.17143 | 6064.11012829409 | -1.99429547052258 | 0.405120794881416 | -4.92271810215602 | 8.5350357207164e-07 | 5.49068267852775e-05 | "-" |  |
| MSTRG.8703 | 14.2057282947891 | -1.98666742646432 | 0.684567321230073 | -2.90207750918427 | 0.00370696809257881 | 0.0303419281960653 | Dct |  |
| **MSTRG.11242** | **99.6177737551233** | **1.98041622619143** | **0.342102247175245** | **5.78896000404504** | **7.08235577506467e-09** | **1.49702322344362e-06** | **Angptl4** |  |
| MSTRG.18426 | 40.4882376915276 | -1.9775451357534 | 0.483775219092481 | -4.08773549720693 | 4.35604398364404e-05 | 0.0011735084877506 | "-" |  |
| **ENSMUSG00000038763** | **29.99538020195** | **1.97304955414467** | **0.570418107651874** | **3.45895322690005** | **0.000542279066263912** | **0.00760337790251277** | **Alpk3** |  |
| MSTRG.10737 | 155.234621339778 | -1.97229894618168 | 0.300260254041042 | -6.56863144434724 | 5.07797958330208e-11 | 3.05234179015049e-08 | "-" |  |
| **MSTRG.28662** | **23.3010154783295** | **1.97035751605969** | **0.621194050048371** | **3.17188729658029** | **0.00151451770766013** | **0.0158929340462862** | **Hsf3** |  |
| MSTRG.25517 | 50.4969333202164 | -1.96674829885413 | 0.380904487754586 | -5.16336342070428 | 2.42551574699132e-07 | 2.11107671463249e-05 | Tm6sf2 |  |
| **MSTRG.24664** | **72.0264906554247** | **1.96590369617198** | **0.458644519849068** | **4.28633421112047** | **1.81645649177077e-05** | **0.000598279805123471** | **Trim72** |  |
| **MSTRG.7663** | **22.1018042504531** | **1.9648628316719** | **0.696888588955751** | **2.81947912881762** | **0.00481016590118458** | **0.0362979761119205** | **Gm48239** |  |
| MSTRG.20785 | 13.2939250954507 | -1.96313295076727 | 0.641044007774854 | -3.06239965892756 | 0.00219570076182302 | 0.0207664712584945 | "-" |  |
| **MSTRG.24662** | **124.822385067342** | **1.9599582179792** | **0.364844650603973** | **5.37203495990593** | **7.78530098460503e-08** | **8.41293620443134e-06** | **Gm45205** |  |
| MSTRG.11326 | 39.1510028954614 | -1.9494838116523 | 0.371817596496008 | -5.24311874968842 | 1.5788485197975e-07 | 1.49601730434999e-05 | Ly6g6e |  |
| **ENSMUSG00000026380** | **48.703327101817** | **1.94781270151258** | **0.643709798813493** | **3.02591743220757** | **0.00247879982468364** | **0.02251694759068** | **Tfcp2l1** |  |
| **MSTRG.18529** | **39.5420194378865** | **1.93024184531114** | **0.653198450681196** | **2.95506188555432** | **0.00312606338374174** | **0.0268436737438716** | **"-"** |  |
| **MSTRG.14652** | **63.1877408445741** | **1.93020200142088** | **0.446925192383679** | **4.31884806297476** | **1.56845709968501e-05** | **0.000533969421459136** | **Slc43a3** |  |
| **MSTRG.16576** | **12.9802450751257** | **1.92766380476808** | **0.671909007380399** | **2.8689357987379** | **0.00411855387771153** | **0.0324673704253202** | **Efna4** |  |
| ENSMUSG00000035896 | 16.2226537819273 | -1.92592979532757 | 0.673090059270498 | -2.86132556676726 | 0.00421873583061607 | 0.0329818685733367 | Rnase1 |  |
| **MSTRG.2123** | **9295.2282517662** | **1.91019794929709** | **0.30463190360998** | **6.27051181002604** | **3.59863252624558e-10** | **1.33114801235257e-07** | **Sgk1** |  |
| ENSMUSG00000033715 | 41.2813371331524 | -1.90098724377819 | 0.462794315573668 | -4.10762876683517 | 3.9974192567938e-05 | 0.0011044478526463 | Akr1c14 |  |
| **ENSMUSG00000049537** | **17.9291578821582** | **1.89303906473391** | **0.64628891125331** | **2.92909104855729** | **0.00339954826735234** | **0.0285049306549792** | **Tecrl** |  |
| **ENSMUSG00000081402** | **16.1937682505778** | **1.8898618425173** | **0.61726536570466** | **3.06166836423725** | **0.00220107168174553** | **0.0207785034360354** | **Gm15455** |  |
| MSTRG.5687 | 224.705943010033 | -1.8786697955728 | 0.351644701450839 | -5.34252268787689 | 9.16618945391108e-08 | 9.63451661999889e-06 | Gm38103 |  |
| MSTRG.11390 | 63.10101318477 | -1.87666892291667 | 0.383159507290286 | -4.89787904830684 | 9.68766328327363e-07 | 6.08961448541726e-05 | H2-T24 |  |
| **MSTRG.2159** | **11.0019637430128** | **1.87296301564266** | **0.676233409759101** | **2.76969902494152** | **0.00561081132682648** | **0.0403755914221876** | **Gm4739** |  |
| **MSTRG.9948** | **262.749030064622** | **1.86945847108613** | **0.3234380694989** | **5.7799580426091** | **7.4719257398023e-09** | **1.54540313553868e-06** | **"-"** |  |
| **MSTRG.22038** | **31.4724357953465** | **1.86641611301722** | **0.567277038663651** | **3.29013160379976** | **0.00100140534760717** | **0.0118396775942241** | **Gm44117** |  |
| **MSTRG.17643** | **12.4897254943382** | **1.85759931920837** | **0.686320922832949** | **2.70660453063372** | **0.00679751787450498** | **0.0457008935044052** | **"-"** |  |
| MSTRG.5614 | 87.6896177169352 | -1.8460463414931 | 0.301908395889315 | -6.11459093760975 | 9.68049553580616e-10 | 3.15600562086833e-07 | Gm48508 |  |
| ENSMUSG00000082361 | 43.5594801275695 | -1.84514242340882 | 0.48257769221534 | -3.82351371224483 | 0.00013156327145274 | 0.00272696069654466 | Btc |  |
| **ENSMUSG00000097848** | **20.4095588285506** | **1.82406157303343** | **0.615755451977185** | **2.96231493716603** | **0.00305335296710582** | **0.0263531396888174** | **Gm807** |  |
| ENSMUSG00000054488 | 8.74401161971228 | -1.82363761839251 | 0.624771413914168 | -2.91888773682441 | 0.00351282706752698 | 0.0291749691899315 | Gm9946 |  |
| **MSTRG.18962** | **44.3877812769923** | **1.80477888753911** | **0.482363716479374** | **3.74153118462482** | **0.000182902476387548** | **0.00345253104348822** | **"-"** |  |
| ENSMUSG00000113186 | 14.2773942981422 | -1.80469420234461 | 0.656537513984826 | -2.74880591573679 | 0.00598127912009049 | 0.0422046602622673 | A330076C08Rik |  |
| **MSTRG.9474** | **498.422296186167** | **1.8043381483266** | **0.174403552380245** | **10.3457648866732** | **4.37409310839585e-25** | **8.41356809399942e-21** | **"-"** |  |
| **MSTRG.1772** | **70.3737334590074** | **1.79860812932267** | **0.391690580278718** | **4.59191060464823** | **4.39206506748052e-06** | **0.000202108544432985** | **"-"** |  |
| **MSTRG.28087** | **60.5167731256234** | **1.79248460655923** | **0.47760681246578** | **3.75305493928158** | **0.000174692549885766** | **0.00333023904564192** | **"-"** |  |
| **MSTRG.7082** | **9.46968526553629** | **1.77899623313924** | **0.638041020612194** | **2.78821607963749** | **0.00529991807520849** | **0.0388950492852481** | **Gm48735** |  |
| **ENSMUSG00000043747** | **31.5976358836715** | **1.77771358706547** | **0.553632884804485** | **3.21099709908538** | **0.00132275270083112** | **0.0144481250428657** | **1520401A03Rik** |  |
| **MSTRG.6114** | **14.5988688549839** | **1.76998616079985** | **0.601442728616198** | **2.94290058984044** | **0.00325152810459869** | **0.0276128667072653** | **Gm19951** |  |
| MSTRG.674 | 44.2667320712274 | -1.76548580669948 | 0.414239637751284 | -4.26199147981948 | 2.02613170054779e-05 | 0.000647540481102225 | Prkag3 |  |
| **MSTRG.25562** | **2321.41998768131** | **1.76460493327281** | **0.322994700934296** | **5.46326279709391** | **4.67461770592198e-08** | **5.76386356239803e-06** | **"-"** |  |
| **MSTRG.16520** | **32.1586178690539** | **1.76112606712209** | **0.427802111853533** | **4.11668390203049** | **3.8436265510021e-05** | **0.00107459530099601** | **"-"** |  |
| **MSTRG.19451** | **67.0495636440173** | **1.75273482651903** | **0.351343386908209** | **4.98866605101905** | **6.07976401789719e-07** | **4.17658074586616e-05** | **"-"** |  |
| ENSMUSG00000046500 | 27.2113549692066 | -1.75008509061345 | 0.516379896487218 | -3.38914257220076 | 0.000701115465164099 | 0.00918036485529711 | Tafa4 |  |
| MSTRG.21920 | 70.0679890276013 | -1.74672277142276 | 0.367180096795956 | -4.75712814138022 | 1.9636652828186e-06 | 0.000106397469619763 | Nat8f7 |  |
| MSTRG.23148 | 13.9470525409287 | -1.74266968226643 | 0.61262777476632 | -2.84458157799188 | 0.00444697968522817 | 0.0342823915463644 | "-" |  |
| **ENSMUSG00000063672** | **14.8981402630466** | **1.73936985399275** | **0.646084125705584** | **2.69217240416362** | **0.00709882381505128** | **0.0468585424077433** | **Nkx6-3** |  |
| **MSTRG.25244** | **21.025442453145** | **1.7362451131881** | **0.586175826555209** | **2.9619868894825** | **0.00305660798782176** | **0.0263531396888174** | **Proscos** |  |
| MSTRG.11496 | 45.8909390964968 | -1.73424906915641 | 0.628093869299082 | -2.76113038818821 | 0.00576016746765109 | 0.0410358597186181 | "-" |  |
| MSTRG.975 | 49.8790435927093 | -1.71618384065136 | 0.37626418362968 | -4.56111401328708 | 5.08829416080801e-06 | 0.000224996179731361 | Panct2 |  |
| **MSTRG.3873** | **1560.74262260151** | **1.71474445714537** | **0.543383214683102** | **3.15568168248517** | **0.00160123506760087** | **0.0165323438139037** | **Gpx3** |  |
| **MSTRG.21079** | **79.47036821377** | **1.71386968519753** | **0.317317302558535** | **5.40112269762338** | **6.62251075617375e-08** | **7.46853843886625e-06** | **Rasl11a** |  |
| **MSTRG.16557** | **88.7206197578481** | **1.71319256625319** | **0.390606521200798** | **4.38598045159747** | **1.15464491445353e-05** | **0.000419887462520103** | **Muc1** |  |
| **ENSMUSG00000055972** | **12.8664637363038** | **1.71282082576452** | **0.641296228146493** | **2.67087307018007** | **0.00756542488243099** | **0.0489711004859014** | **2810407A14Rik** |  |
| **MSTRG.22302** | **435.508772457957** | **1.71066263169271** | **0.224522994035449** | **7.61909771888495** | **2.55455026181702e-14** | **5.45964158733892e-11** | **Zfand4** |  |
| MSTRG.8611 | 32.1399004124228 | -1.70950628148139 | 0.609774918305421 | -2.80350376862358 | 0.00505506414910791 | 0.037687658491508 | 9630013A20Rik |  |
| MSTRG.23407 | 47.5926206888025 | -1.70595174628083 | 0.48276242286053 | -3.53372935733584 | 0.000409740410811945 | 0.00618145631526884 | "-" |  |
| **ENSMUSG00000086015** | **26.5578587545259** | **1.69824800709885** | **0.440742482386005** | **3.85315252095784** | **0.000116606703101263** | **0.00246475816939867** | **4833417C18Rik** |  |
| MSTRG.1157 | 126.344153776415 | -1.69751981571586 | 0.353441860598386 | -4.80282616451236 | 1.56441695382675e-06 | 8.98255525577837e-05 | Rab7b |  |
| **MSTRG.16579** | **334.96852044376** | **1.69341932859296** | **0.282710334146192** | **5.98994491555189** | **2.09912122243691e-09** | **6.2117841097806e-07** | **"-"** |  |
| **MSTRG.15569** | **137.912035466264** | **1.6931158621721** | **0.269373301780674** | **6.28538853323574** | **3.2703474440234e-10** | **1.2581026617158e-07** | **"-"** |  |
| **MSTRG.1394** | **57.2457650530727** | **1.68749398746547** | **0.459550617693274** | **3.67205248452476** | **0.000240610250637972** | **0.00416574092801206** | **Teddm2** |  |
| **MSTRG.17869** | **37.4032900180294** | **1.67844394270116** | **0.378450711569507** | **4.43503973275763** | **9.205530907087e-06** | **0.000351326164678211** | **"-"** |  |
| **ENSMUSG00000024481** | **11.22314798433** | **1.67808024852406** | **0.60473770168091** | **2.77488941711377** | **0.00552204804563644** | **0.0399160444035389** | **Lvrn** |  |
| MSTRG.6716 | 1098.36634967978 | -1.67258586424724 | 0.221615944587544 | -7.54722710660617 | 4.44622881286161e-14 | 7.77483738321756e-11 | Serpinb1a |  |
| **ENSMUSG00000021765** | **16.4194143401735** | **1.67221758140057** | **0.562594696449391** | **2.9723308661709** | **0.00295547997147137** | **0.0257552537369058** | **Fst** |  |
| **MSTRG.10170** | **68.4831741045606** | **1.65975506191492** | **0.344355776519615** | **4.81988447729836** | **1.43641374165698e-06** | **8.4493634008477e-05** | **Gm15743** |  |
| **ENSMUSG00000044244** | **23.8758028730077** | **1.6579614719009** | **0.557932091187355** | **2.97161876523814** | **0.00296234274264347** | **0.0257947771184912** | **Il20rb** |  |
| **ENSMUSG00000115389** | **17.1728171900134** | **1.65162115355018** | **0.556684717082148** | **2.96688790417513** | **0.00300830615799151** | **0.0261004821601113** | **Gm48936** |  |
| **MSTRG.27915** | **29.5005746366554** | **1.64733323949063** | **0.514509051206411** | **3.20175755048039** | **0.00136591909139323** | **0.0147272722662269** | **Gm7628** |  |
| MSTRG.6011 | 52.8425985721262 | -1.64579579372028 | 0.304534574532525 | -5.404298662136 | 6.50624584979349e-08 | 7.40518573495726e-06 | "-" |  |
| **MSTRG.4814** | **1334.08037992514** | **1.6404875681945** | **0.366526887261361** | **4.47576323923192** | **7.61388230487413e-06** | **0.000306387083962874** | **Arl4d** |  |
| MSTRG.21926 | 169.132802866301 | -1.63959470793192 | 0.288662787582484 | -5.67996561546201 | 1.34721822396465e-08 | 2.24748711507941e-06 | Nat8f3 |  |
| MSTRG.21978 | 54.4069737501911 | -1.6386449555894 | 0.472416273422963 | -3.46864629305919 | 0.00052308764456182 | 0.00738194485924183 | Gkn3 |  |
| **MSTRG.337** | **230.586420824796** | **1.63617507599458** | **0.299809651765357** | **5.45737959522102** | **4.83212603099257e-08** | **5.88265469659127e-06** | **Il1r1** |  |
| **MSTRG.27436** | **98.857507355486** | **1.63552602930661** | **0.352110498677893** | **4.6449226462934** | **3.40203900561798e-06** | **0.000164570017324001** | **Gm7972** |  |
| MSTRG.21303 | 16.7873621926677 | -1.63397857934781 | 0.489353645595737 | -3.33905467764241 | 0.000840640160368103 | 0.0104308857123164 | Gm37940 |  |
| MSTRG.3928 | 1922.65747180309 | -1.63087791125002 | 0.29760990808837 | -5.47991806363437 | 4.25522849355558e-08 | 5.34962876297657e-06 | Gjc2 |  |
| **MSTRG.10285** | **56.381666518904** | **1.63069458772636** | **0.416049291969414** | **3.91947449305174** | **8.87422520159229e-05** | **0.00204793647278383** | **Pla1a** |  |
| **MSTRG.11534** | **73.0022722710331** | **1.62923535842443** | **0.3746702404195** | **4.348451471887** | **1.37102146653417e-05** | **0.000481233538481475** | **"-"** |  |
| **MSTRG.24065** | **65.1384199203285** | **1.62915450544314** | **0.426243435009095** | **3.82212222320415** | **0.000132308079196133** | **0.00273507772451122** | **Gm44907** |  |
| **MSTRG.6666** | **21.8588227536429** | **1.62602638936135** | **0.476881931217751** | **3.40970433752686** | **0.00065033339927885** | **0.00869295547958907** | **Gm11346** |  |
| **MSTRG.22557** | **274.604494002656** | **1.62486172784213** | **0.499258804127501** | **3.25454797072978** | **0.00113572977474605** | **0.0129802508718005** | **Apold1** |  |
| **MSTRG.16736** | **129.049119213451** | **1.62399256622892** | **0.369644004304293** | **4.39339620639983** | **1.11593479983051e-05** | **0.000411207009094633** | **Gm20632** |  |
| **MSTRG.12748** | **51.0485014133605** | **1.62248933624924** | **0.360319984533728** | **4.50291242754361** | **6.70285084775406e-06** | **0.000274464759836309** | **"-"** |  |
| **MSTRG.24886** | **888.436877449132** | **1.62210236492393** | **0.382652845498543** | **4.23909657018377** | **2.2442110919983e-05** | **0.000704082881670714** | **Tspan4** |  |
| **MSTRG.4514** | **121.542237045007** | **1.61992513096026** | **0.406218978771835** | **3.98781252382138** | **6.66853269380003e-05** | **0.0016402714368957** | **Tbx2** |  |
| **MSTRG.2434** | **3041.13524666073** | **1.61701987965115** | **0.285898954617867** | **5.65591392879509** | **1.55019143120058e-08** | **2.52053500349433e-06** | **Ddit4** |  |
| **MSTRG.5128** | **130.344964707412** | **1.61420862607289** | **0.435562333412798** | **3.70603356223424** | **0.000210530413239292** | **0.00377756762934495** | **Socs3** |  |
| **MSTRG.17748** | **20.7912332338989** | **1.61417983958007** | **0.551472317313593** | **2.92703693168731** | **0.00342208241656342** | **0.0286564019515008** | **"-"** |  |
| **MSTRG.12041** | **184.709237706848** | **1.61078223037069** | **0.280747898985379** | **5.73746851246989** | **9.61021392472565e-09** | **1.83022242417919e-06** | **Cabyr** |  |
| **MSTRG.25413** | **369.988346150112** | **1.61036964031067** | **0.405879994793668** | **3.96760042615382** | **7.25999225611023e-05** | **0.0017521449315719** | **"-"** |  |
| **ENSMUSG00000026073** | **16.9812456418648** | **1.60965528041004** | **0.57558740333549** | **2.79654361975644** | **0.00516524380314282** | **0.038212870982097** | **Il1r2** |  |
| **MSTRG.7182** | **15.3489846095444** | **1.59806883199992** | **0.466931235311342** | **3.42249288791818** | **0.000620497210743877** | **0.00838150551169837** | **Gm35161** |  |
| MSTRG.6416 | 14.0277751504574 | -1.59497931087989 | 0.553125080552249 | -2.8835779952112 | 0.00393185266339274 | 0.0315516003255567 | "-" |  |
| MSTRG.10760 | 65.3286897779852 | -1.58930848355571 | 0.353948545502718 | -4.49022464917435 | 7.1148095058668e-06 | 0.000288112338621785 | "-" |  |
| **MSTRG.10505** | **19.3449812964202** | **1.58651833763808** | **0.478183306813322** | **3.31780368539179** | **0.000907282275665551** | **0.0110164460748573** | **E330011O21Rik** |  |
| **ENSMUSG00000024215** | **45.520953203297** | **1.57719943231547** | **0.447869439881774** | **3.52156073147524** | **0.000429014385858664** | **0.00639697031937318** | **Spdef** |  |
| **MSTRG.7222** | **104.376642273011** | **1.57661133543883** | **0.321587056643799** | **4.90259574465752** | **9.45784912201951e-07** | **5.98426736388307e-05** | **Slc9a3** |  |
| **MSTRG.25906** | **92.7466653448038** | **1.57460224588633** | **0.353900119970318** | **4.44928429529323** | **8.61569100197009e-06** | **0.000336151757450091** | **Gm45774** |  |
| **MSTRG.2152** | **26.8325547827539** | **1.57175586685733** | **0.457413788044976** | **3.43617946799361** | **0.000589980012693042** | **0.00808857130730625** | **Gm40617** |  |
| MSTRG.3456 | 4170.4992790388 | -1.56947170366771 | 0.560505919172117 | -2.80009835754431 | 0.00510870378649106 | 0.0379508001712145 | Purb |  |
| **MSTRG.2058** | **21.200520435626** | **1.56503241964932** | **0.478760286499533** | **3.26892698450846** | **0.00107956155890407** | **0.0125319050003137** | **B230208H11Rik** |  |
| **MSTRG.8339** | **463.776664727153** | **1.56245731281688** | **0.442690700716937** | **3.52945591648182** | **0.000416415073721863** | **0.00624462706996232** | **Setdb2** |  |
| MSTRG.2253 | 69.8691292909545 | -1.55410560827461 | 0.424813293891492 | -3.65832621205958 | 0.000253867794625717 | 0.00431754821363896 | "-" |  |
| **MSTRG.15056** | **24.8188762520648** | **1.54770824904608** | **0.564605764492498** | **2.74121935406957** | **0.00612116275476215** | **0.0428147511228545** | **Slc12a1** |  |
| MSTRG.11566 | 17.6877282168884 | -1.54572852399997 | 0.493194834922938 | -3.13411336564684 | 0.00172374246865224 | 0.0174330712288162 | "-" |  |
| **MSTRG.24876** | **2189.33863807293** | **1.54507309835666** | **0.346789909893646** | **4.45535770873642** | **8.37534302530572e-06** | **0.000329713767781607** | **"-"** |  |
| **MSTRG.22389** | **68.7286414003698** | **1.54409397212316** | **0.375878167317559** | **4.10796397977177** | **3.9916233650477e-05** | **0.0011044478526463** | **Gm7298** |  |
| **MSTRG.23447** | **91.0758894584496** | **1.5429195323689** | **0.417726824621477** | **3.6936089363354** | **0.00022109373050689** | **0.00392318995046128** | **Plekha4** |  |
| **MSTRG.66** | **1473.23880860918** | **1.54185382860544** | **0.396245357126958** | **3.89115935587209** | **9.97663803191524e-05** | **0.00220161164565628** | **Sgk3** |  |
| **MSTRG.20867** | **236.362144584784** | **1.54175085290769** | **0.25207931040806** | **6.11613404690744** | **9.58726890789603e-10** | **3.15600562086833e-07** | **Tfr2** |  |
| **MSTRG.22473** | **814.117223284803** | **1.53572462486215** | **0.338114222552701** | **4.54202906126725** | **5.57153581901909e-06** | **0.000241370476303676** | **"-"** |  |
| **MSTRG.1392** | **24.0083863536503** | **1.5337440388928** | **0.504893023936436** | **3.03776040899684** | **0.00238343372219281** | **0.0219357395642587** | **A830008E24Rik** |  |
| ENSMUSG00000030074 | 12.4470515980129 | -1.53323076653008 | 0.541862594604215 | -2.82955638901404 | 0.00466125850864217 | 0.0354945793403532 | Gxylt2 |  |
| **MSTRG.16107** | **148.310951904059** | **1.52741618922904** | **0.341599936222755** | **4.47135970257625** | **7.77238418469836e-06** | **0.000310814573373541** | **"-"** |  |
| **MSTRG.9166** | **31.6929685936004** | **1.52636475190451** | **0.469903030100919** | **3.24825475497934** | **0.00116115257700907** | **0.0131768553503065** | **Mroh5** |  |
| MSTRG.10666 | 74.4587699924868 | -1.5234054919303 | 0.374756390271776 | -4.06505541059757 | 4.80210517627505e-05 | 0.00126014315232811 | "-" |  |
| **MSTRG.8908** | **20.7972223414133** | **1.52146297862789** | **0.564076672687028** | **2.69726271675137** | **0.00699120897445688** | **0.0464832024278182** | **"-"** |  |
| **MSTRG.22170** | **19.1682654432392** | **1.52143902395418** | **0.470319525099399** | **3.23490508635089** | **0.00121683173521428** | **0.0135843055292204** | **Gm44104** |  |
| **MSTRG.11102** | **1927.84680313051** | **1.51997946396113** | **0.287367659373013** | **5.28931984649026** | **1.22772038298291e-07** | **1.22358557340292e-05** | **Fkbp5** |  |
| MSTRG.37 | 79.5802074183112 | -1.51752388898696 | 0.278018923242332 | -5.45834747969377 | 4.80586435281425e-08 | 5.88265469659127e-06 | Gm38372 |  |
| **MSTRG.21958** | **21.9488130348832** | **1.5123709334346** | **0.495671349225237** | **3.05115665006365** | **0.00227961606798599** | **0.0212443871452086** | **Gm44214** |  |
| MSTRG.6458 | 35.958883408041 | -1.51148681340035 | 0.474353178565334 | -3.18641653877347 | 0.00144047042150645 | 0.015324916237653 | Ucn3 |  |
| MSTRG.368 | 27.7012057128473 | -1.51106167634496 | 0.496575221365667 | -3.04296632479825 | 0.00234258546457766 | 0.0216856612291136 | "-" |  |
| **MSTRG.26495** | **63.6614165943686** | **1.50974494153397** | **0.385544038643534** | **3.9158819491691** | **9.00743176878794e-05** | **0.00206259464372186** | **Foxf1** |  |
| **MSTRG.27172** | **139.417489688704** | **1.50686068181502** | **0.264771962900586** | **5.69116406928932** | **1.26176193563272e-08** | **2.12894656420135e-06** | **"-"** |  |
| **MSTRG.20959** | **19.2082593524496** | **1.50567771265234** | **0.465307589620471** | **3.23587610913557** | **0.00121270006063258** | **0.0135579384529338** | **Grifin** |  |
| **MSTRG.8820** | **49.2293967291372** | **1.5035583491129** | **0.306827913784306** | **4.90033103757915** | **9.56753086831051e-07** | **6.03381823776894e-05** | **Gm37310** |  |
| MSTRG.12479 | 165.724683626646 | -1.50040248041682 | 0.304623866593581 | -4.92542655043707 | 8.41765259694292e-07 | 5.43334052691937e-05 | Spink10 |  |
| **MSTRG.25742** | **43.1176635227662** | **1.4984968222296** | **0.298840837652269** | **5.01436428167575** | **5.32090943499072e-07** | **3.80474695100545e-05** | **Asf1b** |  |
| MSTRG.24721 | 247.383503167837 | -1.49634889753102 | 0.336366304545304 | -4.44856954252231 | 8.64440617526913e-06 | 0.000336589378099801 | "-" |  |
| **MSTRG.19299** | **59.3038342346833** | **1.49447083007786** | **0.443287394304864** | **3.37133617891705** | **0.000748045135630826** | **0.00960523910805002** | **Asic3** |  |
| **MSTRG.18944** | **42.5660501957819** | **1.4900917924899** | **0.357945511771296** | **4.16290117765736** | **3.14229386576423e-05** | **0.000915788219817803** | **Gm9768** |  |
| MSTRG.22348 | 102.655286809971 | -1.48895567712201 | 0.289504833873534 | -5.14311162684227 | 2.70224949941811e-07 | 2.27737728162687e-05 | Ninj2 |  |
| **ENSMUSG00000001815** | **25.657655082518** | **1.48846380086493** | **0.431884564078444** | **3.44643899010611** | **0.000568026807870179** | **0.00788311374414351** | **Evx2** |  |
| **MSTRG.21992** | **276.311452228491** | **1.4831206672127** | **0.251178762487689** | **5.90464198694106** | **3.53413379811343e-09** | **8.78932981745779e-07** | **1810020O05Rik** |  |
| **MSTRG.25146** | **37.0607719297758** | **1.48260117979034** | **0.457450596837778** | **3.24100829693769** | **0.0011910771716706** | **0.0134005304240709** | **"-"** |  |
| **MSTRG.26178** | **30.2580290580778** | **1.48147392632702** | **0.51143494226872** | **2.89670064339996** | **0.00377109409763733** | **0.0307177669626177** | **"-"** |  |
| **MSTRG.25907** | **8488.84501828937** | **1.47946861095188** | **0.382688950040162** | **3.86598204833615** | **0.000110643076683607** | **0.00239394778403732** | **Mt2** |  |
| **MSTRG.8510** | **2497.49707834627** | **1.47891257765476** | **0.194718453433704** | **7.59513313492026** | **3.07476145233996e-14** | **5.9143036535759e-11** | **"-"** |  |
| MSTRG.19345 | 169.158017749947 | -1.47702211512107 | 0.305871800819229 | -4.82889272945428 | 1.37294339060784e-06 | 8.17259204084464e-05 | "-" |  |
| **MSTRG.22313** | **105.817918531309** | **1.47696054437358** | **0.371405598270841** | **3.97667819561656** | **6.98846351107669e-05** | **0.00170155817260203** | **Depp1** |  |
| **MSTRG.15034** | **25.6118611846922** | **1.47584367915268** | **0.470910347656676** | **3.13402261491324** | **0.00172427566482073** | **0.0174330712288162** | **"-"** |  |
| MSTRG.10042 | 26.0534281601937 | -1.47332170427085 | 0.503727998599944 | -2.92483584070329 | 0.00344637975278718 | 0.0288222237151571 | Gm49566 |  |
| **MSTRG.5827** | **488.960977721482** | **1.47122376763042** | **0.221543627139696** | **6.6407857749062** | **3.12015249895433e-11** | **2.14343333276381e-08** | **Rhoj** |  |
| **MSTRG.10049** | **47.0811240117352** | **1.47091329111393** | **0.384741165966646** | **3.82312427477917** | **0.000131771322454713** | **0.00272833303274101** | **Gm49745** |  |
| MSTRG.24722 | 86.7666804339033 | -1.4668354541647 | 0.508492458267683 | -2.88467494515429 | 0.00391817990937537 | 0.0314812826051943 | Gm45426 |  |
| **ENSMUSG00000114664** | **20.0540910115307** | **1.46633452018073** | **0.537879385849** | **2.72614002090123** | **0.00640798081490896** | **0.044120437716771** | **Gm48639** |  |
| ENSMUSG00000031004 | 30.7691546128866 | -1.46571276259417 | 0.392598233797065 | -3.73336565582157 | 0.000188937990845473 | 0.00351812415674025 | Mki67 |  |
| MSTRG.17253 | 293.50046745449 | -1.46300890463907 | 0.31717604237044 | -4.61260848614277 | 3.9764687947688e-06 | 0.000186339294304278 | Slc9b2 |  |
| **MSTRG.7935** | **745.751690668966** | **1.46220975600983** | **0.218852503782823** | **6.6812566945126** | **2.36901841853806e-11** | **1.6877062696511e-08** | **Galnt15** |  |
| MSTRG.25079 | 46.0494524352051 | -1.46006111583264 | 0.401098642362129 | -3.64015471913375 | 0.000272474265851484 | 0.00452203839832035 | Tmem255b |  |
| **MSTRG.21429** | **99.2243845664808** | **1.4576712350055** | **0.360694885533937** | **4.04128612150324** | **5.31588510069735e-05** | **0.00136516755556627** | **Klrg2** |  |
| **MSTRG.21330** | **40.645476128378** | **1.45662635548205** | **0.508317844554512** | **2.86558178330063** | **0.00416243740819149** | **0.0327194456667607** | **"-"** |  |
| **MSTRG.25227** | **66.5538293009853** | **1.45032930344413** | **0.355740966237588** | **4.07692518177819** | **4.56351687775658e-05** | **0.00121074823646411** | **"-"** |  |
| MSTRG.7673 | 46.1138840221875 | -1.44925410867051 | 0.402810061793061 | -3.59785975111774 | 0.000320846539617738 | 0.00513007746429526 | "-" |  |
| MSTRG.5431 | 13.0848543179878 | -1.44772587525901 | 0.541594218424614 | -2.67308221913843 | 0.00751578273285764 | 0.0487246649364734 | Gm10478 |  |
| MSTRG.21609 | 30.8961838697633 | -1.44450580767191 | 0.498392450969694 | -2.89833003060423 | 0.00375155590442586 | 0.0306155525387072 | Gm8129 |  |
| **MSTRG.1821** | **291.457233235822** | **1.43754568035297** | **0.195023472155415** | **7.37114186546419** | **1.69172699006786e-13** | **2.32431204671109e-10** | **Dnah14** |  |
| **MSTRG.11890** | **80.8008422620289** | **1.43668983567495** | **0.323067534808802** | **4.44702633622785** | **8.70671668305081e-06** | **0.00033811470233936** | **"-"** |  |
| MSTRG.10673 | 58.3802050912788 | -1.43394663417273 | 0.389416962099495 | -3.68229115250086 | 0.000231147208994857 | 0.00405297772562996 | "-" |  |
| **MSTRG.3487** | **948.094869788413** | **1.42938371017464** | **0.154876698089729** | **9.22917215956212** | **2.72731536190679e-20** | **2.62299554931385e-16** | **"-"** |  |
| **MSTRG.21923** | **22.7900370261316** | **1.42907924792466** | **0.482848118600867** | **2.95968689298336** | **0.00307951849629931** | **0.02652688682325** | **Gm42600** |  |
| MSTRG.14101 | 36.1192951147359 | -1.4278747484204 | 0.379068733493807 | -3.76679642042734 | 0.000165355729136117 | 0.00321274489892243 | Pkn3 |  |
| MSTRG.6373 | 174.054083583635 | -1.42783709327826 | 0.216350674671178 | -6.59964243443367 | 4.12150610937752e-11 | 2.55732806496376e-08 | Xrcc3 |  |
| ENSMUSG00000021697 | 37.0227487244101 | -1.42757377945752 | 0.530267088287684 | -2.69217873594113 | 0.00709868903552833 | 0.0468585424077433 | Depdc1b |  |
| **MSTRG.3298** | **44.553040823707** | **1.42728758267647** | **0.417408775327634** | **3.41940003910114** | **0.000627593865078615** | **0.0084536190439686** | **"-"** |  |
| **MSTRG.11345** | **49.5000972501154** | **1.42721435543912** | **0.35546672643102** | **4.01504346066011** | **5.94348417200626e-05** | **0.00149052044391839** | **Cfb** |  |
| **MSTRG.16601** | **22.6731133390503** | **1.42459395972983** | **1.42459395972983** | **3.32821878851997** | **0.000874031953672286** | **0.0106878605396608** | **Creb3l4** |  |
| **MSTRG.20836** | **546.824233753074** | **1.414862049033** | **0.264711214517743** | **5.34492674067712** | **9.04536301872513e-08** | **9.63451661999889e-06** | **Sh2b2** |  |
| MSTRG.6100 | 18.5086069164213 | -1.41256296382048 | 0.46437402771529 | -3.04186470283505 | 0.00235117549150091 | 0.0217218350523631 | "-" |  |
| MSTRG.15271 | 75.5893420183611 | -1.40110981754283 | 0.326491123383048 | -4.29141779728882 | 1.77535924610645e-05 | 0.000587763082596515 | "-" |  |
| MSTRG.23584 | 110.263870108076 | -1.39685887245199 | 0.276757634258421 | -5.04722796968152 | 4.48266358613989e-07 | 3.35502078129964e-05 | A230056P14Rik |  |
| **MSTRG.24881** | **42.4373994207213** | **1.39457694095757** | **0.457060601056036** | **3.0511860740904** | **0.00227939267060498** | **0.0212443871452086** | **"-"** |  |
| **MSTRG.1833** | **36.823123515397** | **1.3914831430246** | **0.423943185037521** | **3.28223967770929** | **0.00102986028084561** | **0.0121158180440766** | **"-"** |  |
| **MSTRG.3654** | **20.2842401391882** | **1.38759169225522** | **0.50745907873007** | **2.73439130447267** | **0.006249572540686** | **0.0434015714279495** | **"-"** |  |
| MSTRG.24117 | 507.89695285096 | -1.38738909775442 | 0.390031826227315 | -3.55711766184393 | 0.000374946243380599 | 0.00579284416981994 | "-" |  |
| **MSTRG.18535** | **40.7677287043403** | **1.38696899321133** | **0.371549975056195** | **3.73292716007196** | **0.000189267341531192** | **0.00352084846649177** | **"-"** |  |
| **MSTRG.26041** | **95.0814125658213** | **1.38578306005864** | **0.307239577052776** | **4.51043147940733** | **6.46958968571868e-06** | **0.000269250982542808** | **Lrrc29** |  |
| MSTRG.8103 | 28.1055988804371 | -1.38479085022078 | 0.478329758147978 | -2.89505477472796 | 0.00379092376603243 | 0.030780252697186 | Gm49096 |  |
| MSTRG.22765 | 66.8021016381639 | -1.38335719402743 | 0.303666292245943 | -4.55551778169385 | 5.22566945369287e-06 | 0.000229759572892647 | Cox6b2 |  |
| MSTRG.16745 | 111.726906533225 | -1.37665615134475 | 0.423438196948114 | -3.25113832730927 | 0.00114943917857711 | 0.0130825222484797 | H2ac18 |  |
| MSTRG.2135 | 646.157123599798 | -1.37523504093719 | 0.180323787153146 | -7.62647603318816 | 2.41257755956669e-14 | 5.45964158733892e-11 | Enpp1 |  |
| MSTRG.20159 | 98.7911433477499 | -1.37517734720112 | 0.256378073726919 | -5.36386488598824 | 8.14598472853478e-08 | 8.75352046108192e-06 | "-" |  |
| **MSTRG.19610** | **42.1909922831918** | **1.37481712038269** | **0.342767340001159** | **4.01093383161314** | **6.04790598312635e-05** | **0.00151079833227838** | **Gm42462** |  |
| MSTRG.1339 | 277.994711070029 | -1.37440834307398 | 0.452768971673416 | -3.03556212784242 | 0.00240087754363211 | 0.0220328623815666 | Pla2g4a |  |
| **MSTRG.23927** | **81.5513856096869** | **1.37288229334514** | **0.240865432002349** | **5.69978963744266** | **1.19955357607189e-08** | **2.09758300324934e-06** | **Ttll13** |  |
| **MSTRG.2360** | **42.3239025430022** | **1.36963560367943** | **0.435163247017063** | **3.14740643440811** | **0.0016472581626322** | **0.0168600053448655** | **Gm47017** |  |
| **MSTRG.14227** | **118.63585076163** | **1.36794020841428** | **0.308209295756123** | **4.43834831476559** | **9.06518354625981e-06** | **0.000346851115706769** | **Gm38299** |  |
| **MSTRG.16715** | **87.1015004657478** | **1.36366846502162** | **0.272389471782286** | **5.00631854858022** | **5.54809125427353e-07** | **3.90906722622532e-05** | **C920021L13Rik** |  |
| **MSTRG.12587** | **38.3163120118292** | **1.35850719656182** | **0.466620998113885** | **2.91137176006438** | **0.003598455881549** | **0.0297576521417003** | **Cfap53** |  |
| **MSTRG.25734** | **84.385440419809** | **1.35751880696014** | **0.339167798535681** | **4.00249909578997** | **6.26769074729887e-05** | **0.00155761022641206** | **"-"** |  |
| **MSTRG.23098** | **338.748783269168** | **1.35475365984166** | **0.463097366620591** | **2.9254186214183** | **0.00343993133317842** | **0.0287808086966885** | **Zfp36** |  |
| MSTRG.10379 | 321.11013163843 | -1.35467332981734 | 0.215662951531748 | -6.28143740125859 | 3.35456871082495e-10 | 1.26519861083761e-07 | "-" |  |
| **MSTRG.26512** | **17.4174289573151** | **1.35395638177828** | **0.469032922859775** | **2.88669796039684** | **0.00389307756817457** | **0.0313569926357984** | **"-"** |  |
| **MSTRG.15078** | **98.8219062344035** | **1.35251325089** | **0.344706193878064** | **3.92366970745073** | **8.72102811582963e-05** | **0.00203085927128309** | **Hdc** |  |
| MSTRG.21931 | 71.7274036782938 | -1.35014763104064 | 0.299363848848675 | -4.51005569387614 | 6.48106082231974e-06 | 0.000269250982542808 | Nat8f6 |  |
| **MSTRG.13984** | **24001.8349731017** | **1.34954986994712** | **0.306579804364787** | **4.40195293601711** | **1.07280831496053e-05** | **0.000398368106916329** | **"-"** |  |
| **MSTRG.3913** | **229.115914584741** | **1.34465489094296** | **0.277022748086712** | **4.8539511654908** | **1.21025551149056e-06** | **7.34361664464382e-05** | **"-"** |  |
| MSTRG.12405 | 905.278040616679 | -1.34310351702574 | 0.406211638397844 | -3.30641318481946 | 0.000944986283797097 | 0.0113812576176419 | 9330117O12Rik |  |
| **MSTRG.28198** | **26.7033643318496** | **1.3413507428542** | **0.401134237180885** | **3.34389493223272** | **0.000826110178435243** | **0.0102982691394698** | **"-"** |  |
| **MSTRG.17949** | **32.5879568151745** | **1.33955882533349** | **0.420115040880612** | **3.18855240822993** | **0.00142987087904235** | **0.015248334337607** | **4933430I17Rik** |  |
| MSTRG.6651 | 162.295173738468 | -1.33951527682164 | 0.319691070022658 | -4.19003032123074 | 2.78917180999232e-05 | 0.000831778601010889 | H2bc6 |  |
| **MSTRG.12066** | **27.6350685903543** | **1.33675358099961** | **0.412357615992123** | **3.24173370190681** | **0.00118804979652698** | **0.0133873098044502** | **Gm50083** |  |
| MSTRG.16922 | 175.982801265173 | -1.33512732938549 | 0.324802551900248 | -4.11058140268407 | 3.94664112104946e-05 | 0.00109701794744778 | Inka2 |  |
| **MSTRG.14393** | **33.5862355501804** | **1.3349581884904** | **0.3650082314879** | **3.65733721414625** | **0.000254849008283663** | **0.00433040695612744** | **A930012O16Rik** |  |
| **MSTRG.17859** | **70.1183299157083** | **1.33490908756528** | **0.358522952027558** | **3.72335740296668** | **0.000196590916079318** | **0.00359346251218477** | **"-"** |  |
| MSTRG.20670 | 434.878312642004 | -1.33384356433587 | 0.424180392233706 | -3.14451961655262 | 0.00166359757529949 | 0.0169807827105834 | "-" |  |
| **ENSMUSG00000036832** | **29.9990533426368** | **1.33077837466107** | **0.435255040855317** | **3.05746803539819** | **0.00223215446600615** | **0.0209645952898576** | **Lpar3** |  |
| **MSTRG.13608** | **151.568107980385** | **1.33053833220957** | **0.359761242300037** | **3.69839264425242** | **0.000216969081043289** | **0.00386068480468794** | **Mcm10** |  |
| **MSTRG.10165** | **45835.4228181835** | **1.32260766172579** | **0.375497951487575** | **3.52227663689281** | **0.000427857419534348** | **0.00639459010469555** | **Apod** |  |
| **ENSMUSG00000113362** | **40.2910723564666** | **1.31982610617354** | **0.381591746482211** | **3.45873860831805** | **0.000542711322616898** | **0.00760337790251277** | **Gm40557** |  |
| MSTRG.24707 | 33.4851116989107 | -1.31778575192119 | 0.421591237587704 | -3.12574274423114 | 0.00177356627542771 | 0.0177425404576764 | "-" |  |
| **MSTRG.2930** | **19.2108295006253** | **1.31719564329444** | **0.452554809201223** | **2.91057705390281** | **0.00360761999627042** | **0.0298078052526897** | **Pah** |  |
| MSTRG.1510 | 787.44170049139 | -1.31389661025859 | 0.474541005752049 | -2.76877360298154 | 0.00562677193680478 | 0.0404148462301867 | Myoc |  |
| **MSTRG.6161** | **549.665015692213** | **1.31179625671765** | **0.451854536859788** | **2.90313839899478** | **0.0036944333297986** | **0.0302727650538924** | **Fbln5** |  |
| **MSTRG.19517** | **157.951743567686** | **1.31091692342322** | **0.253777461346836** | **5.16561603408746** | **2.39648302031712e-07** | **2.09528867708181e-05** | **"-"** |  |
| **MSTRG.2142** | **504.735546963981** | **1.30934517228611** | **0.41006790464234** | **3.19299598301438** | **0.00140804905263683** | **0.0150778413355421** | **Ccn2** |  |
| MSTRG.26449 | 39.0009339826934 | -1.30547619071177 | 0.384497355883116 | -3.39528002140182 | 0.000685584420654107 | 0.00902615765317026 | "-" |  |
| **MSTRG.18648** | **47.1556703689296** | **1.3053714496782** | **0.420280408238185** | **3.10595360642747** | **0.00189666470581179** | **0.0186039498298265** | **Crybg2** |  |
| **MSTRG.16312** | **92.6618755891326** | **1.30360127354661** | **0.259642593828573** | **5.0207527752835** | **5.14693695687503e-07** | **3.73696150779363e-05** | **Vmn2r1** |  |
| MSTRG.6134 | 585.994991665571 | -1.30055640230823 | 0.245992965011185 | -5.28696583761695 | 1.24361853605415e-07 | 1.23304136809286e-05 | Kcnk13 |  |
| **ENSMUSG00000105107** | **23.6303799452606** | **1.29341167115098** | **0.413818601611704** | **3.12555227366172** | **0.00177471525035453** | **0.0177425404576764** | **Gm43412** |  |
| MSTRG.8696 | 158.082761319132 | -1.2925239190029 | 0.255687071458329 | -5.0551007981393 | 4.30163513898815e-07 | 3.27043288136114e-05 | 9330188P03Rik |  |
| **MSTRG.18805** | **27.4206737075177** | **1.28887449375757** | **0.42805246141295** | **3.01101993317163** | **0.00260371759525773** | **0.0233376085483609** | **"-"** |  |
| **MSTRG.13981** | **44.0983765918531** | **1.28573671831762** | **0.434652218569525** | **2.95808157277808** | **0.00309560193433011** | **0.0266296525969766** | **Fut7** |  |
| **MSTRG.10990** | **78.7871782001901** | **1.28387391944921** | **0.330686511841005** | **3.8824502163744** | **0.000103409191837111** | **0.00226288487484281** | **Tekt4** |  |
| **MSTRG.5679** | **1257.49669926939** | **1.27890913394427** | **0.177061976356375** | **7.2229462262987** | **5.08731350351674e-13** | **6.11590470250903e-10** | **Nfkbia** |  |
| MSTRG.17627 | 892.448026406926 | -1.27723913412294 | 0.429034605815073 | -2.97700725491935 | 0.00291077122087733 | 0.0254798836342834 | Mob3b |  |
| **MSTRG.27197** | **156.96454436904** | **1.2709002380503** | **0.290978515903192** | **4.36767722904028** | **1.25574887796025e-05** | **0.00044730240125121** | **"-"** |  |
| MSTRG.24611 | 40.9565706664861 | -1.26942414784234 | 0.397755967027468 | -3.19146474992965 | 0.00141553382658233 | 0.0151297357061538 | "-" |  |
| MSTRG.17913 | 2915.76860326801 | -1.26914885128483 | 0.270646498477255 | -4.6893230040864 | 2.74110413080055e-06 | 0.00014060036788253 | Lpar1 |  |
| **ENSMUSG00000028784** | **58.8772807765057** | **1.26822499277733** | **0.312875520941025** | **4.0534490808451** | **5.04679887098705e-05** | **0.00131037794632185** | **Spocd1** |  |
| MSTRG.16047 | 2678.03480419143 | -1.2650434915203 | 0.3033633040537 | -4.17006102787028 | 3.04518081937894e-05 | 0.00089289715031637 | Sox2ot |  |
| MSTRG.14819 | 125.050857711595 | -1.26466944919579 | 0.350638756902775 | -3.60675887733214 | 0.000310045522124139 | 0.00500312551850488 | Depdc7 |  |
| **MSTRG.27365** | **44.8159384875036** | **1.26318356625566** | **0.408782025014168** | **3.09011524225381** | **0.00200078847209279** | **0.0193490026448993** | **Myzap** |  |
| MSTRG.7967 | 23.9610356287995 | -1.2621467460979 | 0.441138104678777 | -2.86111476816758 | 0.0042215420029922 | 0.0329818685733367 | Gm15512 |  |
| **MSTRG.25196** | **62.5847869860402** | **1.26065563610711** | **0.2910580719127** | **4.33128560160747** | **1.4824124889574e-05** | **0.000511924671904769** | **Tcim** |  |
| **MSTRG.26763** | **38.4777582060234** | **1.2596876833971** | **0.399168004928685** | **3.15578319866131** | **0.00160067790424854** | **0.0165323438139037** | **"-"** |  |
| MSTRG.14854 | 34.9211510503606 | -1.25872332831813 | 0.46296403272879 | -2.71883610676838 | 0.00655120597483335 | 0.0447011163270378 | "-" |  |
| MSTRG.6271 | 3380.79029558795 | -1.25774390141912 | 0.184656650292212 | -6.81125699739917 | 9.67496146594971e-12 | 8.45899471807012e-09 | Eml1 |  |
| MSTRG.8371 | 88.9149222456601 | -1.25456939900851 | 0.381734924287069 | -3.28649363521443 | 0.00101443066350869 | 0.0119562339537926 | Dleu2 |  |
| MSTRG.11931 | 68.6146944718989 | -1.25263899279152 | 0.335403215213961 | -3.73472565548436 | 0.000187919928767731 | 0.00350595521808663 | Gm20939 |  |
| MSTRG.22942 | 18.5671907347343 | -1.24982195085583 | 0.460889533132736 | -2.711760326516 | 0.00669269649768789 | 0.0452810471801008 | Zfp296 |  |
| **MSTRG.26768** | **30.6615152116108** | **1.24932984363456** | **0.376570515556115** | **3.31765178638473** | **0.000907775774819276** | **0.0110164460748573** | **4930517E14Rik** |  |
| MSTRG.11238 | 103.660908910166 | -1.24607669172872 | 0.308265491524067 | -4.042219210357 | 5.29476980876357e-05 | 0.00136471337524039 | Myo1f |  |
| **MSTRG.10834** | **42.6454839325755** | **1.24516847111259** | **0.388856401945308** | **3.20212928187235** | **0.00136415760250747** | **0.0147165291554857** | **Dynlt2a2** |  |
| MSTRG.24989 | 20.3281027594194 | -1.23825343963219 | 0.465302879051022 | -2.66117725761247 | 0.00778679518158188 | 0.0497770040936282 | Rps23-ps2 |  |
| **MSTRG.11052** | **441.74145092567** | **1.23644161600531** | **0.191047237567822** | **6.47191569868354** | **9.67681094950274e-11** | **5.13308875461362e-08** | **"-"** |  |
| ENSMUSG00000060284 | 74.5217160050519 | -1.23635385010752 | 0.378615198714429 | -3.26546280842793 | 0.00109285355875175 | 0.0126379618091709 | Sp7 |  |
| **MSTRG.1785** | **33.5355438598998** | **1.23550656747577** | **0.394466393890091** | **3.13209588094853** | **0.00173563183799027** | **0.0175156759725828** | **"-"** |  |
| **MSTRG.21472** | **17.1630304480116** | **1.22634471712757** | **0.45941562018523** | **2.6693579043593** | **0.00759964217136114** | **0.0491193270047485** | **"-"** |  |
| **MSTRG.13941** | **1998.45514716758** | **1.22618277067984** | **0.443084512157372** | **2.76737899212403** | **0.00565090195642812** | **0.0405578728104086** | **Mrpl41** |  |
| **MSTRG.9491** | **218.873483142234** | **1.22270112952266** | **0.306721655203394** | **3.98635410568535** | **6.7096352836553e-05** | **0.00164617136072844** | **"-"** |  |
| **MSTRG.16753** | **1678.66764307106** | **1.22253609925296** | **0.317947405815017** | **3.84508908358334** | **0.000120508650720838** | **0.00253055010547524** | **Txnip** |  |
| MSTRG.13183 | 3983.12199400093 | -1.22156779957939 | 0.349717681087537 | -3.49301126491693 | 0.000477606360884824 | 0.00689696572944414 | Il33 |  |
| MSTRG.5125 | 31.8862284180228 | -1.21981639141042 | 0.369649535873488 | -3.29992674961157 | 0.000967100672958302 | 0.011568520798727 | Tmem235 |  |
| **MSTRG.12972** | **42.7891585207913** | **1.21885430143868** | **0.297219410631486** | **4.100857002741** | **4.11622873495697e-05** | **0.00112625405002699** | **Gm9750** |  |
| **MSTRG.2969** | **69.1117032140405** | **1.21724912019167** | **0.353686630307109** | **3.44160343051341** | **0.000578277347179685** | **0.00797359481935573** | **"-"** |  |
| MSTRG.6959 | 42.8677438260888 | -1.21564875820594 | 0.396104269339132 | -3.06901200594014 | 0.00214767960110632 | 0.0204406814088471 | Hk3 |  |
| **MSTRG.24896** | **206.148964142704** | **1.21352528398781** | **0.236221166229215** | **5.13724194727868** | **2.78799916053429e-07** | **2.33161581969031e-05** | **Gm20501** |  |
| **MSTRG.11294** | **207.513115079514** | **1.21132472169129** | **0.176744994231621** | **6.85351642889454** | **7.20563721908812e-12** | **6.60002056710286e-09** | **BC051142** |  |
| **MSTRG.4334** | **61.135723990591** | **1.21056564481846** | **0.390021350804528** | **3.10384455189782** | **0.00191023666905573** | **0.0187179838661676** | **Abhd15** |  |
| **MSTRG.14975** | **453.690634636682** | **1.2101577314547** | **0.168927383348583** | **7.16377479758586** | **7.84852990928639e-13** | **8.88038075324257e-10** | **Pla2g4e** |  |
| MSTRG.984 | 67.1392390209248 | -1.20955084386969 | 0.310435266948497 | -3.89630616314725 | 9.76709085188245e-05 | 0.00216689726108373 | "-" |  |
| MSTRG.3269 | 30.2783143426716 | -1.20713957955637 | 0.436838332608935 | -2.76335543253944 | 0.00572104295491995 | 0.0408934452760629 | Myo1a |  |
| MSTRG.26165 | 3718.46285517298 | -1.20689341417043 | 0.291874527289177 | -4.13497342635418 | 3.54995710909917e-05 | 0.00101010983718229 | Fa2h |  |
| **MSTRG.23614** | **62.6104559769563** | **1.20656516549987** | **0.351681053210295** | **3.43085063720616** | **0.0006016917992546** | **0.00820236836191512** | **Gm27252** |  |
| MSTRG.17360 | 50.5452843093862 | -1.20488106714078 | 0.383179121273071 | -3.1444329825114 | 0.00166409022144266 | 0.0169807827105834 | Gm29771 |  |
| **MSTRG.2553** | **57.410989910296** | **1.19867604457903** | **0.328546258023358** | **3.64842397472628** | **0.000263853956373423** | **0.00444027196049238** | **"-"** |  |
| **MSTRG.17182** | **157.831881375536** | **1.19787535396067** | **0.233830344196463** | **5.12283963006196** | **3.00968316470085e-07** | **2.47398528517183e-05** | **Egf** |  |
| **MSTRG.16165** | **35.1021586787738** | **1.19582265453736** | **0.40509471165556** | **2.95195819676395** | **0.0031576567629859** | **0.0270425324292226** | **"-"** |  |
| **MSTRG.7653** | **180.21918178477** | **1.19170171738132** | **0.259832094429469** | **4.58643001742346** | **4.50889443730103e-06** | **0.000203588226529308** | **"-"** |  |
| MSTRG.12853 | 788.862917665599 | -1.18902810349975 | 0.211720312489943 | -5.61603225272131 | 1.95392204523085e-08 | 2.91346438294693e-06 | Cdc42ep2 |  |
| MSTRG.11567 | 40.6308379218345 | -1.18789339770491 | 0.395253416246407 | -3.00539691468311 | 0.00265234485079059 | 0.0236961696260832 | Gm37176 |  |
| MSTRG.23355 | 2735.87687071611 | -1.18743877558928 | 0.328383572998006 | -3.61601149761681 | 0.000299177092947435 | 0.00486036434361817 | "-" |  |
| MSTRG.9320 | 251.924693383259 | -1.18726263600752 | 0.337590723678268 | -3.51686984485691 | 0.000436667845997555 | 0.0064759491270339 | "-" |  |
| **MSTRG.4114** | **2892.30408539777** | **1.1872111660644** | **0.198629906042632** | **5.97700109574431** | **2.27282670831942e-09** | **6.52504802007823e-07** | **"-"** |  |
| MSTRG.13301 | 93.9130109248569 | -1.18433243198955 | 0.313231195727754 | -3.78101685956885 | 0.000156189095218891 | 0.00308133050926705 | Tll2 |  |
| **MSTRG.28857** | **111.133968233296** | **1.18379028165137** | **0.213136135660634** | **5.5541510029827** | **2.78964255946647e-08** | **3.7523618623313e-06** | **"-"** |  |
| **MSTRG.26664** | **42.306074706129** | **1.18307879601842** | **0.40705194553184** | **2.90645655672435** | **0.00365547654798704** | **0.0300611763148913** | **Acta1** |  |
| MSTRG.16695 | 142.506843571468 | -1.18162485498119 | 0.210318159007014 | -5.6182730990042 | 1.92875470098209e-08 | 2.89840599010863e-06 | Ctsk |  |
| **MSTRG.4349** | **31.6106447669731** | **1.18140085680037** | **0.434712302503838** | **2.71766142801983** | **0.00657450754663661** | **0.0448283065081727** | **Dhrs13os** |  |
| **MSTRG.17194** | **5694.08258648951** | **1.18061498033169** | **0.260656219148648** | **4.52939501765124** | **5.91528166588027e-06** | **0.000251704543793162** | **"-"** |  |
| **MSTRG.11563** | **96.5780559648679** | **1.180457515723** | **0.296391713749794** | **3.98276153131431** | **6.81190981163956e-05** | **0.00166701126242859** | **Gm7334** |  |
| **MSTRG.12521** | **78.7390570784946** | **1.17875113035335** | **0.312030822374716** | **3.7776752994542** | **0.000158299077416361** | **0.0031070232184731** | **A330084C13Rik** |  |
| MSTRG.24213 | 76.6845806310841 | -1.17779246585397 | 0.285154502088564 | -4.13036601992056 | 3.62186125441125e-05 | 0.00102601621838881 | Coa4 |  |
| **MSTRG.2670** | **760.608837005682** | **1.17411942010918** | **0.387357313914194** | **3.03110171909462** | **0.00243663146154603** | **0.0222442364322914** | **Icosl** |  |
| MSTRG.4743 | 519.305772961464 | -1.17321570718959 | 0.229519075578269 | -5.11162614363835 | 3.19397438770807e-07 | 2.58134862804894e-05 | Igfbp4 |  |
| MSTRG.8288 | 89.1631800091887 | -1.17310862018999 | 0.308544991502388 | -3.80206664343444 | 0.000143494114187088 | 0.00290511295616451 | Cideb |  |
| **MSTRG.14961** | **97.3671053171398** | **1.1727719751897** | **0.311748404539436** | **3.7619181304947** | **0.000168615245637171** | **0.00325307347024172** | **"-"** |  |
| **MSTRG.19783** | **107.789741729447** | **1.17208884815277** | **0.251212046654672** | **4.66573503843143** | **3.0751575670086e-06** | **0.000152740450880874** | **Gm20033** |  |
| **MSTRG.1155** | **66.7481144615253** | **1.16912753327222** | **0.319983377284529** | **3.65371333721699** | **0.000258474836001626** | **0.00438040834404518** | **Ctse** |  |
| MSTRG.21360 | 90.3167576997424 | -1.16647254769077 | 0.422662957288167 | -2.75981731442692 | 0.00578336920662135 | 0.0411706538450635 | "-" |  |
| **MSTRG.28921** | **589.349354468438** | **1.16554858544707** | **0.254965442585406** | **4.5713982790301** | **4.84480456422304e-06** | **0.00021621767005297** | **"-"** |  |
| MSTRG.28303 | 238.844176133814 | -1.16468156361113 | 0.345885088572877 | -3.36724999743877 | 0.000759218291247365 | 0.00967051440859314 | Gpr34 |  |
| **MSTRG.12133** | **39.0931076978917** | **1.16403474437833** | **0.387394474733553** | **3.00477890186467** | **0.00265773968196896** | **0.0237223307576209** | **"-"** |  |
| **MSTRG.26751** | **29.079253586825** | **1.16394359316587** | **0.413026865612271** | **2.8180820427757** | **0.00483114635355326** | **0.0364277146650713** | **Gm48796** |  |
| **MSTRG.321** | **19.0009966712105** | **1.16293032726979** | **0.42334096543149** | **2.74702998819042** | **0.00601376355665318** | **0.0423525523389773** | **Gm37062** |  |
| MSTRG.17747 | 55.60732179587 | -1.16017378777264 | 0.418743496073938 | -2.77060730172581 | 0.00559518614608124 | 0.0402933004567101 | Atp8b5 |  |
| MSTRG.27738 | 35865.4666948709 | -1.15995133812209 | 0.193750815941422 | -5.98682040375403 | 2.13982835285998e-09 | 6.23630278291844e-07 | Trf |  |
| MSTRG.23905 | 188.978575836986 | -1.15456534690345 | 0.334680519424878 | -3.44975366025927 | 0.000561098310950861 | 0.00779821243579467 | Rlbp1 |  |
| **MSTRG.3460** | **133.322125584834** | **1.15289374278239** | **0.253979715158754** | **4.53931426004534** | **5.64374696429527e-06** | **0.000242857881114585** | **Snhg15** |  |
| **MSTRG.13974** | **87.9191473776063** | **1.15211572912326** | **0.28243253709892** | **4.07925992152858** | **4.51793005675937e-05** | **0.00120363413631256** | **Grin1os** |  |
| **MSTRG.841** | **58.3321565403494** | **1.14967812753831** | **0.323544811491713** | **3.55338143806938** | **0.000380312585091182** | **0.00585225005938311** | **"-"** |  |
| MSTRG.12850 | 1677.48362702227 | -1.14789090777753 | 0.180143501185865 | -6.37209169479379 | 1.86467338706077e-10 | 8.53976014288428e-08 | Frmd8 |  |
| **ENSMUSG00000037638** | **68.816901084705** | **1.14778314438552** | **0.421648441047573** | **2.72213302042311** | **0.00648620284177065** | **0.0443993279934016** | **Zbtb42** |  |
| MSTRG.16852 | 433.373222969216 | -1.14478900010455 | 0.259247720985128 | -4.41581123935984 | 1.00631871169357e-05 | 0.000375855153775258 | "-" |  |
| MSTRG.9568 | 28.2142283835712 | -1.14468871699976 | 0.407112798651518 | -2.81172373060077 | 0.00492768075334496 | 0.0369528028423354 | "-" |  |
| **ENSMUSG00000070337** | **148.353957424672** | **1.14460186255303** | **0.416762363278785** | **2.7464136961604** | **0.00602507357868011** | **0.0423892795486144** | **Gpr179** |  |
| MSTRG.15911 | 10623.1227209113 | -1.14060511623474 | 0.290728724193995 | -3.92326255135922 | 8.7357860718359e-05 | 0.0020318360954264 | "-" |  |
| **MSTRG.24586** | **1427.15017111663** | **1.13908104775218** | **0.206285837343175** | **5.52185774080657** | **3.35434028756728e-08** | **4.4497058918177e-06** | **Sult1a1** |  |
| **MSTRG.4161** | **492.714088301296** | **1.13494572418999** | **0.231279227534976** | **4.90725317741023** | **9.23607762936256e-07** | **5.86323277890392e-05** | **"-"** |  |
| **MSTRG.18108** | **37.0293135656432** | **1.13481558689049** | **0.407036930363347** | **2.78799170846115** | **0.00530359012017068** | **0.0389071533033879** | **Gm12689** |  |
| **MSTRG.28137** | **148.327647211236** | **1.13462095162568** | **0.273655226967786** | **4.14616948558869** | **3.38083613137457e-05** | **0.000968946581876604** | **Slc6a20a** |  |
| **MSTRG.25908** | **9871.16164901637** | **1.13452480144855** | **0.319681536538151** | **3.54892188561898** | **0.000386811809826806** | **0.00591440791893371** | **Mt1** |  |
| **MSTRG.16324** | **64.9563816443311** | **1.13289344468832** | **0.424609878506386** | **2.66808075373423** | **0.00762859200438251** | **0.0492418339201278** | **4931440P22Rik** |  |
| MSTRG.2749 | 300.018248849994 | -1.13257658786751 | 0.223646031148823 | -5.06414793971391 | 4.10230993629167e-07 | 3.16899323793455e-05 | Gamt |  |
| MSTRG.13563 | 1461.87519889721 | -1.13201526265154 | 0.290611379846838 | -3.8952888329705 | 9.80817808209127e-05 | 0.00217350582268463 | Zfp950 |  |
| **MSTRG.4943** | **185.176790028793** | **1.13153969087227** | **0.302533235565406** | **3.74021614107135** | **0.000183862081680289** | **0.00345769718750212** | **"-"** |  |
| **MSTRG.3393** | **349.775674159398** | **1.13030520725529** | **0.236575624941036** | **4.7777754260905** | **1.77245176551563e-06** | **9.8145250388199e-05** | **Castor1** |  |
| MSTRG.18066 | 82.7359846958341 | -1.12922375635353 | 0.298284728307453 | -3.78572434050192 | 0.000153261496309357 | 0.00303771626963797 | "-" |  |
| **MSTRG.24514** | **38.1265533356708** | **1.12813591693739** | **0.400436741247664** | **2.81726375412604** | **0.00484347325158302** | **0.036454610220246** | **Chp2** |  |
| **MSTRG.15132** | **51.0202653566565** | **1.12760753097302** | **0.388265129689757** | **2.90422045336402** | **0.00368168820777699** | **0.030212147046327** | **"-"** |  |
| MSTRG.28885 | 82.7278973583928 | -1.12469992413338 | 0.315621448758389 | -3.56344579418727 | 0.000366018304495243 | 0.00568688375360743 | BC065397 |  |
| **MSTRG.3371** | **1188.62541358472** | **1.12298181726042** | **0.213315578964073** | **5.26441539204013** | **1.40636146944088e-07** | **1.34625780977116e-05** | **8430429K09Rik** |  |
| **MSTRG.2729** | **171.318341658889** | **1.12266408762915** | **0.217132317977764** | **5.17041451076904** | **2.335753097192e-07** | **2.05151647600402e-05** | **"-"** |  |
| **MSTRG.11632** | **94.8481281917716** | **1.12206163589891** | **0.345426430495211** | **3.24833752382614** | **0.00116081482843039** | **0.0131768553503065** | **Nrtn** |  |
| **MSTRG.27467** | **196.670296262591** | **1.12033706054722** | **0.290468319676821** | **3.85700258738622** | **0.000114785947800275** | **0.00244215331193873** | **C920006O11Rik** |  |
| **MSTRG.12149** | **72.969188058536** | **1.11942797729066** | **0.283356158080811** | **3.95060402029946** | **7.79542079228112e-05** | **0.0018511718387596** | **"-"** |  |
| ENSMUSG00000074221 | 105.442738166702 | -1.11881120498955 | 0.323649295812598 | -3.45686278161216 | 0.000546503051867564 | 0.00763952485659346 | Zfp568 |  |
| **MSTRG.13341** | **46.027761031434** | **1.11864677698336** | **0.363135464134813** | **3.08052197448847** | **0.0020663810609108** | **0.019863488109255** | **Gm16244** |  |
| **MSTRG.25485** | **877.128235342821** | **1.11794713440891** | **0.199795403692559** | **5.59545972403443** | **2.20037926458106e-08** | **3.1872176709379e-06** | **Tma16** |  |
| **MSTRG.24918** | **91.1260582256893** | **1.11723781164295** | **0.296889373304513** | **3.76314517157548** | **0.000167789733914449** | **0.00324365380084866** | **Gm33148** |  |
| ENSMUSG00000043020 | 27.5603484461644 | -1.1170630005167 | 0.377914467736933 | -2.95586196317387 | 0.00311796600815237 | 0.0267860992259092 | Dnai3 |  |
| **MSTRG.18055** | **78.2735377806934** | **1.11578962598924** | **0.395622511879302** | **2.82033906687709** | **0.0047972929852501** | **0.036257733033904** | **Dmrta1** |  |
| **MSTRG.25808** | **105.625735172584** | **1.11486416829543** | **0.296698030705398** | **3.7575718505608** | **0.000171570111805645** | **0.00329356397263631** | **"-"** |  |
| ENSMUSG00000033491 | 73.9540664714693 | -1.11062269311176 | 0.366816852722624 | -3.02773082770976 | 0.00246397454302374 | 0.0224157391019008 | Prss35 |  |
| MSTRG.4639 | 53.4326696635518 | -1.10952955996641 | 0.360977052564681 | -3.07368446853723 | 0.00211432926481914 | 0.0201731762940457 | Gngt2 |  |
| MSTRG.21155 | 810.515108328261 | -1.10905575852315 | 0.167292350804757 | -6.62944691247418 | 3.36946987228743e-11 | 2.23488803425685e-08 | Gng11 |  |
| **MSTRG.246** | **31.6280687229456** | **1.1082601309217** | **0.336119788144897** | **3.29721774798914** | **0.000976477652206061** | **0.0116444808680617** | **Gm38336** |  |
| MSTRG.12612 | 91.4802088175293 | -1.10614304948154 | 0.356462160935671 | -3.10311491850369 | 0.00191495265411035 | 0.0187450963368003 | Katnal2 |  |
| MSTRG.11334 | 124.856420064228 | -1.1050159577044 | 0.195911717484374 | -5.6403770631664 | 1.69678179183913e-08 | 2.63438075003358e-06 | Aif1 |  |
| MSTRG.28710 | 2217.68963957728 | -1.10481749398248 | 0.197740874113357 | -5.58719839252421 | 2.30762050763878e-08 | 3.27367612441669e-06 | Gjb1 |  |
| MSTRG.13284 | 55.3692348092962 | -1.10143981207953 | 0.337329204948726 | -3.2651777430506 | 0.00109395406354403 | 0.0126379618091709 | Blnk |  |
| **MSTRG.13633** | **2979.89517140693** | **1.09845400165628** | **0.171090796319363** | **6.42029860920089** | **1.36007290925131e-10** | **6.70794933575613e-08** | **"-"** |  |
| MSTRG.15612 | 511.920663593109 | -1.09803157197232 | 0.175218444734279 | -6.26664375224595 | 3.68912822003353e-10 | 1.33887511910085e-07 | Slc13a3 |  |
| **MSTRG.21475** | **240.171567739569** | **1.09789388380794** | **0.244497551805385** | **4.49040849571307** | **7.10867092323457e-06** | **0.000288112338621785** | **Clcn1** |  |
| ENSMUSG00000110320 | 30.6998852198567 | -1.0964658417835 | 0.351629174589931 | -3.11824479030272 | 0.00181931628714233 | 0.0180198500428336 | Gm31152 |  |
| **MSTRG.11040** | **607.792970823635** | **1.09608355770663** | **0.141020504965673** | **7.77251193344851** | **7.69447533257151e-15** | **2.82184441894152e-11** | **Dusp1** |  |
| **ENSMUSG00000028392** | **37.5525149791022** | **1.09581411162014** | **0.393257176507584** | **2.78650760134063** | **0.00532793685029261** | **0.0390559700134826** | **Bspry** |  |
| **ENSMUSG00000103662** | **70.5863705776299** | **1.09457221361455** | **0.360486821470668** | **3.03637233990705** | **0.00239443478781098** | **0.0219842258441738** | **Gm34294** |  |
| **MSTRG.24785** | **84.5231075249732** | **1.09421774007155** | **0.267168517422201** | **4.09560883381474** | **4.2106036165883e-05** | **0.0011464764307339** | **"-"** |  |
| MSTRG.22472 | 704.865296296617 | -1.09417226945764 | 0.193672440194159 | -5.6496023304127 | 1.60819430090336e-08 | 2.5564973039567e-06 | "-" |  |
| **MSTRG.21067** | **251.90181067558** | **1.09411003709566** | **0.321316455905663** | **3.40508560015018** | **0.000661433170454241** | **0.00881071124216574** | **B230303O12Rik** |  |
| MSTRG.26874 | 241.849156999751 | -1.09251751379917 | 0.323749504554306 | -3.37457663542437 | 0.000739293284626925 | 0.00953103641407433 | Gm35657 |  |
| ENSMUSG00000029177 | 29.8804329338532 | -1.09117389461668 | 0.323303478973279 | -3.37507625368569 | 0.000737952404352563 | 0.00952651979712855 | Cenpa |  |
| **MSTRG.26431** | **842.776339014428** | **1.0901985978845** | **0.197958159622483** | **5.50721728249833** | **3.64550201140091e-08** | **4.70612289861051e-06** | **Col5a3** |  |
| MSTRG.16866 | 445.215021023715 | -1.0895416361252 | 0.218856545845734 | -4.97833698286178 | 6.41329262493031e-07 | 4.34365083241319e-05 | Olfml3 |  |
| **MSTRG.13985** | **1183.24345794626** | **1.08924074287681** | **0.211184564759049** | **5.15776682883797** | **2.49912563485536e-07** | **2.13016768552212e-05** | **"-"** |  |
| **MSTRG.15778** | **4070.43300157679** | **1.08877545791162** | **0.212703777881479** | **5.11874057318482** | **3.07582710237364e-07** | **2.51759720485774e-05** | **Phactr3** |  |
| MSTRG.10991 | 66.8477183690013 | -1.08642594972483 | 0.374105075992858 | -2.90406631570424 | 0.00368350129375471 | 0.0302141353455743 | "-" |  |
| MSTRG.21632 | 1193.39084486103 | -1.08512428086466 | 0.205372092671326 | -5.28369880615316 | 1.26601334444907e-07 | 1.24243707553458e-05 | Chn2 |  |
| **MSTRG.5167** | **258.802849369826** | **1.0839893470638** | **0.230280634031643** | **4.70725361523387** | **2.51076462211755e-06** | **0.000132677355786899** | **"-"** |  |
| MSTRG.14588 | 34.2749771009188 | -1.08307472416724 | 0.387798702252528 | -2.79287867101206 | 0.00522412824406561 | 0.0385151808258344 | Hoxd1 |  |
| MSTRG.27345 | 43.2939643281014 | -1.07964247210564 | 0.368178007699526 | -2.93239261859102 | 0.00336361219295494 | 0.0282652164838306 | Foxb1 |  |
| MSTRG.10504 | 1015.6832220557 | -1.079534063452 | 0.397252607309154 | -2.71750025950584 | 0.00657771038152871 | 0.0448342520158415 | Chodl |  |
| MSTRG.11325 | 90.6896659295004 | -1.07750079454324 | 0.33627572995045 | -3.20421814176719 | 0.00135429822426409 | 0.0146519676738126 | "-" |  |
| **MSTRG.23240** | **229.207699121183** | **1.07489128284306** | **0.309548190501166** | **3.47245216036567** | **0.000515726918860919** | **0.00730486545234888** | **Hpn** |  |
| **MSTRG.22881** | **131.435300257762** | **1.07482699914306** | **0.403779613361044** | **2.66191497435011** | **0.0077697502166511** | **0.0497176132459361** | **Slc1a5** |  |
| **MSTRG.25122** | **41.0359633994213** | **1.07216313055791** | **0.354412939676244** | **3.02518054656054** | **0.0024848474652584** | **0.0225453023557761** | **Angpt2** |  |
| MSTRG.2442 | 1867.18109927023 | -1.07092058826876 | 0.181371664922347 | -5.90456391701133 | 3.53580769799732e-09 | 8.78932981745779e-07 | Unc5b |  |
| **MSTRG.29018** | **71.2274607002838** | **1.06993847554782** | **0.250545552299765** | **4.27043491982526** | **1.95092145379254e-05** | **0.000629772959935614** | **"-"** |  |
| **MSTRG.18653** | **704.061312795007** | **1.06992212076981** | **0.369970483986387** | **2.89191210401848** | **0.00382905053892706** | **0.0309461290404462** | **"-"** |  |
| **MSTRG.20268** | **260.670284569908** | **1.06957342578279** | **0.205225716815529** | **5.21169297093598** | **1.87125059167257e-07** | **1.72217727898669e-05** | **Pole** |  |
| **MSTRG.11111** | **266.99415990221** | **1.06892463246704** | **0.186195436764166** | **5.74087448674124** | **9.41888841837917e-09** | **1.83002342149014e-06** | **"-"** |  |
| MSTRG.25226 | 75.7997070137131 | -1.06670600657207 | 0.392974901441857 | -2.71443800267711 | 0.00663883253801909 | 0.0450751655025758 | Gm17484 |  |
| **MSTRG.13511** | **166.919968735618** | **1.06514493112758** | **0.210871397177534** | **5.05115888349158** | **4.39137599552572e-07** | **3.30733695394616e-05** | **Nrap** |  |
| **MSTRG.28054** | **304.947999484472** | **1.06479787950038** | **0.250381423269502** | **4.25270319816926** | **2.11205422877768e-05** | **0.000668180313989124** | **"-"** |  |
| **MSTRG.18405** | **1880.89694035764** | **1.06339070359914** | **0.160894538384083** | **6.60924052661529** | **3.86296648511553e-11** | **2.47680534470657e-08** | **"-"** |  |
| MSTRG.9169 | 702.989772014797 | -1.06254134604246 | 0.213476433978045 | -4.97732384901903 | 6.44694069939551e-07 | 4.35111945097799e-05 | Arc |  |
| **MSTRG.22912** | **67.1456931754207** | **1.06186503254925** | **0.326242517187074** | **3.25483337274631** | **0.00113458912076423** | **0.0129749237442924** | **Meiosin** |  |
| **ENSMUSG00000031766** | **89.3187735920904** | **1.06005997498455** | **0.349880132044165** | **3.02978042448761** | **0.00244731591776716** | **0.0223205887521343** | **sgk3** |  |
| **MSTRG.5101** | **59.5121807404355** | **1.05923155492852** | **0.359652194563494** | **2.94515526650434** | **0.00322792632995816** | **0.027480348994929** | **Rhbdf2** |  |
| **MSTRG.3082** | **189.820065926278** | **1.05519054942713** | **0.274226211800789** | **3.84788362315149** | **0.000119142622881829** | **0.00251008581723109** | **Acss3** |  |
| **MSTRG.24453** | **200.014278299887** | **1.05486859563174** | **0.212923975085181** | **4.954202997618** | **7.26273552383266e-07** | **4.78420266441511e-05** | **Acsm5** |  |
| **MSTRG.17406** | **243.505845871859** | **1.05466374002806** | **0.304952299627046** | **3.45845478561044** | **0.000543283453728537** | **0.00760557294939476** | **Slc44a5** |  |
| **MSTRG.24330** | **63.9447012546894** | **1.05429703415944** | **0.261094294272012** | **4.03799338893657** | **5.39103720701858e-05** | **0.00138078030195742** | **"-"** |  |
| **MSTRG.7940** | **37.9105095669913** | **1.05373296547593** | **0.350754543665586** | **3.00418906755651** | **0.00266289788578055** | **0.02373187564016** | **"-"** |  |
| **MSTRG.4753** | **36.9699522951127** | **1.05291622473063** | **0.366343914359149** | **2.87411960035561** | **0.00405155505387985** | **0.032145352259237** | **A830036E02Rik** |  |
| **MSTRG.5271** | **189.575729827995** | **1.05253528789713** | **0.261551453135218** | **4.02419973309415** | **5.71693832831242e-05** | **0.00144501062739934** | **"-"** |  |
| MSTRG.1635 | 2376.17135352411 | -1.05237911518407 | 0.18411429631521 | -5.71590113449074 | 1.09124172599952e-08 | 1.98019194335855e-06 | Adamts4 |  |
| **MSTRG.9375** | **44.8212284243488** | **1.04997812616118** | **0.299583359674331** | **3.50479454967919** | **0.000456959770961836** | **0.00667343272164596** | **1110025M09Rik** |  |
| **MSTRG.7689** | **6143.09473515889** | **1.04973223287007** | **0.261070668960671** | **4.02087387698158** | **5.79826443280255e-05** | **0.0014579386292015** | **Fam107a** |  |
| **MSTRG.19145** | **339.622509195785** | **1.04823300531977** | **0.242316358749168** | **4.32588625353535** | **1.51919806488117e-05** | **0.00052181740674981** | **Abcb1b** |  |
| MSTRG.6887 | 32.0984958866283 | -1.04600362155058 | 0.370957075533374 | -2.81974301217088 | 0.00480621235661902 | 0.0362966213897004 | A830005F24Rik |  |
| MSTRG.9347 | 74.2492772968635 | -1.04338560516064 | 0.310741415895872 | -3.35772945538186 | 0.000785854887136943 | 0.00989261698565386 | Gm16576 |  |
| **MSTRG.17994** | **77.6459101081395** | **1.04308749129634** | **0.312948489588873** | **3.33309642320585** | **0.000858851718032005** | **0.0105720899588775** | **"-"** |  |
| **MSTRG.18656** | **130.917191020687** | **1.04278886988487** | **0.252297649096724** | **4.13316918971804** | **3.57795141393842e-05** | **0.00101657157233538** | **Grrp1** |  |
| **MSTRG.28858** | **97.9012892645955** | **1.0427711219384** | **0.303435862320336** | **3.43654541676275** | **0.000589183563039671** | **0.00808341357708137** | **"-"** |  |
| MSTRG.4411 | 2004.23636720434 | -1.04081080058701 | 0.160738691686413 | -6.47517277680437 | 9.47035114038856e-11 | 5.13308875461362e-08 | Myo1d |  |
| **MSTRG.6459** | **1204.88808000649** | **1.03936417498013** | **0.325755579081686** | **3.19062586099102** | **0.00141964991035237** | **0.0151621132846351** | **Net1** |  |
| **MSTRG.6654** | **909.2275241784** | **1.03739044907002** | **0.389323396589851** | **2.66459827011861** | **0.0077080341905412** | **0.0495369320598263** | **H1f2** |  |
| MSTRG.18772 | 3705.06130926355 | -1.03393043865894 | 0.346459798626516 | -2.98427246900735 | 0.00284253523774242 | 0.0250234166123458 | Padi2 |  |
| **MSTRG.10509** | **267.915962419117** | **1.03255541515367** | **0.227976648506396** | **4.52921569782926** | **5.92030368148087e-06** | **0.000251704543793162** | **Btg3** |  |
| MSTRG.27744 | 423.630981907532 | -1.0321583039726 | 0.179130749923815 | -5.76203864725389 | 8.3103962153005e-09 | 1.6651090750136e-06 | Bfsp2 |  |
| **MSTRG.22936** | **89.6069595258818** | **1.03077616693648** | **0.290258912196708** | **3.55123003505894** | **0.000383435129972388** | **0.00588617296489934** | **Klc3** |  |
| MSTRG.1888 | 50.9113568584981 | -1.02670917901198 | 0.314521787221658 | -3.26434994561571 | 0.00109715561628638 | 0.0126445705687648 | Gm37254 |  |
| **MSTRG.24576** | **89.2642485632852** | **1.02516467096148** | **0.345262628650033** | **2.96923149478947** | **0.00298545593839719** | **0.0259373283536902** | **Nupr1** |  |
| MSTRG.10052 | 132.956687929114 | -1.02415432435096 | 0.28724445897958 | -3.56544501498553 | 0.000363239313644891 | 0.00564826855130111 | "-" |  |
| MSTRG.3889 | 1195.03683111683 | -1.02362483323707 | 0.337408729983328 | -3.0337828937848 | 0.00241508162512245 | 0.0221209976472526 | Glra1 |  |
| MSTRG.28173 | 498.698750222681 | -1.02316072401748 | 0.169623883282616 | -6.03193786285835 | 1.62005075263441e-09 | 5.02607681079403e-07 | mt-Tm |  |
| MSTRG.22062 | 338.268741228496 | -1.02260063897872 | 0.316369846463688 | -3.23229489285759 | 0.00122800257193711 | 0.0136659162859229 | Wnt7a |  |
| **MSTRG.21172** | **741.21470979701** | **1.02244192723072** | **0.224600681017677** | **4.55226548111069** | **5.30713058799136e-06** | **0.000231479947528376** | **Pdk4** |  |
| **MSTRG.3988** | **118.016803594453** | **1.01568157674173** | **0.23234128054823** | **4.37150718264589** | **1.23391790285337e-05** | **0.000442806172787024** | **Shmt1** |  |
| MSTRG.24299 | 91.7040290284431 | -1.01397179118314 | 0.324214716561552 | -3.12746997402456 | 0.00176317828674493 | 0.0177008008066486 | "-" |  |
| MSTRG.26899 | 122.738558804221 | -1.01334856526044 | 0.24889470590221 | -4.07139461479178 | 4.67325039909131e-05 | 0.00123475235475991 | "-" |  |
| MSTRG.17280 | 59.8198121463121 | -1.00943904904045 | 0.368649198475319 | -2.73821034526955 | 0.00617745508942468 | 0.0430832552361342 | Dapp1 |  |
| **MSTRG.13220** | **153.169115840239** | **1.00922501908633** | **0.282404127225553** | **3.57369075658118** | **0.000351984604385048** | **0.00549101692242205** | **Fas** |  |
| MSTRG.985 | 1090.22889000167 | -1.00877344937836 | 0.178272866436605 | -5.65859218815605 | 1.52619729044471e-08 | 2.50909443433367e-06 | Cdh20 |  |
| **MSTRG.16834** | **105.411627362832** | **1.00772227047004** | **0.210590944674984** | **4.78521178593559** | **1.70807128508677e-06** | **9.55079975832677e-05** | **"-"** |  |
| MSTRG.7841 | 242.802382655365 | -1.0071658753916 | 0.215100442236364 | -4.682304996309 | 2.83666994607676e-06 | 0.00014396661322635 | Plac9a |  |
| **MSTRG.26033** | **92.9896570219994** | **1.00577017796134** | **0.347514611849609** | **2.89418097445813** | **0.0038014899043288** | **0.0308215800313193** | **Agrp** |  |
| MSTRG.21342 | 328.644332353239 | -1.00507800638053 | 0.237742698182603 | -4.2275872784474 | 2.36210560482827e-05 | 0.000730467866702118 | Mest |  |

The up-regulated DEGs are in bold.
