# Supplementary material for: Novel insights into the nervous system affected by prolonged hyperglycemia
Source: J Mol Med (Berl). 2023 Jul 18;101(8):1015–28. doi: 10.1007/s00109-023-02347-y (PMC10400689; doi:10.1007/s00109-023-02347-y)
Supplement: Supplementary file 5 — Supplementary Table 2. The table with GO-BP terms(DOCX 25 KB) [file 109_2023_2347_MOESM5_ESM.docx]

| **Supplementary Table** **2.** The table with GO-BP terms | | | | |  |
| --- | --- | --- | --- | --- | --- |
| genes with known DAVID ID | | | | |  |
| Term  (categories) | Description | Genes | Count | False Discovery Rate (FDR) | Benjamini |
| GO:0035556 | intracellular signal transduction | **DUSP1**, PKN3, **NET1, SOCS3, ZFP36**, DEPDC1B, DEPDC7, CHN2, BLNK, **SGK3, MYZAP, SGK1, SH2B2** | 13 | 1.0 | 1.0 |
| GO:0006814 | sodium ion transport | **SLC12A3, SLC9A3**, SLC13A3, **SLC12A1, CHP2, ASIC3,** SLC9B2 | 7 | 1.0 | 1.0 |
| GO:0001558 | regulation of cell growth | IGFBP4, **FAM107A, SGK3, SGK1, FBLN5** | 5 | 1.0 | 1.0 |
| GO:0071294 | cellular response to zinc ion | GLRA1, **MT2, MT1** | 3 | 1.0 | 1.0 |
| GO:0055072 | iron ion homeostasis | **STEAP4, TFR2, LCN2,** TRF | 4 | 1.0 | 1.0 |
| GO:0043065 | positive regulation of apoptotic process | TOP2A, **NET1, DUSP1**, LPAR1, **TXNIP, FAS**, PLA2G4A, PDCD1**, NUPR1, SPDEF** | 10 | 1.0 | 1.0 |
| GO:0042127 | regulation of cell proliferation | **NFKBIA**, FA2H, **TXNIP**, WNT7A, **FAS,** PLA2G4A, **SGK3, SGK1** | 8 | 1.0 | 1.0 |
| GO:0010942 | positive regulation of cell death | **CDKN1A**, LPAR1, **FAS,** CIDEB | 4 | 1.0 | 1.0 |
| GO:0007601 | visual perception | GLRA1, BFSP2, **RRH, CNGA3**, RLBP1, **GPR179** | 6 | 1.0 | 1.0 |
| GO:0006811 | ion transport | **SLC12A3**, SLC13A3, **STEAP4,** KCNK13, **SLC12A1**, TRF, **CLCN1, BSPRY**, SLC9B2, **SLC9A3**, GLRA1**, LCN2, CNGA3, ASIC3** | 14 | 1.0 | 1.0 |
| GO:0097460 | ferrous iron import into cell | **TFR2**, TRF | 2 | 1.0 | 1.0 |
| GO:0055085 | transmembrane transport | **SLC12A3, SLC9A3**, SLC13A3, **SLC17A9**, **SLC43A3,** **SLC12A1**, **CNGA3, ABCB1B, CLCN1,** SLC9B2 | 10 | 1.0 | 1.0 |
| GO:0071673 | positive regulation of smooth muscle cell chemotaxis | LPAR1, AIF1 | 2 | 1.0 | 1.0 |
| GO:0070371 | ERK1 and ERK2 cascade | **EGF, TNFSF11**, TRF | 3 | 1.0 | 1.0 |
| GO:0009612 | response to mechanical stimulus | **ACTA1, ANGPT2, TXNIP, ASIC3** | 4 | 1.0 | 1.0 |
| GO:0007165 | signal transduction | GPR17, **ARHGAP8,** GPR34, **LVRN, IL1R1**, UNC5B, PKN3, **RASL11A**, LPAR1**, RRH**, WNT7A, **ICOSL,** **LPAR3,** GNG11, **HCAR2, SOCS3**, DEPDC1B, DEPDC7, GNGT2, CHN2, **VMN2R1, FAS, SH2B2** | 23 | 1.0 | 1.0 |
| GO:0006885 | regulation of pH | **SLC9A3, CHP2, PDK4** | 3 | 1.0 | 1.0 |
| GO:0006629 | lipid metabolic process | **SULT1A1,** FA2H, **ACSM3, PLA2G4E**, PLA2G4A, **APOD,** **ACSM5, TECRL**, PRKAG3, **PLA1A, PLIN5** | 11 | 1.0 | 1.0 |
| GO:0030316 | osteoclast differentiation | **TNFSF11**, TRF, **CD300LF** | 3 | 1.0 | 1.0 |
| GO:0071346 | cellular response to interferon-gamma | **CCL24, CCL19, IL12RB1**, AIF1 | 4 | 1.0 | 1.0 |
| GO:0061030 | epithelial cell differentiation involved in mammary gland alveolus development | FOXB1, **FOXF1** | 2 | 1.0 | 1.0 |
| GO:0010273 | detoxification of copper ion | **MT2, MT1** | 2 | 1.0 | 1.0 |
| GO:2001206 | positive regulation of osteoclast development | **TNFSF11**, SLC9B2 | 2 | 1.0 | 1.0 |
| GO:0008152 | metabolic process | **GYS2**, HK3, **ACSS3**, DCT, **ACSM3**, **PLA2G4E, PAH**, ENPP1, PLA2G4A, **ACSM5, CHIL3** | 11 | 1.0 | 1.0 |
| GO:0042327 | positive regulation of phosphorylation | **EGF, TNFSF11,** TRF | 3 | 1.0 | 1.0 |
| GO:0051482 | positive regulation of cytosolic calcium ion concentration involved in phospholipase C-activating G-protein coupled signaling pathway | GPR17, LPAR1, **LPAR3** | 3 | 1.0 | 1.0 |
| GO:0042771 | intrinsic apoptotic signaling pathway in response to DNA damage by p53 class mediator | **CDKN1A, DDIT4, NUPR1** | 3 | 1.0 | 1.0 |
| GO:0006812 | cation transport | **SLC9A3, CNGA3, ASIC3,** SLC9B2 | 4 | 1.0 | 1.0 |
| GO:0006633 | fatty acid biosynthetic process | FA2H**, ACSM3, ACSM5**, PRKAG3 | 4 | 1.0 | 1.0 |
| GO:0003401 | axis elongation | NAT8F3, NAT8F6 | 2 | 1.0 | 1.0 |
| GO:0090131 | mesenchyme migration | **ACTA1, FOXF1** | 2 | 1.0 | 1.0 |
| GO:0051384 | response to glucocorticoid | **SULT1A1, DUSP1, FAS,** AIF1 | 4 | 1.0 | 1.0 |
| GO:0007257 | activation of JUN kinase activity | **TNFSF11**, TRF, **CCL19** | 3 | 1.0 | 1.0 |
| GO:0016573 | histone acetylation | NAT8F3, NAT8F6, NAT8F7 | 3 | 1.0 | 1.0 |
| GO:0001525 | angiogenesis | **ANGPT2**, UNC5B, **EGF,** WNT7A, **ANGPTL4, HOXB13, APOLD1** | 7 | 1.0 | 1.0 |
| GO:0009749 | response to glucose | **GYS2, ANGPT2**, UCN3, **TXNIP** | 4 | 1.0 | 1.0 |
| GO:0006821 | chloride transport | **SLC12A3**, GLRA1, SLC12A1, CLCN1 | 4 | 1.0 | 1.0 |
| GO:0032411 | positive regulation of transporter activity | **SGK3, SGK1** | 2 | 1.0 | 1.0 |
| GO:0046627 | negative regulation of insulin receptor signaling pathway | **TRIM72, SOCS3**, ENPP1 | 3 | 1.0 | 1.0 |
| GO:0043066 | negative regulation of apoptotic process | BTC, **SOCS3, CDKN1A, DUSP1, HPN**, WNT7A, **FAS, DPEP1, ANGPTL4**, PDCD1, **SGK1,** AIF1 | 12 | 1.0 | 1.0 |
| GO:0042594 | response to starvation | **ZFP36**, UCN3, **PDK4** | 3 | 1.0 | 1.0 |
| GO:0034605 | cellular response to heat | **CDKN1A, HSF3**, MKI67 | 3 | 1.0 | 1.0 |
| GO:0071281 | cellular response to iron ion | **TFR2,** TRF | 2 | 1.0 | 1.0 |
| GO:1900016 | negative regulation of cytokine production involved in inflammatory response | **IL1R2, APOD** | 2 | 1.0 | 1.0 |
| GO:0097066 | response to thyroid hormone | **HPN, CFB** | 2 | 1.0 | 1.0 |
| GO:0002548 | monocyte chemotaxis | **CCL24, TNFSF11, CCL19** | 3 | 1.0 | 1.0 |
| GO:0009636 | response to toxic substance | **CDKN1A**, GJC2, **FAS, NUPR1** | 4 | 1.0 | 1.0 |
| GO:0097192 | extrinsic apoptotic signaling pathway in absence of ligand | UNC5B**, LCN2, FAS** | 3 | 1.0 | 1.0 |
| GO:0007162 | negative regulation of cell adhesion | NAT8F3, NAT8F6, MYO1F | 3 | 1.0 | 1.0 |
| GO:0060012 | synaptic transmission, glycinergic | GLRA1, **SLC6A20A** | 2 | 1.0 | 1.0 |
| GO:0032287 | peripheral nervous system myelin maintenance | FA2H, **PRX** | 2 | 1.0 | 1.0 |
| GO:0048672 | positive regulation of collateral sprouting | **CRABP2, LPAR3** | 2 | 1.0 | 1.0 |
| GO:0019221 | cytokine-mediated signaling pathway | **SOCS3, IL1R1, TNFSF11, IL20RB, SH2B2** | 5 | 1.0 | 1.0 |
| GO:0006879 | cellular iron ion homeostasis | **TFR2, LCN2**, TRF | 3 | 1.0 | 1.0 |

The up-regulated DEGs are in bold.
