# Supplementary material for: Novel insights into the nervous system affected by prolonged hyperglycemia
Source: J Mol Med (Berl). 2023 Jul 18;101(8):1015–28. doi: 10.1007/s00109-023-02347-y (PMC10400689; doi:10.1007/s00109-023-02347-y)
Supplement: Supplementary file 6 — Supplementary Table 3. The table with GO-MF terms (DOCX 17 KB) [file 109_2023_2347_MOESM6_ESM.docx]

| **Supplementary Table** **3.** The table with GO-MF terms | | | | |  |
| --- | --- | --- | --- | --- | --- |
| genes with known DAVID ID | | | | |  |
| Term  (categories) | Description | Genes | Count | False Discovery Rate (FDR) | Benjamini |
| GO:0005215 | transporter activity | **SLC12A3**, SLC13A3, **SLC17A9, CRABP2, SLC12A1, LCN2, APOD, ABCB1B**, RLBP1 | 9 | 1.0 | 1.0 |
| GO:0010485 | H4 histone acetyltransferase activity | NAT8F3, NAT8F6, NAT8F7 | 3 | 1.0 | 1.0 |
| GO:0043565 | sequence-specific DNA binding | TOP2A, FOXB1, **NKX6-3**, HOXD1, **FOXF1, EVX2, TFCP2L1, HOXB13, TBX2, CREB3L4, DMRTA1, MAFF, HSF3, SPDEF** | 14 | 1.0 | 1.0 |
| GO:0015293 | symporter activity | **SLC12A3,** SLC13A3, **SLC12A1, SLC6A20A, SLC1A5** | 5 | 1.0 | 1.0 |
| GO:0016597 | amino acid binding | **HDC, SHMT1, PAH** | 3 | 1.0 | 1.0 |
| GO:0070915 | lysophosphatidic acid receptor activity | LPAR1, **LPAR3** | 2 | 1.0 | 1.0 |
| GO:0015645 | fatty acid ligase activity | **ACSM3, ACSM5** | 2 | 1.0 | 1.0 |
| GO:0019966 | interleukin-1 binding | **IL1R1, IL1R2** | 2 | 1.0 | 1.0 |
| GO:0003996 | acyl-CoA ligase activity | **ACSM3, ACSM5** | 2 | 1.0 | 1.0 |
| GO:0003824 | catalytic activity | **GYS2,** HK3, ACSS3, **ACSM3, HDC, SHMT1**, **PAH, ENPP1, ACSM5, HAO1**, MEST | 11 | 1.0 | 1.0 |
| GO:0015377 | cation:chloride symporter activity | **SLC12A3, SLC12A1** | 2 | 1.0 | 1.0 |
| GO:0047760 | butyrate-CoA ligase activity | **ACSM3, ACSM5** | 2 | 1.0 | 1.0 |
| GO:0004321 | fatty-acyl-CoA synthase activity | **ACSM3, ACSM5** | 2 | 1.0 | 1.0 |
| GO:0004908 | interleukin-1 receptor activity | **IL1R1, IL1R2** | 2 | 1.0 | 1.0 |
| GO:0017081 | chloride channel regulator activity | **SGK3, SGK1** | 2 | 1.0 | 1.0 |
| GO:0043394 | proteoglycan binding | **COL5A3,** CTSK | 2 | 1.0 | 1.0 |
| GO:0019899 | enzyme binding | **NFKBIA,** TOP2A, **SLC12A3,** **HK3, ZFP36, CABYR, TRIB3, MCM10,** CYP2E1 | 9 | 1.0 | 1.0 |

The up-regulated DEGs are in bold.
