# Supplementary material for: Novel insights into the nervous system affected by prolonged hyperglycemia
Source: J Mol Med (Berl). 2023 Jul 18;101(8):1015–28. doi: 10.1007/s00109-023-02347-y (PMC10400689; doi:10.1007/s00109-023-02347-y)
Supplement: Supplementary file 7 — Supplementary Table 4. The table with GO-CC terms (DOCX 18 KB) [file 109_2023_2347_MOESM7_ESM.docx]

| **Supplementary Table** **4.** The table with GO-CC terms | | | | |  |
| --- | --- | --- | --- | --- | --- |
| genes with known DAVID ID | | | | |  |
| Term  (categories) | Description | Genes | Count | False Discovery Rate (FDR) | Benjamini |
| GO:0005576 | extracellular region | **HPN**, TRF, **FBLN5, APOLD1,** ADAMTS4, **ADAMTS14**, UCN3, **LAO1**, PLAC9A, ENPP1, **TNFSF11, NRTN, APOD**, IL33, **CCL24, ANGPT2,** MYOC, IGFBP4, **EGF, IL1R1, GPX3, FST, IL1R2**, GKN3, WNT7A, PRSS35, RNASE1, **ABHD15**, BTC, **LCN2**, **FAS,** TLL2, **AGRP, CHIL3, ANGPTL4, CFB, PLA1A** | 37 | 0.1258629930066948 | 0.1258629930066948 |
| GO:0005615 | extracellular space | TRF, **FBLN5,** PRKAG3, ADAMTS4, NAT8F3, SERPINB1A, UCN3, CTSK, ENPP1, **TNFSF11, APOD, CCL19, GM7298**, IL33, **CCL24, ANGPT2,** MYOC, IGFBP4, **EGF, IL1R1, GPX3**, GKN3, WNT7A, BTC, **ACTA1, LCN2, FAS,** **DPEP1,** SPINK10, **AGRP**, **ANGPTL4, CFB** | 32 | 0.1258629930066948 | 0.1258629930066948 |
| GO:0016324 | apical plasma membrane | **SLC12A3**, **SLC9A3, MUC1**, TMEM235, MYO1A**, HPN, SLC12A1, FAS, DPEP1**, TRF, **SLC6A20A, ABCB1B** | 12 | 0.1258629930066948 | 0.1258629930066948 |
| GO:0030864 | cortical actin cytoskeleton | **GYS2**, MYO1A, **MYZAP,** MYO1F | 4 | 0.7306247680998161 | 0.7306247680998161 |
| GO:0016020 | membrane | **STEAP4, GALNT15, RASL11A, RRH**, GXYLT2, MKI67, **CREB3L4, CHP2**, ENPP1, **TNFSF11, IL1R1**, UNC5B, **IL1R2**, KCNK13, SLC9B2, **TMEM82**, BTC, **SLC9A3**, ARC, **TFR2**, NINJ2, **DPEP1**, CYP2E1, **FKBP5, 1700093K21RIK**, CHODL, **HPN**, LPAR1, DAPP1, **LPAR3,** **SLC1A5**, **EFNA4, HCAR2, FUT7,** GJC2, CDH20, **TSPAN4, CD300LF, ASIC3**, ATP8B5, GPR17**, LVRN**, **TECRL**, GNG11, **BSPRY, ABHD15**, FA2H, **BST1**, BFSP2, GJB1, TMEM235, SPINK10, **SLC44A5, TRIM72, PSCA**, TRF, **SLC6A20A, TFCP2L1,** GNGT2**, PRX**, BLNK, **CNGA3**, SLC13A3, MYOC, GPR34, TMEM255B, **PLA2G4E,** **RHBDF2, ICOSL, ARL4D**, DCT, **RHOJ,** CDC42EP2, **MYZAP**, RAB7B, **SGK1, SLC43A3, DCST1**, AIF1, **CLCN1**, GLRA1, SERPINB1A, **MUC1, SLC17A9**, CHN2, **KLRG2, VMN2R1, IL12RB1**, MEST, **ABCB1B, SH2B2, MPZL2, SLC12A3, EGF, SLC12A1, PLEKHA4,** TM6SF2, **FAS,** PDCD1 | 99 | 0.7587483629476005 | 0.7587483629476005 |
| GO:0048471 | perinuclear region of cytoplasm | CHODL, **CDKN1A**, PKN3, XRCC3, CIDEB, TRF, PLA2G4A, **MT2, MT1**, EML1, AIF1, **BSPRY, FAS, APOD, SGK1** | 15 | 1.0 | 1.0 |
| GO:1990712 | HFE-transferrin receptor complex | **TFR2**, TRF | 2 | 1.0 | 1.0 |
| GO:0072562 | blood microparticle | **ACTA1**, TRF, **ANGPTL4, CFB, OAZ3** | 5 | 1.0 | 1.0 |
| GO:0031410 | cytoplasmic vesicle | **TRIM72**, IL33, ATP8B5, MYOC, TRF, PLA2G4A, MYO1D, ARC, **TFR2, CREB3L4, SGK3, CHIL3**, RAB7B | 13 | 1.0 | 1.0 |
| GO:0005886 | plasma membrane | H2-T24, **TRIM72**, **STEAP4, PSCA, SLC6A20A, GNGT2**, **PRX,** BLNK, **CHP2**, ENPP1, **TNFSF11, CNGA3, POLE, GPR179,** SLC13A3, GPR34, **IL1R1**, UNC5B, **IL1R2**, **RHBDF2**, BTC, **SLC9A3,** **ARL4D**, ARC, **TFR2**, **RHOJ**, CDC42EP2, **DPEP1, MYZAP, SGK1, HPN**, LPAR1, DAPP1, **ZBTB42, IL20RB, LPAR3, SLC1A5, EFNA4**, AIF1, **CLCN1, HCAR2**, GLRA1, **MUC1**, GJC2, CDH20, **TSPAN4,** **VMN2R1, CD300LF**, LY6G6E, **ASIC3, ABCB1B, SH2B2, MPZL2, SLC12A3**, ATP8B5, GPR17, **ANGPT2, EGF, LVRN, SLC12A1**, GNG11, **NET1, NFKBIA, BST1**, BFSP2, GJB1, **FAS**, PDCD1 | 68 | 1.0 | 1.0 |

The up-regulated DEGs are in bold
