# Supplementary material for: Novel insights into the nervous system affected by prolonged hyperglycemia
Source: J Mol Med (Berl). 2023 Jul 18;101(8):1015–28. doi: 10.1007/s00109-023-02347-y (PMC10400689; doi:10.1007/s00109-023-02347-y)
Supplement: Supplementary file 8 — Supplementary Table 5. Functional enrichment analysis of DEGs in diabetic spinal cord (SC) of mice by KEGG database (DOCX 19 KB) [file 109_2023_2347_MOESM8_ESM.docx]

**Supplementary Table 5.** Functional enrichment analysis of DEGs in diabetic spinal cord (SC) of mice by KEGG database

| Kegg catergory | Kegg subcategory | Kegg pathways | Altered genes | Count | Benijamini | p-value | FDR |
| --- | --- | --- | --- | --- | --- | --- | --- |
| mmu09160: Human Diseases | mmu09161: Cancer: overview | mmu05200: Pathways in cancer | **NFKBIA, CDKN1A**, GNGT2, **EGF**, LPAR1, WNT7A, **FAS, LPAR3,** GNG11 | 9 | 9.8E-1 | 9.9E-2 | 0.9836252195569368 |
|  |  | mmu05215 Prostate cancer | **NFKBIA, CDKN1A, EGF, CREB3L4** | 4 | 9.6E-1 | 8.8E-2 | 0.9590530743649162 |
|  | mmu09172 Infectious disease: viral | mmu05166 Human T-cell leukemia virus 1 infection | H2-T24, **NFKBIA, ZFP36, CDKN1A, IL1R1, IL1R2,** WNT7A, **POLE** | 8 | 8.7E-1 | 4.7E-2 | 0.8722764667435894 |
|  | mmu09174 Infectious disease: parasitic | mmu05146 Amoebiasis | SERPINB1A, **IL1R1, IL1R2, COL5A3,** RAB7B | 5 | 8.7E-1 | 5.1E-2 | 0.8722764667435894 |
|  | mmu09167 Endocrine and metabolic disease | mmu04931 Insulin resistance | **NFKBIA, GYS2, SOCS3, CREB3L4, TRIB3,** PRKAG3 | 6 | 7.0E-1 | 1.0E-2 | 0.6951509509082803 |
| mmu09130 Environmental Information Processing | mmu09131 Membrane transport | mmu04068: FoxO signaling pathway | **CDKN1A, EGF, SGK3, SGK1**, PRKAG3 | 5 | 9.6E-1 | 7.6E-2 | 0.9590530743649162 |
|  | mmu09132 Signal transduction | mmu04010: MAPK signaling pathway | **IL1R1, DUSP1, PLA2G4E, EGF, FAS,** PLA2G4A, **MAP3K6** | 7 | 9.6E-1 | 8.0E-2 | 0.9590530743649162 |
|  |  | mmu04064 NF-kappa B signaling pathway | **NFKBIA, IL1R1**, BLNK, **TNFSF11, CCL19** | 5 | 7.7E-1 | 2.9E-2 | 0.767856675627659 |
|  |  | mmu04066 HIF-1 signaling pathway | HK3, **CDKN1A, ANGPT2, EGF**, TRF | 5 | 7.9E-1 | 3.4E-2 | 0.788115287348024 |
|  |  | mmu04014 Ras signaling pathway | **ANGPT2,** GNGT2, **PLA2G4E, EGF,** PLA2G4A, GNG11, **EFNA4, PLA1A** | 8 | 7.3E-1 | 1.9E-2 | 0.732693272162321 |
|  |  | mmu04151 PI3K-Akt signaling pathway | **CDKN1A, ANGPT2, EGF**, PKN3, LPAR1, **LPAR3, EFNA4,** GNG11, **GYS2**, GNGT2, **CREB3L4, COL5A3, DDIT4, SGK3, SGK1** | 15 | 1.2E-2 | 6.5E-5 | 0.012146059959682301 |
|  | mmu09133 Signaling molecules and interaction | mmu04060 Cytokine-cytokine receptor interaction | **CCL24, IL1R1, IL1R2, FAS, TNFSF11,** **IL20RB, CCL19, IL12RB1** | 8 | 7.7E-1 | 2.5E-2 | 0.767856675627659 |
| mmu09150 Organismal Systems | mmu09152 Endocrine system | mmu04910 Insulin signaling pathway | **GYS2**, HK3, **SOCS3, SH2B2,** PRKAG3 | 5 | 9.6E-1 | 8.6E-1 | 0.9590530743649162 |
|  |  | mmu04920 Adipocytokine signaling pathway | **NFKBIA, SOCS3, AGRP,** PRKAG3 | 4 | 8.7E-1 | 5.5E-2 | 0.8722764667435894 |
|  | mmu09158 Development and regeneration | mmu04380 Osteoclast differentiation | **NFKBIA, SOCS3, IL1R1**, CTSK, BLNK, **TNFSF11** | 6 | 7.3E-1 | 1.7E-1 | 0.73269327216232 |
|  | mmu09154 Digestive system | mmu04978 Mineral absorption | **SLC9A3,** TRF, **MT2, MT1** | 4 | 7.0E-1 | 1.1E-2 | 0.6951509509082803 |
| mmu09100 Metabolism | mmu09103 Lipid metabolism | mmu00590 Arachidonic acid metabolism | **GPX3, PLA2G4E**, PLA2G4A, CYP2E1 | 4 | 9.6E-2 | 9.0E-1 | 0.9590530743649162 |
|  | mmu09105 Amino acid metabolism | mmu00400 Phenylalanine, tyrosine and tryptophan biosynthesis | **LAO1, PAH** | 2 | 9.6E-1 | 9.2E-2 | 0.9590530743649162 |
|  | mmu09101 Carbohydrate metabolism | mmu00500 Starch and sucrose metabolism | **GYS2**, HK3, ENPP1 | 3 | 8.7E-1 | 5.6E-2 | 0.8722764667435894 |

The analysis revealed, that among 19 signaling pathway, the PI3K-Akt signaling pathway is the most significantly enrichment biological axis in diabetic spinal cord. The up-regulated genes are in bold.
