# Supplementary material for: Novel insights into the nervous system affected by prolonged hyperglycemia
Source: J Mol Med (Berl). 2023 Jul 18;101(8):1015–28. doi: 10.1007/s00109-023-02347-y (PMC10400689; doi:10.1007/s00109-023-02347-y)
Supplement: Supplementary file 9 — Supplementary Table 6. The table of interaction network in mouse diabetic spinal cord (SC) by GeneMANIA analysis (DOCX 20 KB) [file 109_2023_2347_MOESM9_ESM.docx]

| **Supplementary Table 6.** The table of interaction network in mouse diabetic spinal cord (SC) by GeneMANIA analysis | | |
| --- | --- | --- |
| GENE 1 | GENE 2 | INTERACTION |
| *HOXB13* | *ACTB* | CO-EXPRESSION |
| *ACTG1* | *HOXB13* | CO-EXPRESSION |
| *TMSB4X* | *DIAPH1* | CO-EXPRESSION |
| *NBEAL2* | *DIAPH1* | CO-EXPRESSION |
| *SUSD6* | *TXNIP* | CO-EXPRESSION |
| *DAPP1* | *CTSE* | CO-EXPRESSION |
| *ANO1* | *RHOJ* | CO-EXPRESSION |
| *WFIKKN1* | *ACTB* | CO-EXPRESSION |
| *HSD17B6* | *HAO1* | CO-EXPRESSION |
| *ATXN7L2* | *ACTB* | CO-EXPRESSION |
| *ATXN7L2* | *HOXB13* | CO-EXPRESSION |
| *MYB* | *DIAPH1* | CO-EXPRESSION |
| *MYB* | *CTSE* | CO-EXPRESSION |
| *EEF1A1* | *ACTG1* | CO-EXPRESSION |
| *VASP* | *DIAPH1* | CO-EXPRESSION |
| *VASP* | *MYB* | CO-EXPRESSION |
| *SDR9C7* | *HAO1* | CO-EXPRESSION |
| *SDR9C7* | *HSD17B6* | CO-EXPRESSION |
| *RAC1* | *VASP* | CO-EXPRESSION |
| *MLXIP* | *TXNIP* | CO-EXPRESSION |
| *TNS1* | *SUSD6* | CO-EXPRESSION |
| *TGFB1I1* | *RHOJ* | CO-EXPRESSION |
| *NET1* | *TXNIP* | CO-EXPRESSION |
| *TMSB4X* | *ACTB* | CO-EXPRESSION |
| *ACTG1* | *ACTB* | CO-EXPRESSION |
| *ACTG1* | *TMSB4X* | CO-EXPRESSION |
| *SUSD6* | *TMSB4X* | CO-EXPRESSION |
| *DAPP1* | *DIAPH1* | CO-EXPRESSION |
| *ANO1* | *TXNIP* | CO-EXPRESSION |
| *ANO1* | *HOXB13* | CO-EXPRESSION |
| *HSD17B6* | *HAO1* | CO-EXPRESSION |
| *EEF1A1* | *ACTB* | CO-EXPRESSION |
| *EEF1A1* | *ACTG1* | CO-EXPRESSION |
| *NRIP1* | *TXNIP* | CO-EXPRESSION |
| *NRIP1* | *HOXB13* | CO-EXPRESSION |
| *VASP* | *ACTB* | CO-EXPRESSION |
| *VASP* | *TMSB4X* | CO-EXPRESSION |
| *VASP* | *SUSD6* | CO-EXPRESSION |
| *RAC1* | *ACTB* | CO-EXPRESSION |
| *RAC1* | *TMSB4X* | CO-EXPRESSION |
| *RAC1* | *ACTG1* | CO-EXPRESSION |
| *MLXIP* | *TXNIP* | CO-EXPRESSION |
| *TNS1* | *TXNIP* | CO-EXPRESSION |
| *TGFB1I1* | *RHOJ* | CO-EXPRESSION |
| *AGER* | *RHOJ* | CO-EXPRESSION |
| *TMSB4X* | *ACTB* | CO-EXPRESSION |
| *ACTG1* | *ACTB* | CO-EXPRESSION |
| *ACTG1* | *TMSB4X* | CO-EXPRESSION |
| *SUSD6* | *ACTB* | CO-EXPRESSION |
| *DAPP1* | *RHOJ* | CO-EXPRESSION |
| *ANO1* | *TXNIP* | CO-EXPRESSION |
| *EEF1A1* | *ACTB* | CO-EXPRESSION |
| *EEF1A1* | *ACTG1* | CO-EXPRESSION |
| *TNS1* | *DIAPH1* | CO-EXPRESSION |
| *RHOJ* | *ACTB* | CO-EXPRESSION |
| *TMSB4X* | *ACTB* | CO-EXPRESSION |
| *SUSD6* | *DIAPH1* | CO-EXPRESSION |
| *SUSD6* | *NET1* | CO-EXPRESSION |
| *RAC1* | *ACTB* | CO-EXPRESSION |
| *TGFB1I1* | *ACTB* | CO-EXPRESSION |
| *AGER* | *HAO1* | CO-EXPRESSION |
| *ACTG1* | *DIAPH1* | CO-EXPRESSION |
| *ANO1* | *TXNIP* | CO-EXPRESSION |
| *ANO1* | *RHOJ* | CO-EXPRESSION |
| *WFIKKN1* | *HAO1* | CO-EXPRESSION |
| *WFIKKN1* | *AGER* | CO-EXPRESSION |
| *VASP* | *ACTB* | CO-EXPRESSION |
| *RHOJ* | *ACTB* | CO-EXPRESSION |
| *SUSD6* | *DIAPH1* | CO-EXPRESSION |
| *SUSD6* | *ACTG1* | CO-EXPRESSION |
| *EEF1A1* | *CTSE* | CO-EXPRESSION |
| *NRIP1* | *ACTB* | CO-EXPRESSION |
| *VASP* | *ACTB* | CO-EXPRESSION |
| *RHOJ* | *TXNIP* | CO-EXPRESSION |
| *TMSB4X* | *ACTB* | CO-EXPRESSION |
| *ACTG1* | *ACTB* | CO-EXPRESSION |
| *MYB* | *DIAPH1* | CO-EXPRESSION |
| *EEF1A1* | *ACTB* | CO-EXPRESSION |
| *EEF1A1* | *ACTG1* | CO-EXPRESSION |
| *SDR9C7* | *HAO1* | CO-EXPRESSION |
| *RAC1* | *TMSB4X* | CO-EXPRESSION |
| *RAC1* | *VASP* | CO-EXPRESSION |
| *TNS1* | *TXNIP* | CO-EXPRESSION |
| *TGFB1I1* | *RHOJ* | CO-EXPRESSION |
| *HOXB13* | *DIAPH1* | CO-EXPRESSION |
| *TGFB1I1* | *ACTG1* | CO-EXPRESSION |
| *ACTB* | *DIAPH1* | PREDICTED |
| *NET1* | *DIAPH1* | PREDICTED |
| *NET1* | *ACTB* | PREDICTED |
| *RHOT2* | *CTSE* | PREDICTED |
| *ACTG1* | *DIAPH1* | PREDICTED |
| *ACTG1* | *ACTB* | PREDICTED |
| *ACTG1* | *NET1* | PREDICTED |
| *NBEAL2* | *DIAPH1* | PREDICTED |
| *DAPP1* | *TXNIP* | PREDICTED |
| *HSD17B6* | *CTSE* | PREDICTED |
| *MYB* | *TXNIP* | PREDICTED |
| *MYB* | *DAPP1* | PREDICTED |
| *EEF1A1* | *DIAPH1* | PREDICTED |
| *EEF1A1* | *ACTB* | PREDICTED |
| *EEF1A1* | *NET1* | PREDICTED |
| *EEF1A1* | *ACTG1* | PREDICTED |
| *NRIP1* | *HOXB13* | PREDICTED |
| *NRIP1* | *MYB* | PREDICTED |
| *VASP* | *DIAPH1* | PREDICTED |
| *VASP* | *TXNIP* | PREDICTED |
| *VASP* | *DAPP1* | PREDICTED |
| *VASP* | *MYB* | PREDICTED |
| *PTPRO* | *ACTB* | PREDICTED |
| *SDR9C7* | *CTSE* | PREDICTED |
| *RAC1* | *ACTB* | PREDICTED |
| *RAC1* | *TXNIP* | PREDICTED |
| *RAC1* | *DAPP1* | PREDICTED |
| *RAC1* | *MYB* | PREDICTED |
| *TNS1* | *HOXB13* | PREDICTED |
| *TNS1* | *NRIP1* | PREDICTED |
| *TGFB1I1* | *DIAPH1* | PREDICTED |
| *TGFB1I1* | *ACTB* | PREDICTED |
| *TGFB1I1* | *NET1* | PREDICTED |
| *TGFB1I1* | *ACTG1* | PREDICTED |

*In silico* analysis revealed, that the most common interaction in diabetic spinal cord is co-expression, *i.e.* simultaneous expression of two or more genes.
