# Supplementary material for: Novel insights into the nervous system affected by prolonged hyperglycemia
Source: J Mol Med (Berl). 2023 Jul 18;101(8):1015–28. doi: 10.1007/s00109-023-02347-y (PMC10400689; doi:10.1007/s00109-023-02347-y)
Supplement: Supplementary file 10 — Supplementary file 1: Western blot raw data (DOCX 3728 KB) [file 109_2023_2347_MOESM10_ESM.docx]

**Western blot raw data**

Uncropped blots for western blot panels presented in main figures. Western blots and densitometry analysis of proteins in sciatic nerves of control (CTR) and experimentally treated mice (STZ). An equal amount of protein (40 µg) was fractionated on 15-well 4–15% Mini-PROTEAN® TGX™ Precast Protein Gels (Bio-Rad, CA, USA) and transferred to nitrocellulose membranes. The bands were visualized with ChemiDoc Imag-ing Systems (Bio-Rad); automatic exposures. Images were quantified densitometrically with ImageJ Software 1.50i (Wayne Rasband, MD, USA) and compared to experimental condition after normalization to the total amount of protein in a sample. In some cases, the membrane has been cut to allow for more analyzes. Uncropped blots for western blot panels presented in main figures are outlined. Figure panels are indicated above the corresponding western blot set. Wight dashed lines indicate where gels were cut in the cases where multiple antibodies were probed simultaneously. Loading controls and the respective experimental samples were run on the same blots.
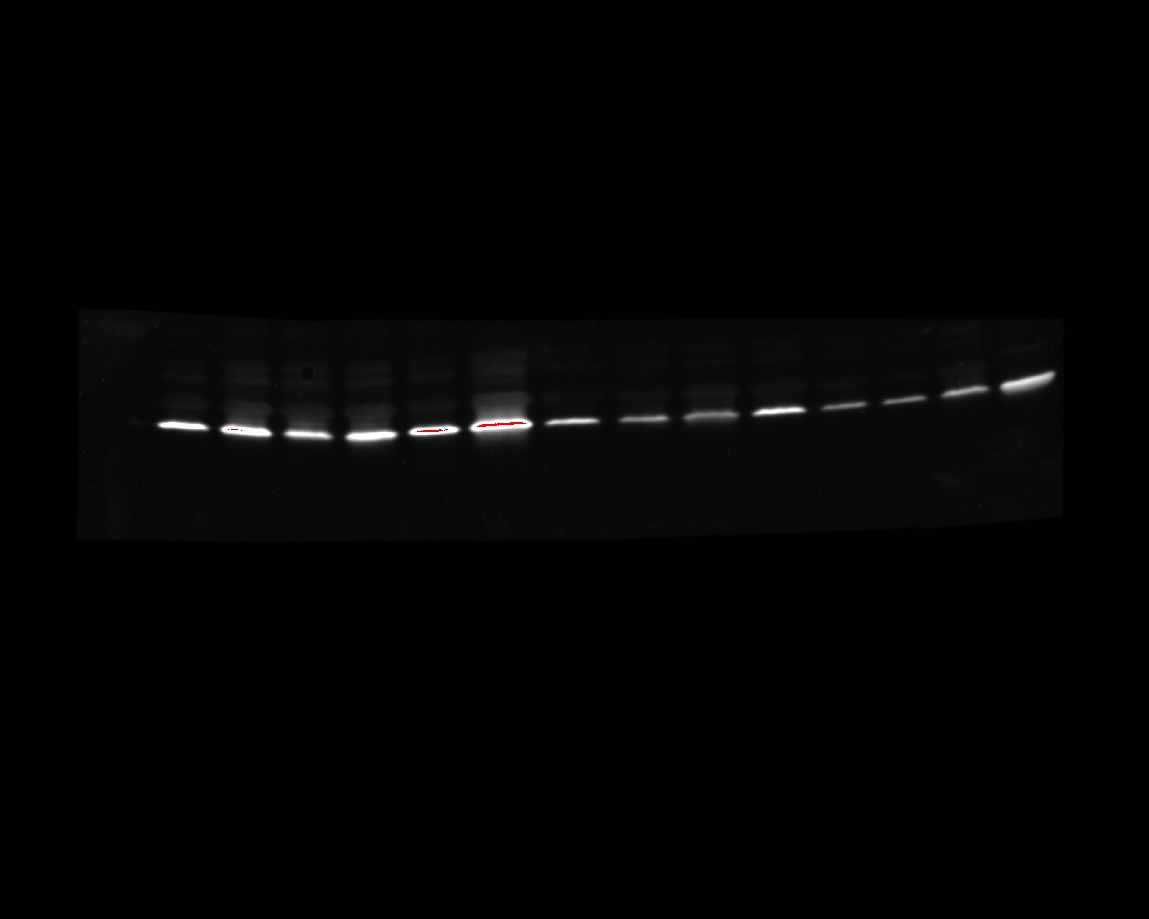
 Red squares where the band is cut to represent it in the main figure (Fig. 3I). DIAPH1 – Diaphanous Related Formin 1, S100B – S100 calcium-binding protein B, HMGB1 – High Mobility Group Box 1, CML – N(epsilon)-(carboxymethyl)lysine, S100A6 – S100 Calcium Binding Protein A6, SOD1 – superoxide dismutase type 1, STZ – streptozotocin (diabetes type 1), CTR – control.


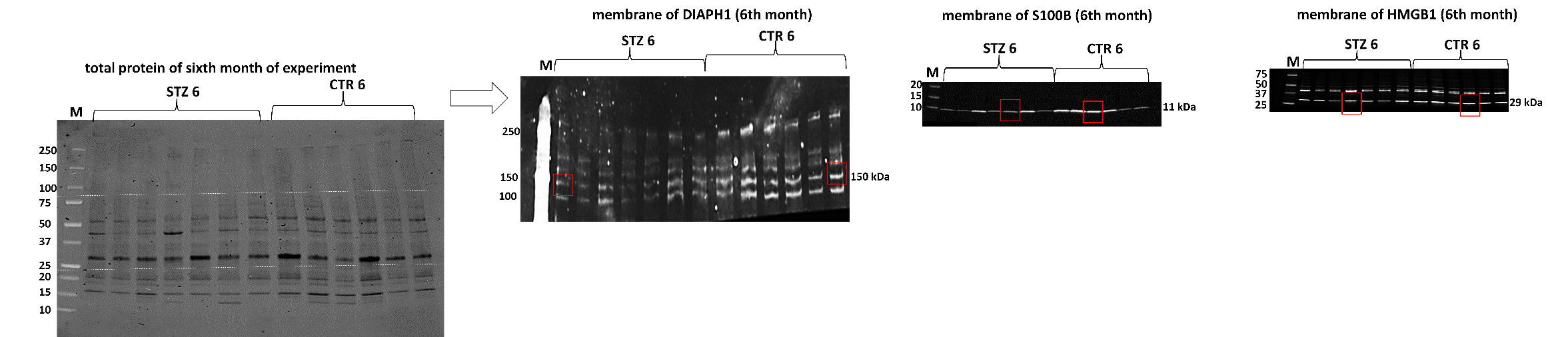


1 2 3 4 5 6 7 8 9 10 11 12 13


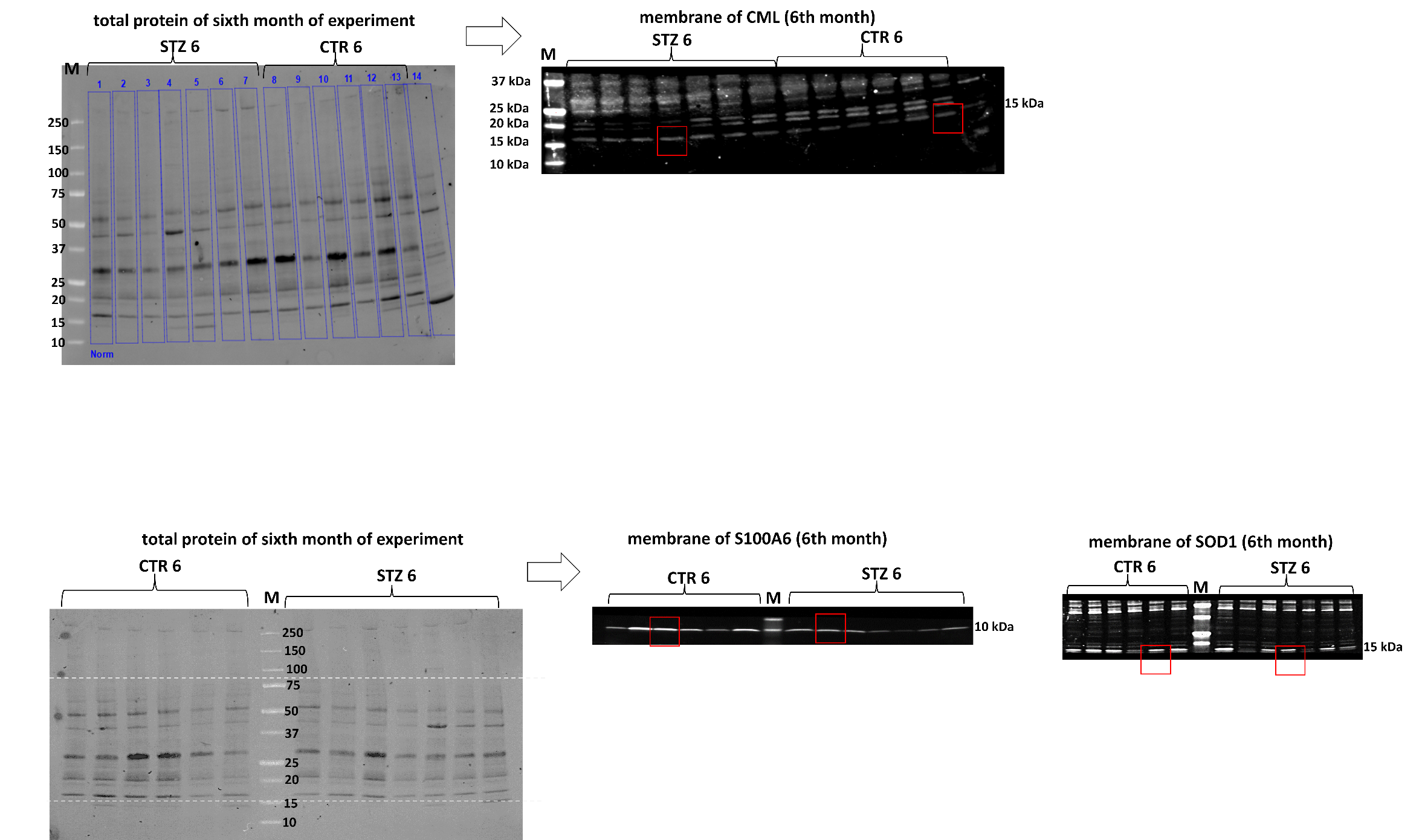


1 2 3 4 5 6 7

1 2 3 4 5 6


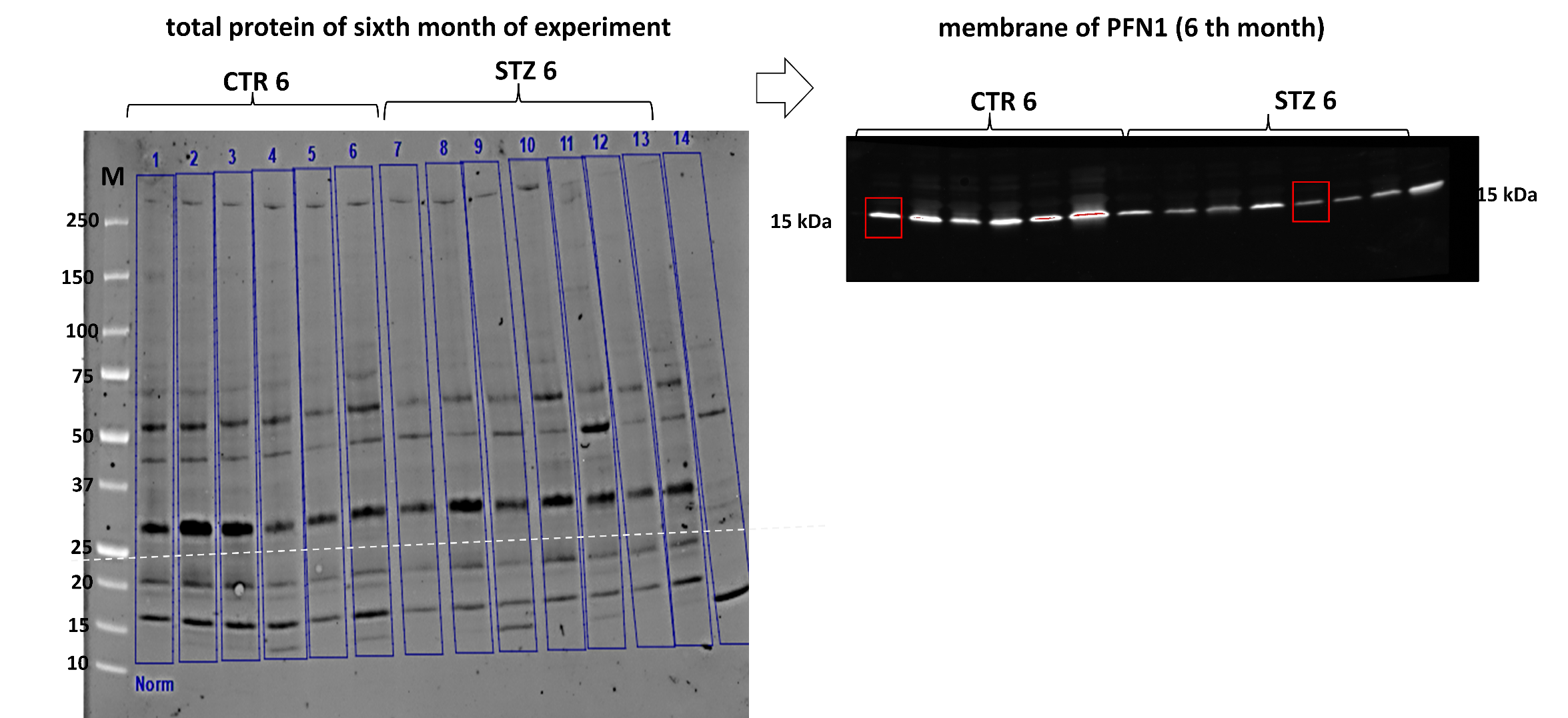


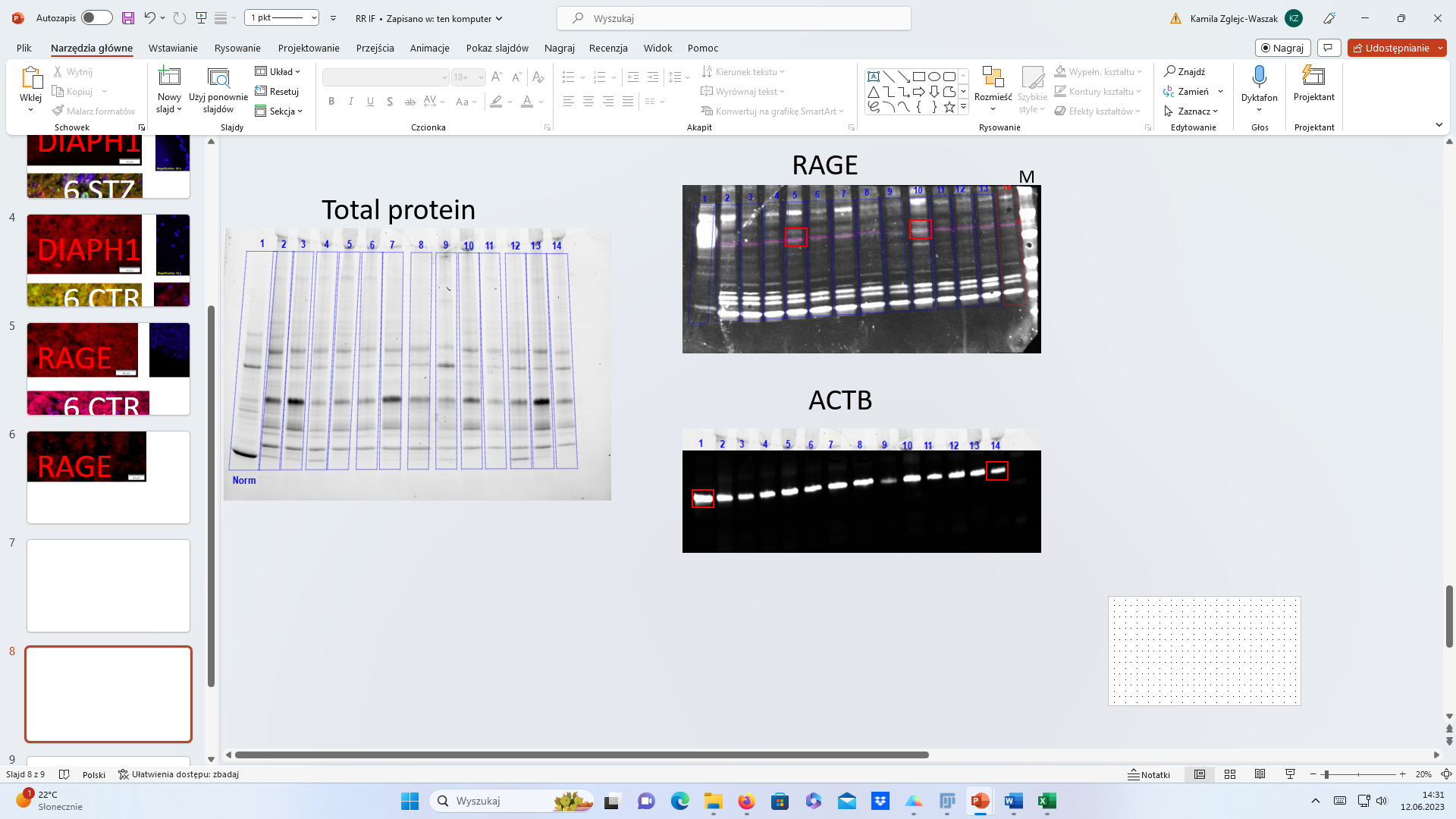
 Abbreviations: 1 – positive control for RAGE protein, *i.e.* lungs, 2-7 – 6 CTR, 8-14 – 6 STZ

**Table of western blot raw data**

Images were quantified densitometrically with ImageJ Software 1.50i (Wayne Rasband, MD, USA) and compared to experimental condition after normalization to the total amount of protein in a sample. DIAPH1 – Diaphanous Related Formin 1, S100B – S100 calcium-binding protein B, HMGB1 – High Mobility Group Box 1, CML – N(epsilon)-(carboxymethyl)lysine, S100A6 – S100 Calcium Binding Protein A6, SOD1 – superoxide dismutase type 1, STZ – streptozotocin (diabetes type 1), CTR – control.

| Test protein | Number of sample  STZ | STZ – value  (test protein/total protein) | Number of sample  CTR | CTR – value  (test protein/total protein) |
| --- | --- | --- | --- | --- |
| Diaph1 | 1 | \| 95,13 \| \| --- \| | 8 | \| 446,152 \| \| --- \| |
|  | 2 | \| 106,0059 \| \| --- \| | 9 | 693,3131 |
|  | 3 | \| 90,7409 \| \| --- \| | 10 | 629,3328 |
|  | 4 | \| 28,464 \| \| --- \| | 11 | 487,945 |
|  | 5 | \| 60,50672 \| \| --- \| | 12 | 583,9879 |
|  | 6 | \| 416,3836 \| \| --- \| | 13 | \| 476,203 \| \| --- \| |
|  | 7 | \| 542,4111 \| \| --- \| | - | - |
| S100B | 1 | \| 265,697 \| \| --- \| | 8 | \| 793,7217 \| \| --- \| |
|  | 2 | 292,6702 | 9 | 1573,577 |
|  | 3 | 749,6712 | 10 | 1420,229 |
|  | 4 | 296,754 | 11 | 813,5958 |
|  | 5 | 469,7516 | 12 | 449,0248 |
|  | 6 | 1033,321 | 13 | \| 356,7531 \| \| --- \| |
|  | 7 | 327,2462 | - | - |
| HMGB1 | 1 | \| 4988,547 \| \| --- \| | 8 | \| 4933,876 \| \| --- \| |
|  | 2 | 8091,417 | 9 | 12342,86 |
|  | 3 | 2956,432 | 10 | 11370,97 |
|  | 4 | 7294,098 | 11 | 5195,496 |
|  | 5 | 7946,963 | 12 | 9068,496 |
|  | 6 | 5470,955 | 13 | \| 10267,51 \| \| --- \| |
|  | 7 | \| 9521,353 \| \| --- \| | - | - |
| CML | 1 | \| 9754,077 \| \| --- \| | 8 | \| 9065,868 \| \| --- \| |
|  | 2 | 19775,06 | 9 | 20724,74 |
|  | 3 | 26323,17 | 10 | 9617,513 |
|  | 4 | 18237,27 | 11 | 13264,44 |
|  | 5 | 21395,38 | 12 | 15593,42 |
|  | 6 | 19610,54 | 13 | 22096,67 |
|  | 7 | \| 14409,19 \| \| --- \| | - | - |
| S100A6 | 1 | \| 739,0459 \| \| --- \| | 1 | \| 412,6043 \| \| --- \| |
|  | 2 | 807,3371 | 2 | 1491,952 |
|  | 3 | 288,4507 | 3 | 628,291 |
|  | 4 | 318,5214 | 4 | 380,1037 |
|  | 5 | 73,73338 | 5 | 8,850311 |
|  | 6 | 346,9666 | 6 | 1999,851 |
|  | 7 | \| 493,5449 \| \| --- \| | - | - |
| SOD1 | 1 | \| 172,8304 \| \| --- \| | 1 | \| 156,3345 \| \| --- \| |
|  | 2 | 60,26163 | 2 | 257,9805 |
|  | 3 | 124,8396 | 3 | 165,2853 |
|  | 4 | 70,46887 | 4 | 56,50366 |
|  | 5 | 18,32289 | 5 | 82,86584 |
|  | 6 | 57,67217 | 6 | 164,8396 |
|  | 7 | 50,43846 | - | - |
| PFN1 | 7 | \| 8278,965 \| \| --- \| | 1 | \| 8820,084 \| \| --- \| |
|  | 8 | 3817,64 | 2 | 10784,55 |
|  | 9 | 5500,452 | 3 | 7439,724 |
|  | 10 | 7288,991 | 4 | 14155,64 |
|  | 11 | 2853,856 | 5 | 16434,23 |
|  | 12 | 3628,142 | 6 | 20878,48 |
|  | 13 | \| 4750,413 \| \| --- \| | - | - |
| RAGE | 8 | 488,2423 | 2 | \| 540,5984 \| \| --- \| |
|  | 9 | 467,4032 | 3 | 174,0488 |
|  | 10 | 1461,556 | 4 | 531,9798 |
|  | 11 | 632,8842 | 5 | 844,7766 |
|  | 12 | 373,3332 | 6 | 616,1709 |
|  | 13 | 381,7066 | 7 | \| 625,7401 \| \| --- \| |
|  | 14 | 637,9687 | - | - |
| ACTB | 8 | 1592,526 | 2 | 828,6879 |
|  | 9 | 465,5038 | 3 | 652,9786 |
|  | 10 | 1777,784 | 4 | 1418,078 |
|  | 11 | 1458,627 | 5 | 2010,547 |
|  | 12 | 1105,984 | 6 | 1994,298 |
|  | 13 | 853,8778 | 7 | 1432,978 |
|  | 14 | 964,2549 | - | - |
