# Supplementary material for: Novel insights into the nervous system affected by prolonged hyperglycemia
Source: J Mol Med (Berl). 2023 Jul 18;101(8):1015–28. doi: 10.1007/s00109-023-02347-y (PMC10400689; doi:10.1007/s00109-023-02347-y)
Supplement: Supplementary file 11 — Supplementary file 2: Detailed methodology (DOCX 24 KB) [file 109_2023_2347_MOESM11_ESM.docx]

*Detailed methodology*

*Animals*

All experiments were approved by the Local Ethics Committee of Experiments on Animals in Olsztyn (Poland; decision no. 57/2019) and were carried out in accordance with the 3R principles (Replacement, Reduction and Refinement). Eight weeks old C57BL/6 males were randomly divided into control and experimental groups per defined time points (Fig. 1A). Animals were injected intraperitoneally with streptozotocin (STZ, 50mg/kg; Sigma-Aldrich, USA) or vehicle (PBS, pH 7.4; Eurx^®^, Poland) for five consecutive days and sacrificed eight, 16 and 24 weeks post the last STZ injection (two, four and six months of rendered diabetes, respectively). All animals received a standard, balanced diet (Labofeed B, Morawski, Poland) and were housed in a barrier animal facility with 12-h light cycle. To determine the diabetes status of the animals, blood glucose was measured seven days after completion of STZ injection cycle and then every third week for the duration of the experiment, using a standard glucometer (Accu-Chek®, Roche, Switzerland). Animals with a blood glucose level ≥ 13 mmol/L (260 mg/dL) were considered diabetic and were used for further experiments. Mice were sacrificed with a mixture of ketamine (300mg/kg, Narketan®, Vétoquinol Biowet, Poland) and xylazine (30mg/kg, VetaXyl®, Vet-Agro, Poland). Both SNs and lumbar SC were collected and snap‐frozen in liquid nitrogen and stored at −80°C for further analysis. In addition, samples of SN were also collected, postfixed in suitable fixative for morphometric and ultrastructural analysis.

*Nerve conduction velocity (NCV)*

Measurement of SN electrophysiological activities was performed with modifications according to the Diabetes Complications Consortium Nerve Conduction protocol (www.diacomp.org). Prior to NCV studies, all animals (n = 12) were intraperitoneally anesthetized with the mixture of ketamine (100 mg/kg) and xylazine (10 mg/kg) diluted in saline.

*Motor nerve conduction velocity (MNCV)*

For MNCV studies, SN was stimulated twice, *i.e.* proximal and distal stimulation, with recording from the same site.

*Sensory nerve conduction velocity (SNCV)*

For SNCV, the sural nerve was stimulated orthodromically using needle electrodes placed in the fourth toe of the foot, with recording *via* needle electrodes in the gastrocnemius muscle.

*Ultrastructural studies – scanning electron microscopy*

Nerve samples (n = 5 per group; at 6 months of experiment) were immersion-fixed in a mixture of 1% paraformaldehyde and 2.5% glutaraldehyde in 0.2 M cacodylate buffer (pH 7.4) for 2 h at 4 °C.

*Scanning electron microscopy (SEM)*

Samples for SEM imaging were post-fixed in a solution containing 2% aqueous osmium tetroxide and 1.5% potassium ferrocyanide in 0.15 M cacodylate buffer with 2 mM calcium chloride for 1 h, washed in water and placed in freshly prepared, filtered 1% thiocarbohydrazide solution for 20 min. Next, samples were rinsed in water, incubated in 2% osmium tetroxide for 30 min., rinsed again in water and placed in 1% aqueous uranyl acetate for overnight incubation at 4°. The following day, samples were *en bloc* stained according to Walton’s lead aspartate method for 30 min., washed in water, dehydrated and embedded in Epon 812 for sectioning in a longitudinal plane. The ultrathin sections were cut using PT3D PowerTome ultramicrotome with ASH2 (Boeckeler Instruments, Inc., Tucson, AR, USA) and placed on silicon wafer. Whole sections were imaged using a backscatter detector in SEM Gemini 450 at 1.5 kV controlled by Atlas 5 software (Carl Zeiss, Oberkochen Germany). Digital ultrastructural images of longitudinal sections obtained in SEM were used for the semiquantitative analysis of myelin structure alternations, which was performed in the ATLAS Browser-Based Viewer 3.0.

*The morphometric analysis of semithin sections*

Semithin sections (n = 5 per group) were cut using Leica Ultracut III ultramicrotome. Semithin sections were stained with 1% toluidine blue and digitalized at 40x objective using PANNORAMIC 250 Flash III scanner (3DHistech, Budapest, Hungary). Digital scans of semithin sections were used for measurements of cross section perimeter of nerve fiber and the thickness of the myelin sheet, for counting of the myelinated fiber per area unit. The right upper part of the semithin section with the area of 25 000 µm^2^ was chosen for analysis and all myelinated fibers located inside this area were measured. No less than 200 nerve fibers were measured per animal. Measurements were made using CaseViewer 2.1 (3DHistech Ltd, Budapest, Hungary).

The count of total number of myelinated axons in mouse SN was performed as described in Juranek et al. [5]. Full-size cross-sections of distal SN fascicule from each of three control and experimental groups were photographed and analyzed using the ImageJ software (National Institutes of Health and Laboratory for Optical and Computational Instrumentation).

*The expression of Diaph1, RAGE and beta-actin (ACTB) in SN*

*RNA isolation*

Total RNA was isolated from a whole, unilateral SN (n = 4 per each control and STZ-treated group) with the use of TRI Reagent® (Sigma Aldrich, USA), according to the manufacturer’s protocol. The quantity of isolated RNA was measured on Infinite 200 mol/L PRO spectrophotometer (TECAN, Switzerland). RNA quality and integrity were confirmed by visualized on 1.5% agarose gel to rule out RNA degradation or DNA contamination.

*Reverse transcription*

Reverse transcription was conducted using the QuantiNova Reverse Transcription Kit (Qiagen, Valencia, CA, USA), according to the manufacturer’s protocol. Briefly, RNA samples, *i.e.* 1 μg were incubated in QuantiNova gDNA Removal Mix at 45°C for 2 min to effectively reduce contaminating gDNA. After gDNA removal, RNA samples were ready for reverse transcription using a reverse-transcription master mix prepared from QuantiNova Reverse Transcription Enzyme and QuantiNova Reverse Transcription Mix. After 3 min primer annealing step at 25°C, the reactions took place at 45°C for 20 min and were then inactivated at 85°C. Next, the samples were diluted and used for qPCR analysis.

*The relative expression of Diaph1, gene encoding RAGE (AGER) and ACTB mRNAs*

The expression of *Diaph1*, *AGER* and *ACTB* (Table 2) in SN harvested from each group (Fig. 1A) were analyzed in duplicates by LightCycler® 480 Instrument II (Roche Diagnostics, Switzerland). The quantitative PCR (qPCR) reaction included the following: 100 ng cDNA template, 12 μl TaqMan^®^ Universal Master Mix II, with UNG (Life Technologies, Grand Island, NY, USA), and 1 μl each of four TaqMan^®^ Gene Expression Assays (Table 2, Life Technologies, Grand Island, NY, USA) as well as RNase-free water to a final volume of 20 μl. Non‐template controls were used for each probe to confirm reaction specificity. The relative amplification of genes was calculated using the *ΔΔ*Ct method and normalized using the geometric mean of the expression levels of high stability reference gene in mouse SN, *i.e.* *18S rRNA* (Table 2).

*Localization and immunoreactivity of Diaph1, RAGE and ACTB proteins*

The presence of Diaph1, RAGE and ACTB in SN sections was determined using a two-day procedure for semi-quantitative immunohistochemical (IHC) staining. Tissue samples were cut on a CM1800 cryostat at -20°C (Leica, Wetzlar, German) into 8-µm sections and mounted onto poly-l-lysine-coated glass microscope slides (Menzel-Glaser, Braunschweig, Germany). IHC analysis was performed as described in a protocol of VECTASTAIN® ABC-HRP Kit, Peroxidase (Rabbit IgG, PK-4001; Vector Laboratories, USA). SN sections were incubated with primary antibodies (Table 3) diluted in 0.1% BSA at 4°C, overnight. To visualize the immunoreactivity, sections were immersed in 3,3 diaminobenzidine tetrahydrochloride (DAB, Dako, USA) followed by hematoxylin stained (Aqua-Med, Poland). Subsequently, SN sections were dehydrated in graded series of ethanol (70, 90, 100%), cleared in xylene, and mounted with DPX (Sigma Aldrich, USA).

Stained tissue sections were photographed using Olympus BX51 Trinocular Transmitted Light Microscope (Olympus, Japan). Images were taken under 40× objective with 0.75 numerical aperture (40×/0.75). Areas of staining were determined with ImageJ software which automatically converted the positive signal of immunoreaction into the corresponding range of gray values. The total percentage of staining immunoreactive area was calculated per ROI for four technical replicates per each of biological replicates (number of samples) within each group of animals.

*The content of Diaph1, profilin 1 (PFN1), N(epsilon)-(carboxymethyl)lysine-AGEs (CML–AGEs), High Mobility Group Box 1 (HMGB1), S100 Calcium Binding Protein B (S100B), S100 Calcium Binding Protein A6 (S100A6), superoxide dismutase type 1 (SOD1), ACTB and RAGE proteins in SN*

*Protein extraction*

Whole SN (min. n = 6 per group; at 6 months of experiment) was homogenized with isolation buffers (Eurx^®^, Poland) in MagNA Lyser (Roche Diagnostics, Switzerland) according to the manufacturer’s protocol (Universal DNA/RNA/Protein Purification Kit, Eurx^®^). The protein concentration was determined using Direct Detect® Infrared Spectrometer for Total Protein Quantitation (Merck Millipore, Darmstadt, Germany).

*Western blot analysis*

Approximately 40 µg of total protein was separated on 15-well Mini-PROTEAN® TGX™ (Tris-Glycine eXtended) Precast Protein Gels (Bio-Rad, Hercules, CA, USA) and transferred onto nitrocellulose membrane using semi-dry system, *i.e.* Trans-Blot Turbo Transfer System (Bio-Rad). Subsequently, the blotting membrane was incubated in EveryBlot Blocking Buffer (Bio-Rad) for 5 min at room temperature (RT). Primary antibody solutions (Table 3) were diluted in SignalBoost™ Immunoreaction Enhancer solution (Merck Millipore, Darmstadt, Germany) and left overnight in 4 ℃. Then, the membrane was washed in PBS with 0,1% Tween-20 and incubated for 2 h at RT with the secondary antibody (Table 3) diluted in SignalBoost™ Immunoreaction Enhancer solution (Merck Millipore, Darmstadt, Germany). Next, bands were visualized with ChemiDoc Imaging Systems (Bio-Rad Hercules, CA, USA). Images were quantified densitometrically with Image Lab v6.0.1 software and compared to experimental condition after normalization to total amount of protein loaded in the gel (https://www.bio-rad.com/en-pl/applications-technologies/total-protein-normalization?ID=PODYJQRT8IG9).

*Co-localization of Diaph1 and ACTB as well as ACTB and PFN1 in SN*

*Immunofluorescence*

To investigate the co-expression and co-localization between Diaph1 and ACTB as well as ACTB and PFN1 in mouse SN, samples were collected six months after induction of diabetes (Fig. 1A). Immunofluorescence staining was performed according to standard laboratory protocols. Briefly, SN samples were cut longitudinally at 8 µm thickness on a cryostat (Leica, Wetzlar, German) and mounted onto slides (SuperFrost Plus; Fisher Scientific, Pittsburgh, PA, USA). Next, SN sections were incubated with 2.5% goat normal serum (Sigma Aldrich, USA). Consequently, sections were incubated with respective primary antibodies diluted in 0.1% BSA at 4°C, overnight (Table 3). To visualize the immunofluorescence staining, SN sections were immersed with secondary antibodies diluted in 0.1% BSA for 1 h at RT (Table 3). Samples were mounted with glycerol in PBS (pH 8.4, Euroimmun, PerkinElmer Germany Diagnostics GmbH) and subsequently examined using a microscope (Olympus IX83, Japan) with ORCA-Flash4.0 V3 Digital CMOS camera: C13440-20CU (Hamamatsu, Japan). Representative pictures were taken under magnification ×40 with 0.6 numerical aperture (40×/0.6). Areas of fluorescent staining were determined with CellSens Imaging Software v. 2.3 (Olympus). Negative control (NC) was used for each primary antibodies to confirm immunofluorescent staining specificity.

*Next-generation sequencing procedure*

The next-generation sequencing workflow include four basic steps: 1) sample preparation, 2) library construction, 3) sequencing, 4) raw data analysis.

*Sample preparation*

Total RNA was extracted from lumbar SC neuromere (n = 5 per group) harvested from 32-weeks-old mice. RNA was isolated using RNeasy® Plus Universal Mini Kit and QIAzol® Lysis Reagent (both Qiagen, USA), according to manufacturer’s recommendations. Briefly, tissue samples were homogenized in QIAzol® Lysis Reagent (Qiagen, USA). After addition of gDNA Eliminator Solution and chloroform, homogenate was separated into aqueous and organic phases by centrifugation. The upper, aqueous phase was mixed with ethanol to provide appropriate binding conditions and applied to RNeasy Mini spin column (Qiagen, USA). Total RNA was bound to the spin column membrane, and phenol and other contaminants were efficiently washed away. High-quality RNA was then eluted in RNase-free water (Qiagen, USA). Subsequently, RNA integrity (RIN), quality and quantity were evaluated with microcapillary electrophoresis (2100 Bioanalyzer, Agilent Technologies, Santa Clara, CA, USA). Only samples with RIN above 8 were used for further analysis.

*Library construction*

Library was constructed using TruSeq stranded mRNA kit (Illumina®, USA). The sequencing library was prepared by random fragmentation of the cDNA sample, followed by 5' and 3' adapter ligation. Polymerase chain reaction (PCR) amplification was performed to enrich cDNA libraries. Subsequently, the sample quality was checked according to the Illumina qPCR Quantification Protocol Guide (USA). Standard curve of fluorescence readings and library sample concentration were generation with Roche's Rapid library standard Quantification solution and Calculator (Switzerland).

*Sequencing*

The sequencing was performed on the NovaSeq 6000 platform (Illumina®, USA) to generate 2 × 150 bp paired-end reads, with assumed min. sequencing depth – 40 million total reads per sample.

*Raw data analysis*

Sequencing data was converted into raw data for the *in silico* analysis (Supplementary Fig. 1). Firstly, in order to verify the integrity and quality of files with raw data, md5sum and FastQC v0.11.8 software was used. The md5sum is designed to verify data integrity using Message Digest Algorithm 5 (MD5). Consequently, regions of row reads were trimmed with use of Trimmomatic v0.38 program. Next, mapping was performed using STAR tool. For mapping the *Mus musculus* GRCm39 was used as the reference genome with annotation version GRCm39.104 downloaded from Ensembl database (https://www.ensembl.org/index.html). Principal component analysis (PCA) and Euclidean distances between samples analysis were performed using ggplot2 library v3.3.5 (https://www.rdocumentation.org/packages/ggplot2/versions/3.3.5).

*Functional annotation of DEGs*

Functional analysis was performed for genes with significant expression differences with The Database for Annotation, Visualization and Integrated Discovery (DAVID) v6.8. DAVID 6.8 provides a comprehensive set of functional annotation tools for investigators to understand biological meaning behind large list of genes. The list of DEGs was uploaded to DAVID 6.8 to identify enriched biological themes, particularly Gene Ontology (GO) terms and visualize KEGG pathways. The GeneMANIA Prediction Server was used to create an interaction network of selected genes among DEGs in lumbar SC of diabetic mice with *AGER, Diaph1* and *ACTB*.

*Validation of selected genes with altered expression in lumbar SC neuromere harvested in six months of diabetes*

Validation of selected genes (Table 1) with altered expression in lumbar SC harvested from diabetic mice (Fig. 1) were performed by using quantitative PCR (qPCR). The qPCR was proceeded by cDNA synthesis (reverse transcription) using the same RNA as in the library construction. The expression of hydroxyacid oxidase 1 (*HAO1*)*,* neuroepithelial cell transforming 1 (*NET1*)*,* ras homolog family member J (*RHOJ*)*,* thioredoxin interacting protein (*TXNIP*), cathepsin E (*CTSE*) was investigated in duplicate by LightCycler® 480 Instrument II (Roche Diagnostics, Switzerland). The qPCR reaction included the following: 40 ng cDNA template, 10 ul Power SYBR™ Green PCR Master Mix (Life Technologies, Grand Island, NY, USA), 1 μl each of primers (Table 1) and RNase-free water to a final volume of 20 μl. The relative amplification of genes was calculated using the *ΔΔ*Ct method and normalized using the geometric mean of the expression levels of high stability reference genes in mice SC, *i.e.* 18S ribosomal RNA (*18S rRNA,* Table) and importin 8 (*IPO8*, Table 1).

*The comparison of SC and SN transcriptomes in diabetes – Venn diagram*

The obtained list of DEGs with a known biological function in SC of T1D was compared with the list of DEGs with known biological functions in T1D and T2D SN obtained from Gene Expression Omnibus (GEO) database (<http://www.ncbi.nlm.nih.gov.geo/>). The comparison of transcriptomes was performed by constructing a Venn diagram.

*Statistical analyses*

Analyses with *P* values ≤ 0.05 were considered as statistically significant. All data were presented as mean ± *SEM*. Before selecting the appropriate statistical test, we have performed the normality and lognormality test, *i.e.* *Shapiro-Wilk* test. Moreover, all datasets were tested for presence of outliers by using the Grubbs’ test (α = 0.05). Statistical analyses and graphs were performed using GraphPad Prism 9.1.0. (CA, USA). The expression of selected genes with altered expression in lumbar SC harvested from six-month diabetic mice was compared with *Student's t*-test or in case of abnormal data distribution nonparametric equivalent, *i.e.* *U Mann-Whitney* test. The morphometric analysis of semithin sections, measured immunoreactivity as well as relative presence of protein in diabetic as well as control SN were compared with *Student's t*-test or in case of abnormal data distribution nonparametric test, *i.e.* *U Mann-Whitney* test. Moreover, the *Student's t*-test or *U Mann-Whitney* test was used to determine the individual changes in the *Diaph1, AGER* and *ACTB mRNAs* expression level in SN. *One-way* ANOVA followed by Tukey's HSD post hoc test was used to indicate the effect of diabetes on alterations in MNCV and SNCV. Finally, in case of morphometric analysis, MNCV and SNCV *two-way* ANOVA was performed to evaluate main effects and interactions among factors (diabetes status and months of diabetes) followed by the Tukey’s HSD post-hoc test. The effect on diabetes on alternations in body mass and blood glucose level were defined by Kruskal-Wallis test.
